# Discovery and functional characterization of a bombesin-type neuropeptide signaling system in an invertebrate

**DOI:** 10.1073/pnas.2420966122

**Published:** 2025-03-28

**Authors:** Weiling Huang, Xingxing Zhong, Cleidiane G. Zampronio, Andrew R. Bottrill, Kite G. E. Jones, Ana B. Tinoco, Lijin Guo, Michaela Egertová, Olivier Mirabeau, Maurice R. Elphick

**Affiliations:** ^a^Centre for Evolutionary and Functional Genomics, School of Biological and Behavioural Sciences, Queen Mary University of London, London E1 4NS, United Kingdom; ^b^Proteomics Facility Research Technology Platform, School of Life Sciences, University of Warwick, Coventry CV4 7AL, United Kingdom; ^c^College of Animal Science, South China Agricultural University, Guangzhou 510642, China; ^d^Brain-Immune Communication Lab, Institut Pasteur, Université Paris Cité, Bioinformatics and Biostatistics Hub, Inserm U1224, Paris 75015, France

**Keywords:** neuropeptide, G protein–coupled receptor, bombesin, echinoderm, *Asterias rubens*

## Abstract

Neuropeptide signaling systems are key regulators of physiological and behavioral processes in animals. However, the evolutionary history of some neuropeptides originally discovered in vertebrates is unknown. The peptide bombesin (BN) was first isolated from the skin of the toad *Bombina bombina* and subsequently BN-related neuropeptides have been identified in other chordates, including gastrin-releasing peptide (GRP) and neuromedin B (NMB) in mammals, and a GRP-like peptide in the cephalochordate *Branchiostoma japonicum*. However, BN-type neuropeptides have hitherto not been identified in any nonchordate animals. Here, we report the discovery and functional characterization of a BN-type neuropeptide signaling system in an echinoderm—the starfish *Asterias rubens*. BN-type precursor proteins were identified in several echinoderm species based on their amino acid sequences and gene structures, and the mature structure of the *A. rubens* BN-type neuropeptide ArBN was determined using mass spectrometry. A protein related to vertebrate GRP/NMB-type G protein–coupled receptors was identified experimentally as the receptor for ArBN in *A. rubens*. Analysis of the distribution of the ArBN precursor in *A. rubens* using mRNA in situ hybridization and immunohistochemistry revealed a widespread pattern of expression in the central nervous system, digestive system, and locomotory organs. Moreover, effects of ArBN in *A. rubens* included contraction and retraction of the evertible stomach and inhibition of feeding behavior. Our findings show that the evolutionary history of BN-type neuropeptide signaling can be traced back to the deuterostome common ancestor of echinoderms and chordates. Furthermore, an ancient role of BN-type neuropeptides as regulators of feeding behavior has been revealed.

## Significance

The peptide bombesin (BN) was first discovered in frog skin and subsequently the BN-related neuropeptides gastrin-releasing peptide and neuromedin B were identified in mammals and other vertebrates as regulators of feeding, digestion, and other physiological processes. Hitherto, BN-type peptides and their cognate G protein–coupled receptors have only been identified in chordates. Here, we report the discovery and functional characterization of a BN-type signaling system in a nonchordate deuterostome invertebrate—the starfish *Asterias rubens* (phylum Echinodermata). Our findings demonstrate the evolutionary origin of BN-type signaling in the deuterostome branch of the animal kingdom and uncover an ancient role in regulation of feeding behavior.

Neuropeptides are evolutionarily ancient signaling molecules that are secreted by neurons and regulate diverse physiological and behavioral processes in animals (1, 2). Neuropeptides typically exert effects by binding to G protein–coupled receptors (GPCRs) and the coevolution of neuropeptides and their receptors has been revealed by phylogenetic analysis of transcriptome/genome sequence data. Furthermore, the evolutionary origin of many neuropeptide-receptor signaling systems can be traced back to a common ancestor of bilaterian animals (1, 3, 4). Reconstruction of the evolution of neuropeptide signaling systems has been facilitated by research on well-established model systems such as the insect *Drosophila melanogaster* (phylum Arthropoda) and *Caenorhabditis elegans* (phylum Nematoda) (2, 5, 6). However, more recently the importance of research on invertebrates belonging to other phyla has become apparent. For example, identification of neuropeptide signaling systems in an invertebrate deuterostome, the starfish *Asterias rubens* (phylum Echinodermata) (1, 7, 8), has provided key insights into the evolution of gonadotropin-releasing hormone/corazonin-type, prolactin-releasing peptide/short neuropeptide F-type and somatostatin/allatostatin C-type signaling systems (9–11). Accordingly, in this paper, we report use of *A. rubens* as an experimental system to investigate the evolution of bombesin-type neuropeptide signaling.

The peptide bombesin (BN; pQQRLGNQWAVGHLM-NH_2_) was first isolated from the skin of the frog *Bombina bombina* (12, 13). Furthermore, related peptides were identified in other frog species, including ranatensin (pQVPQWAVGHFM-NH_2_) from *Rana pipiens* (14) and phyllolitorin (pQLWAVGSLM-NH_2_) from *Phyllomedusa sauvagii* (15), revealing sequence similarity in their C-terminal regions. A BN-like peptide was identified in the porcine gut and named gastrin-releasing peptide (GRP) on account of its first known bioactivity in stimulating gastrin secretion (16, 17). Then in 1983, a ranatensin-like peptide was identified in the porcine spinal cord and named neuromedin B (NMB) (18). Thus, a family of BN-related peptides has been identified in vertebrates, with both GRP-type and NMB-type neuropeptides being discovered in nonmammalian vertebrates (19).

BN-type neuropeptides exert their effects by binding to GPCRs. In mammals, NMB and GRP act as high affinity ligands for the BB1 receptor (NMB-R) and the BB2 receptor (GRP-R), respectively, and orthologs of these receptors have been identified in nonmammalian vertebrates (20–24). Furthermore, a third receptor type that is activated by BN-type peptides has been identified in vertebrates more recently and is known as BRS-3 or the BB4 receptor (24–26). BN-type neuropeptides are involved in regulation of a variety of physiological and behavioral processes in mammals/vertebrates, including the release of gastrointestinal hormones, smooth muscle contraction in the stomach and small intestine, suppression of food intake, mediation of satiety, regulation of circadian rhythms, and learning and enhancement of fear memories (19, 27–35). Consistent with these diverse roles, BN-type neuropeptides are widely expressed in the central nervous system and peripheral organs of mammals and other vertebrates (36, 37).

Insights into the evolutionary history of BN-type neuropeptide signaling have been obtained from phylogenetic analysis of genome sequence data. A gene encoding a BN-type neuropeptide precursor was identified in the invertebrate chordate *Branchiostoma floridae* [subphylum Cephalochordata; (4)] and, subsequently, experimental characterization of BN-type neuropeptide signaling in the cephalochordate *Branchiostoma japonicum* has been reported (24). A GPCR that is activated by the BN-type neuropeptide DKGQEHWQYGHWY-NH_2_ was identified. Furthermore, expression of the gene encoding this neuropeptide was found to be affected by temperature and light. Thus, the evolutionary history of BN-type neuropeptide signaling has been traced back to the common ancestor the chordates. However, BN-type neuropeptides have yet to be identified in nonchordates.

The aim of this study was to investigate the occurrence of BN-type neuropeptide signaling in a nonchordate deuterostome phylum—the echinoderms. Specifically, we report the discovery and functional characterization of a BN-type neuropeptide signaling system in the starfish *A. rubens*, providing important insights into the evolution and comparative physiology of BN-type neuropeptides and their receptors.

## Results

### Identification of Putative Neuropeptide Precursors in *A. rubens* and Other Echinoderms that Share Sequence Similarity with BN-Type Peptides in Chordates.

**Fig. 1.**
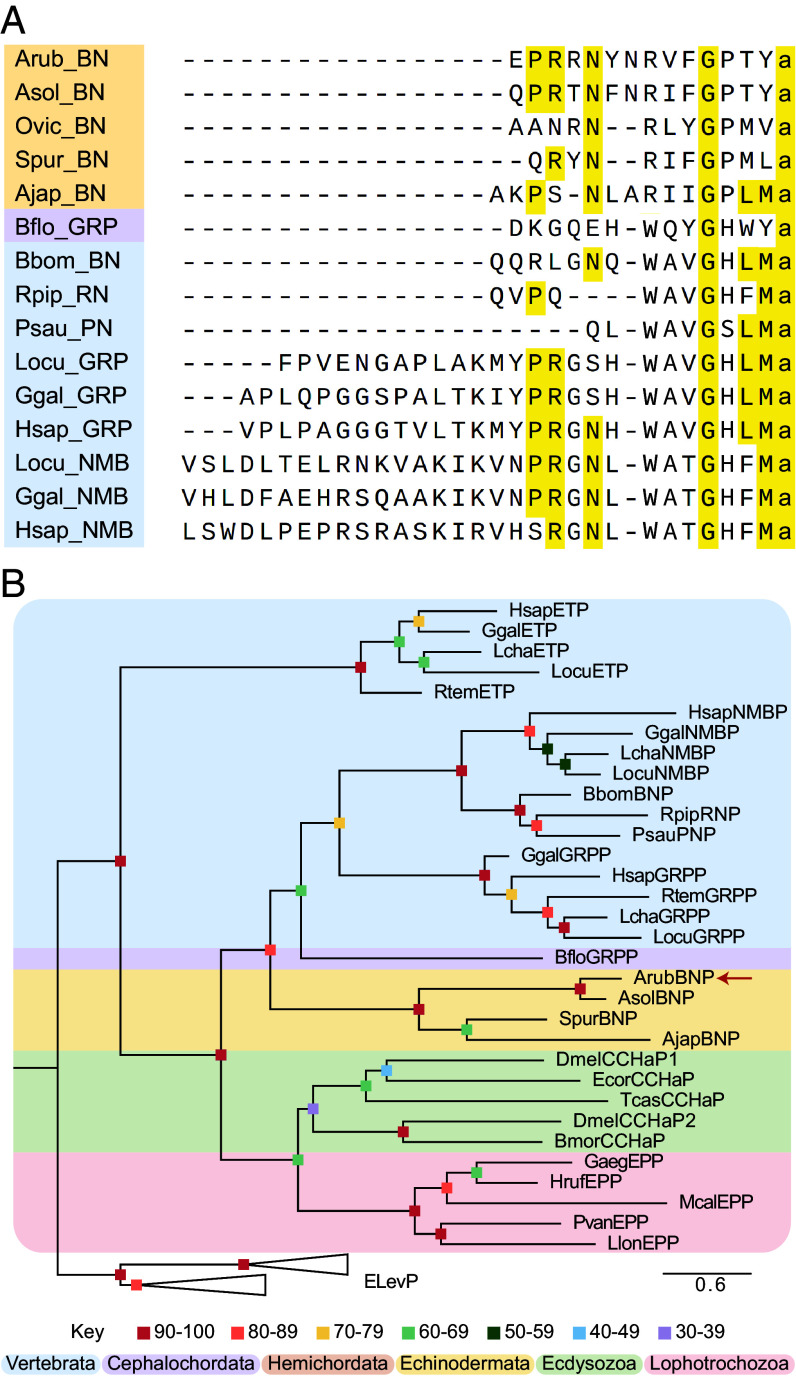
Sequence alignment and phylogenetic analysis of putative echinoderm and chordate BN-type peptides/precursors. (*A*) Alignment of putative echinoderm BN-type neuropeptides with chordate BN-type neuropeptides. Conserved residues that occur in at least one echinoderm species and several chordate species are highlighted in yellow. Species and peptide names are highlighted in taxon-specific colors: yellow (Echinodermata), purple (Cephalochordata), blue (Vertebrata). Species name abbreviations: Ajap (*A. japonicus*), Asol (*Acanthaster* cf. *solaris*), Arub (*A. rubens*), Bbom (*B. bombina*), Bflo (*B. floridae*), Ggal (*Gallus gallus*), Hsap (*Homo sapiens*), Locu (*Lepisosteus oculatus*), Ovic (*Ophionotus victoriae*), Psau (*Phyllomedusa sauvagii*), Rpip (*R. pipiens*), Spur (*Strongylocentrotus purpuratus*). Peptide abbreviations: BN (Bombesin), RN (Ranatensin), PN (Phyllolitorin), GRP (Gastrin-releasing peptide), NMB (Neuromedin B). Accession numbers of the precursor sequences used for the neuropeptide alignment in this figure are listed in *SI Appendix,* Table S1. (*B*) Phylogenetic tree generated using the maximum likelihood method (1,000 bootstrap replicates, JTT+I+G4 substitution model) and showing that the putative *A. rubens* BN-type precursor (ArubBNP) (arrow) and related proteins in other echinoderms are positioned in the same clade as chordate BN-type precursors. This clade is distinct from other clades that comprise protostome CCHa/EP-type precursors and vertebrate ET-type precursors. Elevenin-type precursors are included as an outgroup. Bootstrap support for each node is represented by colored squares and the colored backgrounds highlight different taxonomic groups (see key). The scale bar represents the average residue substitution per site. Abbreviations for species names not shown in part A of this figure: Bmor (*Bombyx mori*), Dmel (*D. melanogaster*), Ecor (*Eupeodes corollae*), Gaeg (*Gigantopelta aegis*), Hruf (*Haliotis rufescens*), Lcha (*Latimeria chalumnae*), Llon (*Lineus longissimus*), Mcal (*Mytilus californianus*), Pvan (*Perinereis vancaurica*), Rtem (*Rana temporaria*), Tcas (*Tribolium castaneum*). Accession numbers of precursor sequences used to generate this phylogenetic tree are listed in *SI Appendix,* Table S2.

Analysis of transcriptome/genome sequence data identified putative neuropeptide precursor proteins in *A. rubens* and other echinoderms that share sequence similarity with chordate BN-type peptides. Specifically, the similarity resides in a putative neuropeptide sequence located adjacent to the predicted N-terminal signal peptide. In Fig. 1*A*, the sequences of the putative BN-type neuropeptides from *A. rubens* (ArBN) and other echinoderms are aligned with the sequences of chordate BN-type peptides. This reveals that the N-terminal PRXN motif in several of the echinoderm peptides and a C-terminal GXLMa motif in the *Apostichopus japonicus* peptide are features that are shared with several chordate BN-type peptides, where X is variable and “a” is a C-terminal amide predicted to be derived from glycine in the precursor proteins. Furthermore, the predicted amidated tyrosine (Ya) in the *A. rubens* peptide is also a characteristic of the GRP-like peptide in the cephalochordate *B. floridae*. In addition, an alignment of the sequences of the putative BN-type neuropeptides from *A. rubens* and other echinoderm species is shown in *SI Appendix,* Fig. S1, revealing a consensus N-terminal PRXN motif and a consensus C-terminal RIFGPXXa motif. Collectively, these sequence similarities suggest that the putative neuropeptide precursors identified in echinoderms are orthologs of BN-type peptide precursors in chordates. However, because sequence similarity is limited to only a few residues, we sought further evidence of orthology.

### Phylogenetic Analysis of Relationships Among BN-Type, ET-Type, CCHa/EP-Type Peptide Precursors in Different Taxa.

Using a maximum-likelihood-based phylogenetic method, we investigated relationships of putative BN-type precursors in echinoderms with BN-type precursors in chordates. Included in this analysis were precursors of peptides that act as ligands for GPCRs that have been shown to be closely related to BN-type receptors, which include precursors of endothelin (ET)-type peptides in vertebrates, precursors of CCHamide (CCHa)-type peptides in ecdysozoan protostomes and precursors of the CCHa-like excitatory peptides (EPs) in lophotrochozoan protostomes. Elevenin-type precursors were also included as an outgroup to root the tree. Importantly, the phylogenetic tree generated shows that BN-type precursors in *A. rubens* (ArubBNP) and other echinoderms are positioned in a clade together with chordate BN-type precursors (Fig. 1*B*). This provides further evidence that the putative BN-type precursors in echinoderms are orthologs of BN-type precursors in chordates.

### Similarity in the Exon/Intron Structure of Genes Encoding Putative BN-Type Precursors in Echinoderms and BN-Type Precursors in Chordates.

**Fig. 2.**
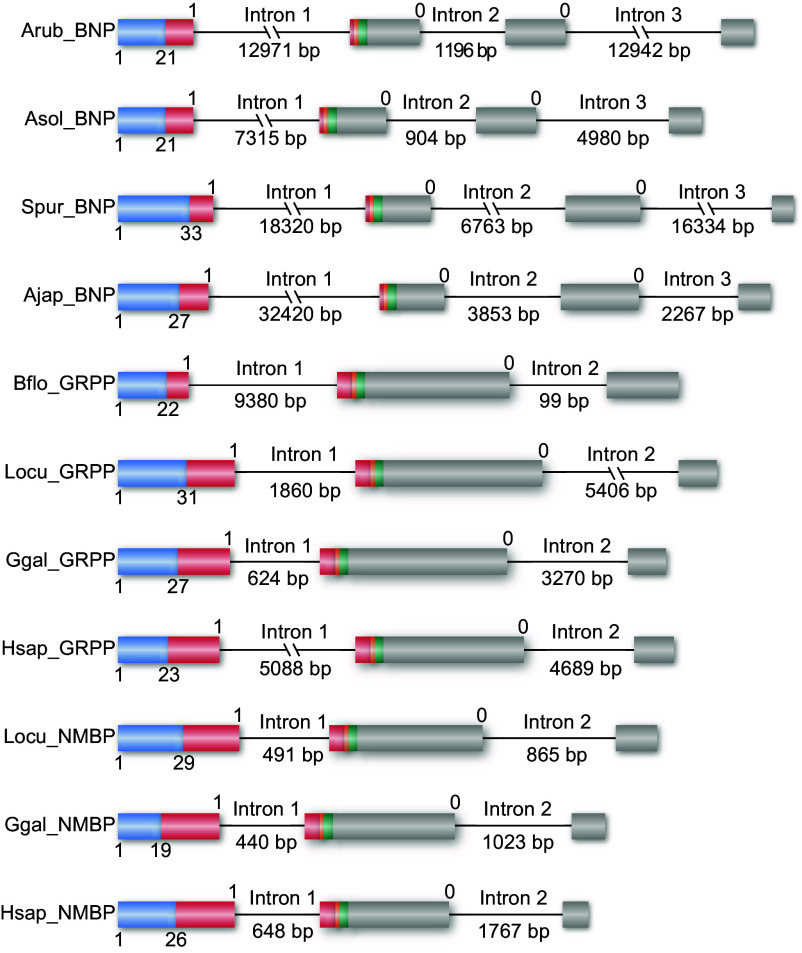
Comparison of the exon/intron structure of genes encoding precursors of putative BN-type peptides in echinoderms and BN-type peptides in chordates. Exons are color-coded to show the N-terminal signal peptide (blue), BN-type peptide (red), C-terminal glycine predicted to be a substrate for amidation (orange), cleavage sites (green), and other regions (gray). Protein-coding exons are shown as rectangles and introns as lines (with length stated underneath). A conserved phase 1 intron (intron 1) interrupts the neuropeptide-encoding sequences and a conserved phase 0 intron (intron 3 in echinoderms, intron 2 in chordates) interrupts the coding sequences for the C-terminal region of the precursor proteins. Intron 2 (phase 0) in echinoderms is unique to this phylum. Full names of species: Ajap (*A. japonicus*), Arub (*A. rubens*), Asol (*A.* cf. *solaris*), Bflo (*B. floridae*), Ggal (*G. gallus*), Hsap (*H. sapiens*), Locu (*L. oculatus*), Spur (*S. purpuratus*). Full names of peptide precursor genes: BNP (Bombesin precursor), GRPP (Gastrin-releasing peptide precursor), NMBP (Neuromedin B precursor). Accession numbers of the precursor cDNAs and corresponding genomic sequences shown in Fig. 2 are listed in *SI Appendix,* Table S3. An alignment that shows the positions of introns with respect to the precursor amino acid sequences is shown in *SI Appendix*, Fig. S2.

To further investigate a relationship between the putative BN-type precursors in echinoderms and BN-type precursors in chordates, the exon/intron structure of the genes encoding these proteins was compared (Fig. 2). Chordate BN-type precursor genes typically have two introns interrupting the protein-coding sequence, with the first a phase 1 intron that interrupts the coding sequence for the BN-type peptide and the second a phase 0 intron that interrupts the coding sequence for the C-terminal region of the precursor protein. Genes encoding the putative precursors of BN-type neuropeptides in echinoderms all have three introns that interrupt the protein-coding sequence and, importantly, the location and phases of the first and third introns are consistent with those in chordate BN-type precursor genes (Fig. 2 and *SI Appendix,* Fig. S2). In particular, it is noteworthy that the first intron (phase 1) interrupts the coding sequence for the putative neuropeptide in the echinoderm genes, consistent with the occurrence of this characteristic in chordate BN-type precursor genes. In contrast, the second intron (phase 0) was only found in the putative BN-type precursor genes in echinoderms, indicating that this intron originated since the divergence of echinoderms and chordates from a common ancestor. Importantly, the similarities in the structure of the genes encoding the putative BN-type precursors in echinoderms and BN-type precursors in chordates provide strong supporting evidence of orthology.

### Identification of Peptides Derived from the BN-Type Precursor in the Starfish *A. rubens*.

Having obtained evidence that a putative BN-type precursor in the starfish *A. rubens* (ArBNP; GenBank accession no. XM_033768834.1) is an ortholog of chordate BN-type precursors, we cloned and sequenced a cDNA encoding this protein to confirm the sequence determined from analysis of *A. rubens* transcriptome/genome sequence data (*SI Appendix,* Fig. S3). This revealed that ArBNP is a 102-residue protein comprising a predicted 21-residue N-terminal signal peptide, from which three potential neuropeptides could be derived based on analysis of its sequence: i) a C-terminally amidated 14-residue peptide (ArBN, EPRRNYNRVFGPTY-NH_2_) (*SI Appendix,* Fig. S4*A*); ii) a C-terminally amidated 10-residue peptide corresponding to the C-terminal region of ArBN (ArBN_5-14_; NYNRVFGPTY-NH_2_) (*SI Appendix,* Fig. S4*B*); iii) a C-terminally amidated 16-residue peptide predicted to be derived from residues 65 to 81 of ArBNP (ArBNP_65-81_; EPMPSSLALYIANLSP-NH_2_) (*SI Appendix,* Fig. S4*C*). Having synthesized these as reference peptides, mass spectrometric analysis of an *A. rubens* radial nerve cord extract revealed the presence of ArBN and ArBN_5-14_, but not ArBNP_65-81_ (*SI Appendix,* Fig. S4). Consistent with these findings, it is noteworthy that the ArBN sequence is highly conserved in the *A*. cf. *solaris* BN-type precursor, whereas the ArBNP_65-81_ sequence is not highly conserved in the *A*. cf. *solaris* BN-type precursor (*SI Appendix,* Fig. S2).

### Identification of BN-Type Receptors in *A. rubens*.

Having identified ArBN and ArBN_5-14_ as peptides that are naturally derived from ArBNP in *A. rubens*, we then sought to identify receptors that these peptides may act as ligands for. BLAST analysis of transcriptome/genome data identified eleven candidate BN-type receptors, which we refer to as ArBNR1-11. Homologs of this family of proteins were also identified in other echinoderms. Analysis of the sequences of ArBNR1-11 using the protein topology visualization tool Protter indicated that all of these proteins, except ArBNR2, have seven predicted transmembrane domains, as expected for GPCRs (*SI Appendix,* Fig. S5). Analysis of the sequence of ArBNR2 revealed that it has a glutamate residue at position 272, whereas the residue occupying this position in an ortholog of ArBNR2 in the starfish *Marthasterias glacialis* (MgBNR2) is glycine, and MgBNR2 has seven predicted transmembrane domains. Accordingly, a modified form of ArBNR2 in which the glutamate residue at position 272 is substituted with glycine, ArBNR2(G_272_), also has seven predicted transmembrane domains (*SI Appendix*, *SI Methods*).

**Fig. 3.**
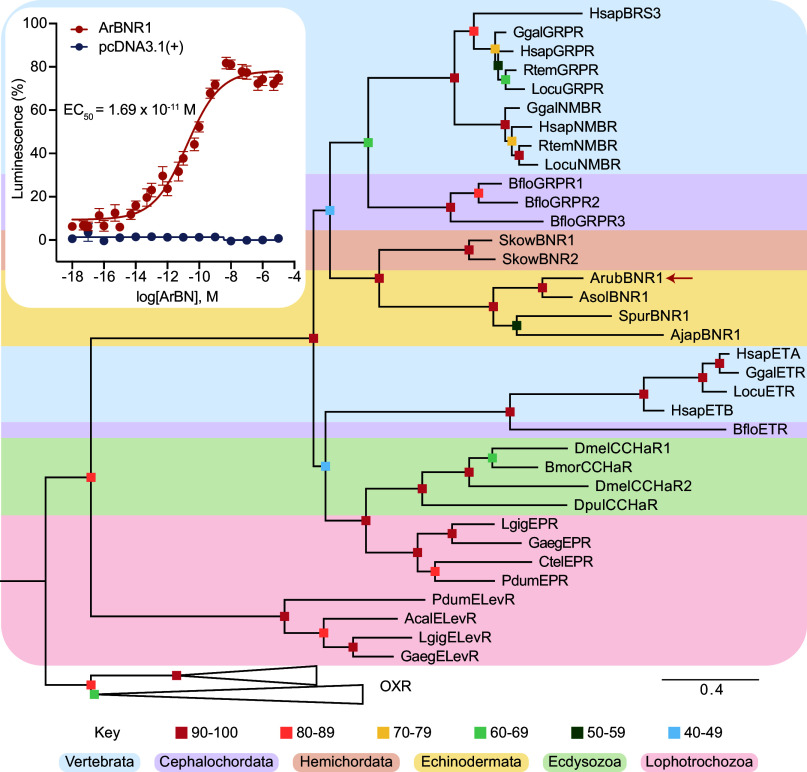
Phylogenetic and functional identification of the *A. rubens* receptor ArBNR1 as a BN-type receptor. The phylogenetic tree was generated using the maximum likelihood method (1,000 bootstrap replicates, LG+F+G4 model) and it shows that ArBNR1 (arrow) is positioned in a clade that contains chordate BN-type receptors and that is distinct from clades comprising chordate ET-type receptors, protostome CCHa/EP-type receptors, and protostome elevenin-type receptors. Orexin-type receptors were included as an outgroup to root the tree. The colored squares represent bootstrap support for clades and colored backgrounds highlight different taxonomic groups (see key). The scale bar represents the average residue substitution per site. Abbreviations for species names: Acal (*Aplysia californica*), Ajap (*A. japonicus*), Arub (*A. rubens*), Asol (*A.* cf. *solaris*), Bflo (*B. floridae*), Bmor (*B. mori*), Ctel (*Capitella teleta*), Dmel (*D. melanogaster*), Dpul (*Daphnia pulex*), Gaeg (*G. aegis*), Ggal (*G. gallus*), Hsap (*H. sapiens*), Lgig (*Lottia gigantea*), Locu (*L. oculatus*), Pdum (*Platynereis dumerilii*), Rtem (*R. temporaria*), Skow (*S. kowalevskii*), Spur (*S. purpuratus*). Accession numbers for the receptor sequences used to generate this tree are listed in *SI Appendix,* Table S4. The inset shows that the peptide ArBN (EPRRNYNRVFGPTY-NH_2_) acts as a ligand for ArBNR1, triggering dose-dependent luminescence in Chinese hamster ovary (CHO)-K1 cells coexpressing ArBNR1 and Gqs5, with half-maximal response concentration (EC_50_) of 1.69 × 10^−11^ M. No ArBN-induced luminescence is observed in control experiments where cells were cotransfected with empty pcDNA3.1(+) vector and Gqs5. Each point represents mean values ± SEM from at least four independent experiments performed in triplicate (Dataset S1). Luminescence is expressed as a percentage of the maximal response observed in each experiment.

Use of CLANS to investigate relationships of ArBNR1-11 with BN-type receptors in other taxa and closely related GPCRs (ET-type, CCHa/EP-type, ELev-type) revealed that ArBNR1-9 are positioned in a cluster comprising BN-type, ET-type, and CCHa/EP-type receptors, whereas ArBNR10 and ArBNR11 are more distal to this cluster (*SI Appendix,* Fig. S6). A phylogenetic analysis of the same receptor sequences was performed (*SI Appendix,* Fig. S7*A*) and this revealed that ArBNR1-11 and related receptors in other echinoderms are all positioned in a clade that is distinct from a clade comprising chordate BN-type receptors, chordate ET-type receptors, and protostome CCHa/EP-type receptors. However, it is noteworthy that the branch leading to ArBNR1 is shorter than branches leading to ArBNR2-11. Because inclusion of a large number of echinoderm receptors in our dataset may have affected the topology of the tree, we also generated trees using smaller subsets of the echinoderm receptors. In this analysis, ArBNR1 and orthologous receptors in other echinoderms were positioned in a clade together with chordate BN-type receptors and putative BN-type receptors in the hemichordate *Saccoglossus kowalevskii* (Fig. 3). In contrast, all of the other candidate BN-type receptors in *A. rubens* were positioned in a clade that was distinct from a clade comprising chordate BN-type receptors, chordate ET-type receptors, and protostome CCHa/EP-type receptors (*SI Appendix, Fig.* S7 *B–H*). Collectively, these findings indicated that ArBNR1 is the strongest candidate receptor for the BN-type peptides ArBN and/or ArBN_5-14_ in *A. rubens*.

### ArBN, but Not ArBN_5-14_, Acts as a Ligand for ArBNR1.

Having identified ArBNR1 as the strongest candidate receptor for ArBN and/or ArBN_5-14_ in *A. rubens*, we performed in vitro experiments to test this potential ligand–receptor partnership. Furthermore, because analysis of receptor relationships using CLANS indicated that ArBNR2 and ArBNR3, like ArBNR1, may share more similarity with chordate BN-type receptors than other GPCRs in *A. rubens*, we also tested ArBN and ArBN_5-14_ as ligands for ArBNR2 and ArBNR3. In addition, although the putative C-terminally amidated peptide ArBNP_65-81_ was not detected in *A. rubens*, we also tested this peptide to investigate whether it could act as a ligand for ArBNR1-3. A modified form of ArBNR2 (ArBNR2(G_272_)) was also tested in these experiments, as explained in *SI Appendix*, *SI Methods*.

ArBN was found to cause dose-dependent luminescence in CHO-K1 cells transfected with ArBNR1 and the chimeric G protein Gqs5, with a half-maximal response concentration (EC_50_) of 1.69 × 10^−11^ M (Fig. 3, *Inset*). Furthermore, the mean total luminescence response triggered by 10^−5^ M ArBN in cells expressing ArBNR1 was 147.08 × 10^4^, which was an order of magnitude greater than the mean total luminescence observed when cells expressing ArBNR1 were exposed to the media used to dissolve ArBN (16.29 × 10^4^) (*SI Appendix,* Fig. S8). ArBN_5-14_ and ArBNP_65-81_ were also tested as ligands for ArBNR1 but peptide-induced luminescence was only observed with ArBN_5-14_ (*SI Appendix,* Fig. S9 *B* and *C*)_._ However, this was only observed at the highest concentration tested (10^−5^ M) and with low levels of luminescence that were only slightly higher than observed with the vehicle media (*SI Appendix,* Fig. S9*B*). Furthermore, ArBN, ArBN_5-14_, and ArBNP_65-81_ did not trigger luminescence in cells expressing the other receptors tested (ArBNR2, ArBNR2(G_272_), or ArBNR3) (*SI Appendix,* Fig. S9 *D–L*) or in cells transfected with empty pcDNA3.1(+) vector (Fig. 3). Collectively, these results confirmed our prediction, based on analysis of receptor relationships, that ArBNR1 acts as a receptor for ArBN in *A. rubens*.

### ArBNP Transcripts Are Expressed in the Nervous System, Locomotory System, and Digestive System of *A. rubens*.

**Fig. 4.**
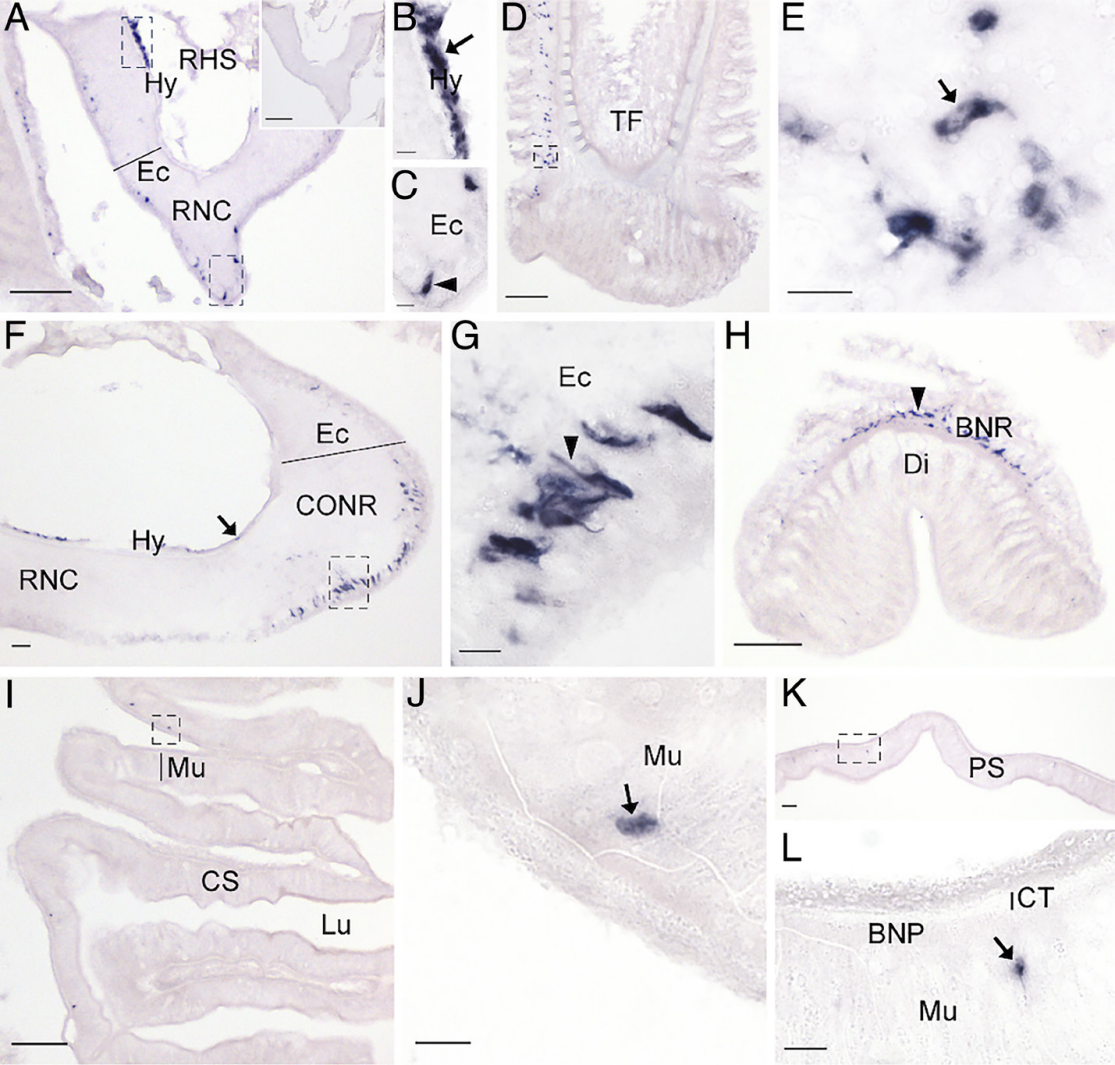
Localization of ArBN precursor expression in *A. rubens* using mRNA in situ hybridization. (*A*) Transverse section of a radial nerve cord incubated with antisense probes revealing stained cells in the hyponeural region and the ectoneural region. The *Inset* shows absence of staining in an adjacent section of the radial nerve cord incubated with sense probes, demonstrating the specificity of staining observed with antisense probes. (*B*) Higher magnification image of the upper boxed region in panel *A*, showing stained cells in the hyponeural region (arrow) of the radial nerve cord. (*C*) Higher magnification image of the lower boxed region in panel *A*, showing stained cells in the ectoneural region (arrowhead) of the radial nerve cord. (*D*) Longitudinal section of a tube foot revealing stained cells concentrated at one side of the subepithelial layer along the length of the stem and in the basal nerve ring of the disk region. (*E*) Higher magnification image of the boxed region in panel *D*, showing stained cells (arrow) at the junction between subepithelial layer of the stem and the basal nerve ring of the disk region. (*F*) Transverse section of a circumoral nerve ring showing stained cells in the hyponeural region (arrow) and the ectoneural region. (*G*) Higher magnification image of the boxed region in panel *F*, showing stained cells in the ectoneural region (arrowhead) of the circumoral nerve ring. (*H*) Longitudinal section of the disk region of a tube foot revealing abundant stained cells in the basal nerve ring (arrowhead). (*I*) Transverse section of the central disk region of the starfish body revealing sparsely distributed stained cells in the cardiac stomach. (*J*) Higher magnification image of the boxed region in panel *I* showing a stained cell (arrow). (*K*) Transverse section of the central disk region revealing stained cells in the pyloric stomach. (*L*) Higher magnification image of the boxed region in panel *K* showing a stained cell (arrow). Abbreviations: BNP, basi-epithelial nerve plexus; BNR, basal nerve ring; CONR, circumoral nerve ring; CS, cardiac stomach; CT, collagenous tissue; Di, tube foot disk; Ec, ectoneural region; Hy, hyponeural region; Lu, lumen; Mu, mucosa; PS, pyloric stomach; RHS, radial hemal strand; RNC, radial nerve cord; TF, tube foot. (Scale bars, 100 μm in *A*, *D*, *F*, *H*, *I*, and *K*, and *Inset* in *A*; 10 μm in *B*, *C*, *E*, *G*, *J*, and *L*.)

Having identified a BN-type signaling system in *A. rubens* comprising ArBN and its cognate receptor ArBNR1, our next objective was to examine the expression of the ArBN precursor (ArBNP) in *A. rubens* as a basis for investigation of the physiological roles of ArBN in this species. To accomplish this, we first investigated the distribution of ArBNP transcripts in *A. rubens* using mRNA in situ hybridization (Fig. 4). This revealed that ArBNP is expressed in the nervous system, locomotory system, and digestive system of *A. rubens* (refer to *SI Appendix,* Fig. S10 for a diagram of starfish anatomy). Absence of stained cells in adjacent sections incubated with ArBNP sense probes (Fig. 4 *A*, *Inset*) confirmed the specificity of staining observed with ArBNP antisense probes. The central nervous system of starfish comprises radial nerve cords that extend along the oral side of each of the five arms and a circumoral nerve ring in the central disk. Both the radial nerve cords and circumoral nerve ring contain ectoneural and hyponeural regions (38). ArBNP-expressing cells were observed abundantly in the hyponeural region of the radial nerve cords, but sparsely in the ectoneural region (Fig. 4*A*). In contrast, in the circumoral nerve ring ArBNP-expressing cells were mainly observed in the ectoneural region, but with some also detected in the hyponeural region (Fig. 4*F*). On either side of the radial nerve cords are two rows of tube feet (locomotory system). Expression of ArBNP was detected in the tube feet, with stained cells most abundantly observed on one side of longitudinally sectioned tube feet in the subepithelial layer along the length of the stem and in the basal nerve ring (Fig. 4*D*). In addition, prominently stained cells are present in the disk region of tube feet (Fig. 4*H*). In the digestive system, ArBNP-expressing cells were observed in the cardiac stomach (Fig. 4*I*) and pyloric stomach (Fig. 4*K*).

### Immunohistochemical Localization of ArBNP in *A. rubens*.

We attempted to generate antisera to ArBN and/or ArBNP and thereby enable visualization of their expression using immunohistochemical methods. We first attempted to generate antisera to ArBN using the antigen peptide KNYNRVFGPTY-NH_2_, which contains the amidated C-terminal decapeptide of ArBN. However, analysis of antisera using ELISA indicated low antibody titers (*SI Appendix,* Fig. S11*A*) and, accordingly, when antisera were tested on sections of arms from *A. rubens*, no specific immunostaining was observed. Therefore, informed by our previously reported success in visualizing neuropeptide expression in *A. rubens* using affinity-purified polyclonal antibodies to neuropeptide precursor proteins (39), we attempted to generate an antiserum to a peptide corresponding to the C-terminal region of ArBNP (KLAMTRMNSEAENE). Using ELISA, antibodies to the ArBNP antigen were detected in antiserum (*SI Appendix*, Fig. S11*B*). Furthermore, to facilitate enhanced immunohistochemical visualization of ArBNP expression in *A. rubens*, we affinity-purified polyclonal antibodies to the ArBNP antigen peptide and used these for a system-wide analysis of ArBNP expression.

**Fig. 5.**
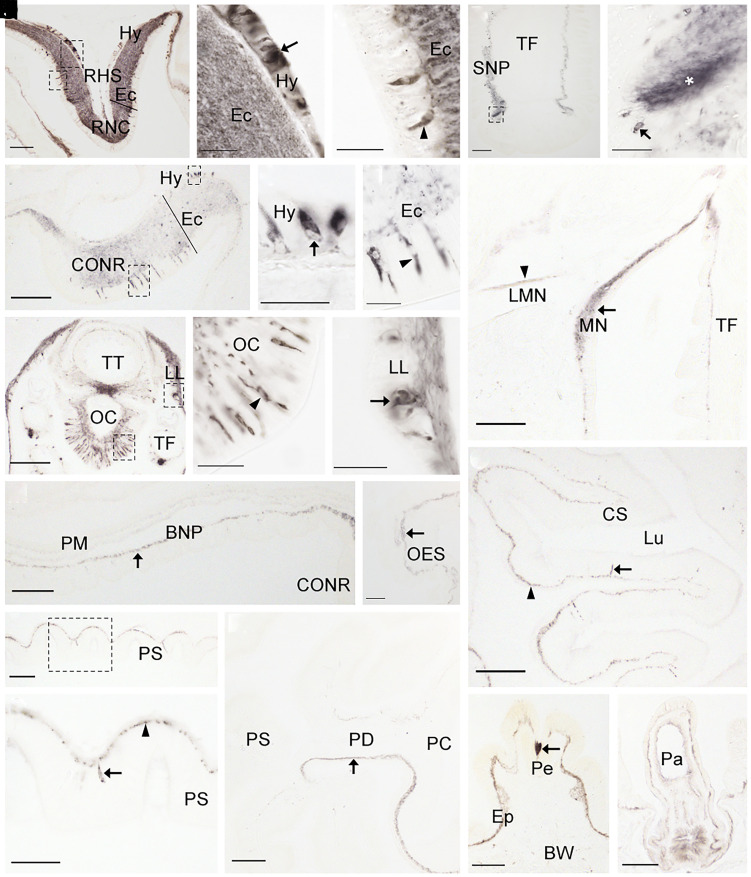
Localization of ArBN precursor expression in *A. rubens* using immunohistochemistry. (*A*) Transverse section of a radial nerve cord showing ArBNP-immunoreactivity (ir) in the hyponeural and ectoneural regions. (*B*) Higher magnification image of the upper boxed region in panel *A* showing stained cells in the hyponeural region (arrow) and stained fibers in the neuropile of the ectoneural region. (*C*) Higher magnification image of the lower boxed region in panel *A* showing stained cells (arrowhead) in the epithelial layer of the ectoneural region. (*D*) Longitudinal section of a tube foot showing ArBNP-ir in the subepithelial nerve plexus along the length of the stem and in the basal nerve ring of the disk region. (*E*) Higher magnification image of the boxed region in panel *D*, showing stained cells (arrow) and stained fibers (asterisk) associated with the basal nerve ring of the disk region. (*F*) Transverse section of the circumoral nerve ring showing ArBNP-ir in the hyponeural and ectoneural regions. (*G*) Higher magnification image of the upper boxed region of panel *F*, showing stained cells (arrow) in the hyponeural region. (*H*) Higher magnification image of the lower boxed region of panel *F*, showing stained cells (arrowhead) in the epithelium of the ectoneural region. (*I*) ArBNP-ir in marginal nerve (arrow) and lateral motor nerve (arrowhead). (*J*) Transverse section of an arm tip, showing ArBNP-ir in the terminal tentacle, lateral lappet, and optic cushion. (*K*) High magnification image of the lower boxed region of panel *J*, showing stained cells (arrowhead) in the optic cushion. (*L*) High magnification image of the upper boxed region of panel *J*, showing stained cells (arrow) in a lateral lappet. (*M*) Transverse section of the central disk region showing ArBNP-ir in the peristomial membrane (arrow). (*N*) Transverse section of the central disk region showing ArBNP-ir in the esophagus (arrow). (*O*) Transverse section of the central disk region revealing ArBNP-ir in the cardiac stomach, including stained fibers in the basi-epithelial nerve plexus (arrowhead) and a stained cell in the mucosa (arrow). (*P*) Transverse section of the central disk region showing ArBNP-ir in the pyloric stomach. (*Q*) Higher magnification image of the boxed region of panel *P*, showing staining in the basi-epithelial nerve plexus (arrowhead) and in a cell located in the mucosa (arrow). (*R*) Longitudinal section of an arm showing ArBNP-ir in the basi-epithelial nerve plexus on the oral side of a pyloric duct (arrow). (*S*) Transverse section of the aboral body wall revealing ArBN-ir in a pedicellaria (arrow). (*T*) Transverse section of the aboral body wall showing ArBN-ir in a papula. Abbreviations: BNP, basi-epithelial nerve plexus; BW, body wall; CONR, circumoral nerve ring; CS, cardiac stomach; Ec, ectoneural region; Ep, epithelium; Hy, hyponeural region; LL, lateral lappet; LMN, lateral motor nerve; Lu, lumen; MN, marginal nerve; Mu, mucosa; OC, optic cushion; OES, esophagus; Pa, papula; PC, pyloric cecum; PD, pyloric duct; Pe, pedicellaria; PM, peristomial membrane; PS, pyloric stomach; RHS, radial hemal strand; RNC: radial nerve cord; SNP, subepithelial nerve plexus; TF, tube foot; TT, terminal tentacle. (Scale bars, 100 μm in *A*, *D*, *F*, *I*, *J*, *M*–*O*, and *R*; 20 μm in *B*, *C*, *E*, *G*, *H*, *K*, and *L*; 40 μm in *Q*; and 200 μm in *P*, *S*, and *T*.)

In the radial nerve cords, ArBNP-immunoreactive (ArBNP-ir) cells were revealed in both the ectoneural and hyponeural regions (Fig. 5*A*) and in the ectoneural region dense networks of immunostained fibers were observed throughout the ectoneural neuropile (Fig. 5*A*). In the circumoral nerve ring, a prominent cluster of immunostained cells were observed in the ectoneural region, while fewer immunostained cells were observed in the hyponeural region (Fig. 5*F*). Immunostained cells and/or processes were also revealed in the segmental lateral branches of the radial nerve cords and in the marginal nerve cords (Fig. 5*I*), which are located lateral to the outer row of tube feet on each side of the arm, in parallel with the radial nerve cords. In the tube feet, immunostaining of ArBNP was observed in the subepithelial nerve plexus along the length of the podium and extending into the basal nerve ring of the disk region, with one side revealing more extensive staining than the other side (Fig. 5*D*). At the tip of each arm is a sensory organ known as the terminal tentacle (40, 41). ArBNP-ir cells and an immunoreactive nerve plexus were observed in the terminal tentacle and associated sensory organs including the optic cushion and lateral lappets (Fig. 5*J*). In the body wall, immunostaining was observed in several appendages including pedicellariae (Fig. 5*S*), which are defensive organs, and papulae (Fig. 5*T*), which enable gas exchange between external seawater and the coelomic fluid (42). ArBNP-ir cells and/or fibers were observed in the mucosa and basi-epithelial nerve plexus in several regions of the digestive system, including the peristomial membrane (Fig. 5*M*), esophagus (Fig. 5*N*), cardiac stomach (Fig. 5*O*), pyloric stomach (Fig. 5*P*), and pyloric ducts (Fig. 5*R*).

### ArBN Causes Dose-Dependent Contraction of Cardiac Stomach and Tube Foot Preparations from *A. rubens*.

**Fig. 6.**
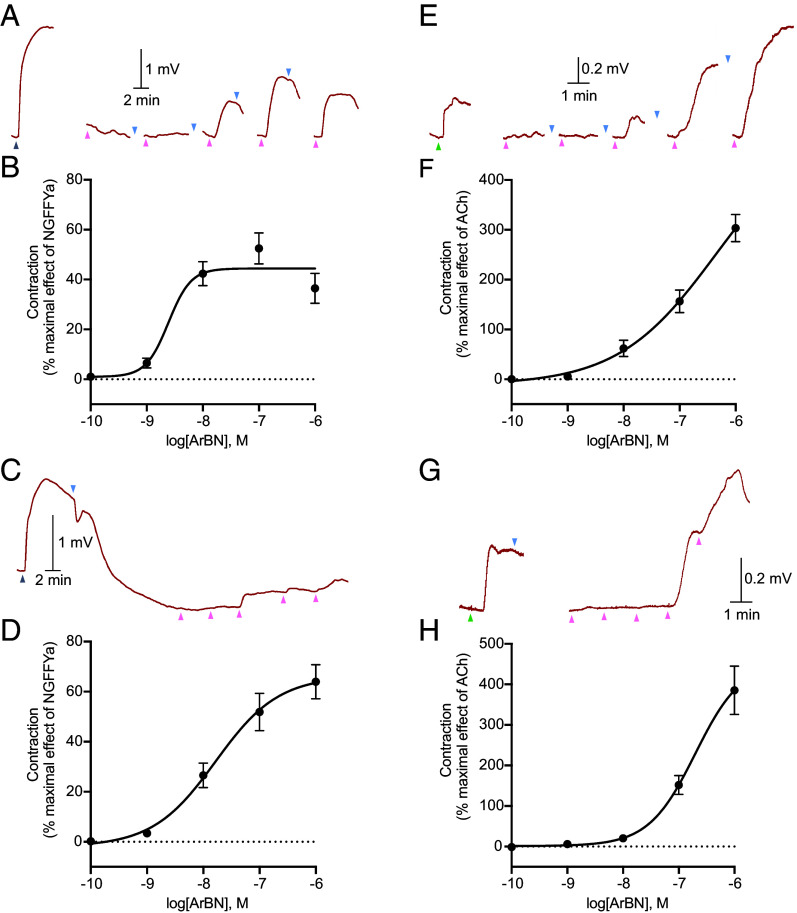
ArBN causes dose-dependent contraction of cardiac stomach and tube foot preparations from *A. rubens*. (*A*) Representative recording showing the effects of NGFFYamide (10^−7^ M) and ArBN (10^−10^ to 10^−6^ M) on a cardiac stomach preparation, with washing between each application of the test agent. (*B*) Graph showing that ArBN causes dose-dependent contraction of cardiac stomach preparations (n = 10) at concentrations ranging from 10^−9^ M to 10^−6^ M, using the method shown in *A*. (*C*) Representative recording showing the effects of NGFFYamide (10^−7^ M) and ArBN (10^−10^ to 10^−6^ M) on a cardiac stomach preparation, without washing between each application of the test agent. (*D*) Graph showing the cumulative contracting effect of ArBN (10^−10^ to 10^−6^ M) on cardiac stomach preparations (n = 8), using the method shown in *C*. The graphs in *B* and *D* show mean percentage (±SEM) of the contraction induced by 10^−7^ M NGFFYamide. (*E*) Representative recording showing the effects of acetylcholine (ACh, 10^−5^ M) and ArBN (10^−10^ to 10^−6^ M) on a tube foot preparation, with washing between each application of the test agent. (*F*) Graph showing that ArBN causes dose-dependent contraction of tube foot preparations (n = 10) at concentrations ranging from 10^−9^ M to 10^−6^ M, using the method shown in *E*. (*G*) Representative recording showing the effect of ACh (10^−5^ M) and ArBN (10^−10^ to 10^−6^ M) on a tube foot preparation, without washing between each application of the test agent. (*H*) Graph showing the cumulative contracting effect of ArBN (10^−10^ to 10^−6^ M) on tube foot preparations (n = 7), using the method shown in *G*. The graphs in *F* and *H* show mean percentage (±SEM) of the contraction induced by 10^−5^ M ACh. In *A*, *C*, *E*, and *G*, the gray upward pointing arrowheads show when 10^−7^ M NGFFYamide was added, the green upward pointing arrowheads show when 10^−5^ M ACh was added, the pink upward pointing arrowheads show when ArBN was added (left to right, from 10^−10^ to 10^−6^ M) and the blue downward pointing arrowheads show when the preparation was washed with seawater. The raw data for the graphs shown in this figure are available in Dataset S2.

Based on the expression of ArBNP in the cardiac stomach and tube feet of *A. rubens*, experiments were performed to investigate effects of ArBN on in vitro preparations of these organs. ArBN caused dose-dependent contraction of cardiac stomach and tube foot preparations when tested at concentrations from 10^−9^ to 10^−6^ M. The effects of ArBN on cardiac stomach preparations were compared with the effects of NGFFYamide, which causes contraction of cardiac stomach preparations in vitro (43). In experiments where cardiac stomach preparations were washed in between application of ArBN at different concentrations, ArBN caused 36.48 ± 6.02% (n = 10) of the contraction induced by 10^−7^ M NGFFYamide (defined as 100% contraction) when at the highest concentration (10^−6^ M) tested (Fig. 6*B*). Maximal efficacy was observed at 10^−7^ M ArBN, which caused 52.48 ± 6.19% (n = 10) of the contraction induced by 10^−7^ M NGFFYamide (Fig. 6*B*). In experiments where preparations were not washed in between application of ArBN, following application of ArBN at 10^−10^ M to 10^−6^ M the cumulative contracting effect of ArBN was 63.98 ± 6.80% (n = 8) of the contraction induced by 10^−7^ M NGFFYamide (defined as 100% contraction) (Fig. 6*D*). Thus, ArBN causes dose-dependent contraction of cardiac stomach preparations from *A. rubens*, but is less effective than NGFFYamide. The effects of ArBN on tube foot preparations were compared with the effects of acetylcholine (ACh), which causes contraction of tube foot preparations in vitro (44). In experiments where tube foot preparations were washed in between application of ArBN at different concentrations, ArBN had a maximal effect of 303.50 ± 27.16% (n = 10) of the effect of 10^−5^ M ACh (defined as 100% contraction) when at the highest concentration (10^−6^ M) tested (Fig. 6*F*). In experiments where preparations were not washed in between application of ArBN, following application of ArBN at 10^−10^ M to 10^−6^ M the cumulative contracting effect of ArBN was 385.42 ± 59.70% (n = 7) of the contraction induced by 10^−5^ M of ACh (defined as 100% contraction) (Fig. 6*H*). Thus, ArBN causes dose-dependent contraction of tube foot preparations from *A. rubens* and, furthermore, it is more effective than ACh in causing contraction of tube feet.

We also tested ArBN_5-14_ (NYNRVFGPTY-NH_2_) and the putative ArBNP-derived peptide ArBNP_65-81_ (EPMPSSLALYIANLSP-NH_2_) on cardiac stomach and tube foot preparations that had exhibited responses to ArBN. However, neither ArBN_5-14_ nor ArBNP_65-81_ had any observable effect (*SI Appendix,* Fig. S12).

### ArBN Causes Cardiac Stomach Retraction In Vivo.

**Fig. 7.**
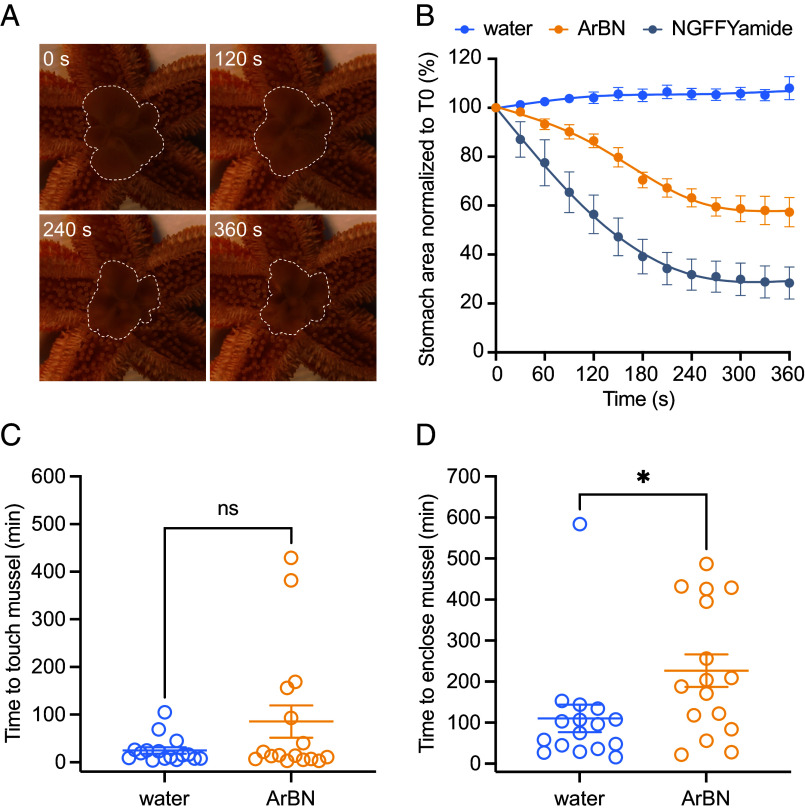
ArBN causes cardiac stomach retraction and inhibits feeding behavior in *A. rubens*. (*A* and *B*) ArBN causes cardiac stomach retraction in *A. rubens*. (*A*) Photographs from an experiment showing that the two-dimensional area (marked by white dashed lines) of a cardiac stomach is progressively reduced at 120, 240, and 360 s after injection (0 s) of 10 μL of ArBN (10^−4^ M). (*B*) Graph comparing the everted cardiac stomach area in starfish injected with water (blue; 10 µL; n = 7), ArBN (yellow; 10 µL of 10^−4^ M; n = 7), and NGFFYamide (gray; 10 µL of 10^−4^ M; n = 7). The two-dimensional area of cardiac stomach everted at each time point was normalized to the area of cardiac stomach everted just before injection. Each point represents means (±SEM) from seven separate experiments. (*C* and *D*) ArBN inhibits feeding behavior in *A. rubens*. (*C*) For starfish that successfully fed, injection of ArBN (10 μL of 10^−4^ M) had no effect on the time taken for starfish to first touch a mussel. (*D*) For starfish that successfully fed, the time taken to enclose a mussel was significantly longer (*P* = 0.0324) in the group that was injected with 10 μL of 10^−4^ M ArBN (yellow, n = 16) than in the group injected with 10 μL of water (blue, n = 16). Data were analyzed statistically using a two-tailed Student’s *t*-test in Prism 10 and are shown as scatter plots. The raw data for the graphs shown in this figure are available in Datasets S3 and S4.

Starfish (*A. rubens*) feed by everting their cardiac stomach out of the mouth over the digestible soft tissues of prey (e.g., mussels) and when feeding is completed, the stomach is retracted back into the central disk region of the body (45). Previous studies have revealed that NGFFYamide triggers cardiac stomach contraction in vitro and retraction in vivo in *A. rubens* (43). As ArBN caused contraction of *A. rubens* cardiac stomach preparations in vitro (Fig. 6 *A*-*D*), it was of interest to investigate whether ArBN can also trigger cardiac stomach retraction in vivo. Cardiac stomach eversion was induced by immersion of specimens in 2% MgCl_2_ dissolved in seawater (43). Control experiments were performed first and, consistent with findings from a previous study (43), injection of distilled water (negative control) had no effect (*SI Appendix,* Fig. S13), while injection of NGFFYamide (10 µL of 10^−4^ M; positive control) caused cardiac stomach retraction (*SI Appendix,* Fig. S13). When 10 µL of 10^−4^ M ArBN was injected, cardiac stomach retraction was also observed (Fig. 7*A*) and the mean percentage two-dimensional area of the everted cardiac stomach 6 min after injection was 57.30 ± 6.00% of the area before injection (Fig. 7*B*). Thus, ArBN induces cardiac stomach retraction in vivo, but is less effective than NGFFYamide, consistent with the relative efficacy of these neuropeptides in inducing cardiac stomach contraction in vitro (Fig. 6 *A*–*D*).

### ArBN Has an Inhibitory Effect on Feeding Behavior in *A. rubens*.

Previous studies have revealed that some neuropeptides that cause cardiac stomach retraction in vivo also have an inhibitory effect on feeding behavior in *A. rubens* (46, 47). Therefore, because ArBN triggers cardiac stomach retraction in *A. rubens*, we also investigated its effects on feeding behavior. In these experiments starfish that had been starved for 30 d were placed at one end of a tank and a mussel was placed at the opposite end. The behavior of starfish that had been injected with ArBN (10 μL of 10^−4^ M) was compared with control animals that had been injected with water (10 μL). When comparing behavior of ArBN-injected and water-injected animals there was no significant difference in the proportion of animals that approached and touched the mussel or the proportion of animals that fed on the mussel (*SI Appendix,* Fig. S14 *A* and *B*). More specifically, both in animals that did proceed to feed and in animals that approached and touched the mussel but did not proceed to feed, there was no significant difference in the time taken to touch the mussel between ArBN-injected animals and water-injected animals (Fig. 7*C* and *SI Appendix,* Fig. S14*C*). However, in the subset of animals that did feed, ArBN-injected animals took longer to enclose a mussel than water-injected animals (Fig. 7*D*). Thus, evidence that ArBN has an inhibitory effect on feeding behavior in *A. rubens* was obtained.

## Discussion

The identification of bombesin (BN) in the toad *B. bombina* heralded the beginning of research on a novel family of bioactive peptides, with the neuropeptides gastrin-releasing peptide (GRP) and neuromedin B (NMB) subsequently being identified as representatives of this family in mammals (12, 13, 16, 18). Then, discovery of receptors for GRP and/or NMB yielded insights into the mechanisms by which BN-type neuropeptides exert their effects (20–23). In addition, identification of these receptors facilitated investigation of the phylogenetic distribution of BN-type signaling, revealing its occurrence throughout the vertebrates (4). Furthermore, discovery of a BN-type peptide and three BN-type receptors in the cephalochordate *B. floridae* revealed that the evolutionary origin of BN-type signaling can be traced back further to the common ancestor of chordates (24). Here, we report the discovery and functional characterization of a BN-type signaling system in a nonchordate deuterostome—the starfish *A. rubens*.

### Discovery of a BN-Type Neuropeptide Signaling System in an Echinoderm.

Putative precursors of BN-type peptides in echinoderms were identified by analysis of transcriptome/genome sequence data and analysis of the predicted peptide sequences revealed similarity with BN-type peptides in chordates. Further evidence that the echinoderm precursors of BN-like peptides are orthologs of chordate BN-type peptides was obtained from phylogenetic analysis of these proteins and comparative analysis of the exon/intron structure of genes encoding these proteins. Phylogenetic analysis revealed that the echinoderm precursor proteins are positioned in a clade together with chordate BN-type precursors that is distinct from clades containing precursors of closely related peptides (chordate ET-type peptides and protostome CCHa/EP-type neuropeptides). Furthermore, genes encoding putative BN-type precursors in echinoderms and BN-type precursors in chordates have in common a phase 1 intron that interrupts the coding sequence for the predicted neuropeptide sequence and a phase 0 intron that interrupts the coding sequence for the C-terminal region of the precursor proteins. Collectively, these findings provide compelling evidence that the putative BN-type precursors identified in echinoderms are indeed orthologs of BN-type precursors in chordates.

Our next objective was to identify the receptor(s) that mediate effects of the BN-type neuropeptides in echinoderms. To address this issue, we selected the starfish *A. rubens* as an experimental system and first used mass spectrometry (MS) to determine the mature structure(s) of the neuropeptide(s) derived from the BN-type precursor in this species. A C-terminally amidated peptide with the structure EPRRNYNRVFGPTY-NH_2_ was identified in an extract of *A. rubens* radial nerve cords and we refer to this peptide as ArBN. However, an N-terminally truncated form of ArBN was also detected in the extract (NYNRVFGPTY-NH_2_) and we refer to this peptide as ArBN_5-14_. Then candidate receptors for ArBN and/or ArBN_5-14_ were identified in *A. rubens* by analysis of transcriptome/genome sequence data for the presence of transcripts/genes encoding proteins closely related to chordate BN-type receptors. Eleven BN receptor-related proteins were identified (ArBNR1-11) but phylogenetic analysis indicated that only one of these (ArBNR1) is an ortholog of chordate BN-type receptors. Accordingly, cell-based assays in which CHO-K1 cells were transfected with ArBNR1 and the chimeric G protein Gqs5 revealed that ArBN is a potent ligand for ArBNR1 (EC_50_ = 1.69 × 10^−11^ M). In contrast, only very high concentrations of ArBN_5-14_ (10^−5^ M) triggered responses in cells expressing ArBNR1. Therefore, we conclude that ArBN, but not ArBN_5-14_, acts as a ligand for ArBNR1 physiologically in *A. rubens*. The evolutionary and functional significance of the expanded family of ArBNR1-related proteins in *A. rubens* (ArBNR2-11) and other echinoderms remains to be determined. ArBN did not act as a ligand when tested on two of these proteins (ArBNR2, ArBNR3) and therefore this family of ArBNR1-related proteins may have evolved as receptors for other types of ligands.

Precursors of ArBN-type peptides were also identified in other echinoderms, including the starfish *A.* cf. *solaris*, the sea urchin *S. purpuratus* and the sea cucumber *A. japonicus*, and the extensive sequence similarity that these peptides share with ArBN is shown in Fig. 1 and *SI Appendix,* Fig. S1. Accordingly, orthologs of ArBNR1 were identified in *A.* cf. *solaris*, *S. purpuratus,* and *A. japonicus* and therefore, based on our experimental findings from *A. rubens*, we predict that the BN-type peptides identified in these species (Fig. 1) act as ligands for the BNR1-type proteins in these species (Fig. 3). In conclusion, the experimental identification of ArBNR1 as the receptor for ArBN in *A. rubens* and the occurrence of orthologs in other echinoderms are important as discoveries of BN-type signaling in nonchordates. Furthermore, these findings indicate that the evolutionary history of BN-type signaling extends back to the common ancestor of the deuterostomes.

### Functional Characterization of the BN-Type Neuropeptide ArBN in the Starfish *A. rubens*.

Having identified ArBN and its receptor (ArBNR1) in *A. rubens*, our next objective was to gain insights into the physiological roles of ArBN. Expression of the ArBN precursor (ArBNP) was analyzed using mRNA in situ hybridization and immunohistochemistry and, importantly, there was consistency in the anatomical distribution of stained cells revealed by these two techniques, providing evidence of the specificity of the affinity-purified polyclonal antibodies to ArBNP that we generated. Consistent with our analysis of the expression of many other neuropeptides (10, 44, 47, 48), ArBNP has a widespread pattern of expression in *A. rubens*. Thus, ArBNP-expressing cells and processes are present in the central nervous system, which comprises the radial nerve cords that extend along the oral side of each arm and the circumoral nerve ring that links the radial nerve cords in the central disk region. More specifically, the radial nerve cords and circumoral nerve ring comprise two regions: the ectoneural region, which is thought to largely comprise sensory neurons and interneurons, and the hyponeural region, which contains the cell bodies of somatic motoneurons (40, 49). ArBNP expression was revealed in subpopulations of both ectoneural and hyponeural neurons. Consistent with ArBNP expression in the hyponeural region of the radial nerve cords, immunostaining was also observed in the lateral motor nerve, which contains the axons of hyponeural motoneurons (40, 44). Therefore, we infer that ArBN may be involved in mediating motoneuronal control of peripheral organs/muscles. Furthermore, the presence of ArBNP-expressing cells and fibers in the ectoneural region of the radial nerve cords and the circumoral nerve ring suggests that ArBN is involved in coordination of whole-animal behavior in response to sensory input.

ArBNP expression was also revealed in tube feet, which enable locomotory and feeding behavior in starfish. Accordingly, in vitro experiments revealed that ArBN causes dose-dependent contraction of tube foot preparations from *A. rubens*. In this regard, ArBN joins a growing list of neuropeptides that have been found to have excitatory effects on tube foot preparations from *A. rubens* (10, 46, 47). Therefore, we infer that ArBN is one of several neuropeptides that may mediate neural control of tube foot contractility during locomotory and/or feeding behavior. A noteworthy feature of ArBNP expression in tube feet is a population of ArBNP-expressing cells concentrated in the disk region of these organs, which contains an adhesive epidermis comprising secretory cells that release adhesive or deadhesive substances (50) and allow tube feet to bind to and disengage from substrata during locomotion or feeding. Therefore, the expression of ArBNP in the disk region of tube feet may indicate that ArBN is also involved in regulation of the secretion of adhesive or deadhesive substances.

ArBNP expression was revealed in many regions of the digestive system in *A. rubens*. Accordingly, in vitro experiments revealed that ArBN causes dose-dependent contraction of cardiac stomach preparations from *A. rubens*, consistent with the excitatory effects of ArBN on tube foot preparations. Discovery of neuropeptides that exert excitatory or inhibitory effects on the cardiac stomach in vitro is of interest with respect to the unusual feeding behavior of *A. rubens* and other starfish. When starfish feed on bivalve mollusks such as mussels, they use the collective pulling power of tube feet located on the underside of their arms to pry apart the two shells. They then evert their cardiac stomach out of their mouth into the shell cavity and over the soft tissue of prey, and enzymes secreted by the stomach and/or other regions of digestive system proceed to digest the prey tissues. After this, the digested tissue is transported to the pyloric ceca located in each arm and the cardiac stomach is retracted back into the central disk region (45). Previous studies have revealed that some neuropeptides, including NGFFYamide and the sulfakinin/cholecystokinin-type neuropeptides ArSK/CCK1 and ArSK/CCK2, that cause cardiac stomach contraction in vitro also trigger retraction of the everted cardiac stomach when injected in vivo (43, 46, 47). Therefore, here we also tested the in vivo effects of ArBN in *A. rubens* and observed that, consistent with its contracting effect on the cardiac stomach in vitro, ArBN triggers retraction of the everted cardiac stomach. Furthermore, consistent with the relative efficacy of ArBN and NGFFYamide in vitro, ArBN was less effective than NGFFYamide in triggering cardiac stomach retraction.

Based on the effects of NGFFYamide, ArSK/CCK1, and ArSK/CCK2 in triggering a process associated with termination of feeding behavior—cardiac stomach retraction—the effects of neuropeptides on feeding behavior of starfish have also been investigated. In these experiments starfish were starved and then presented with prey (a mussel) in a testing tank and the time taken to touch, enclose, and then consume the mussel was measured. Injection of NGFFYamide was found to cause an increase in the time taken for starfish to touch and enclose a mussel, by comparison with control animals injected with water (46). Injection of ArSK/CCK1 or ArSK/CCK2 did not affect the time taken to touch a mussel but both peptides caused an increase in the time taken to enclose a mussel and a reduction in the number of animals that successfully consumed a mussel (47). Collectively, these findings indicated that both NGFFYamide and SK/CCK-type peptides act as inhibitory regulators of feeding behavior in *A. rubens* (46, 47). Accordingly, here we also investigated the effect of injection of ArBN on feeding behavior in *A. rubens* and observed that ArBN caused a significant increase in the time taken for animals to enclose a mussel. The mechanisms by which NGFFYamide, SK/CCK-type peptides, and ArBN exert inhibitory effects on feeding behavior remain to be determined. However, based on the expression pattern and effects of ArBN in *A. rubens*, several potential mechanisms of action can be postulated. Thus, the effects could be a manifestation of the contracting action of ArBN on both tube feet and the cardiac stomach, thereby impeding enclosure and opening of a gap between the two valves of their prey and/or impeding eversion of the cardiac stomach. Alternatively or additionally, the inhibitory effect of ArBN on feeding behavior could also be a manifestation of effects of this peptide on the central nervous system that then affects coordination of whole-animal behavior.

### The Evolution and Comparative Physiology of BN-Type Neuropeptide Signaling.

The discovery of ArBN and its receptor (ArBNR1) in the starfish *A. rubens* provides an important insight into the evolution of BN-type neuropeptide signaling. Hitherto BN-type signaling has only been characterized in chordates and therefore our discovery of a BN-type neuropeptide signaling system in an echinoderm has revealed that the evolutionary history of BN-type signaling extends to the common ancestor of deuterostomes. Accordingly, orthologs of the *A. rubens* BN-type precursor (ArBNP) and ArBNR1 were also identified in other echinoderms, including the starfish *A.* cf. *solaris*, the sea urchin *S. purpuratus* and the sea cucumber *A. japonicus*. Consistent with the occurrence of BN-type signaling in echinoderms, here we also identified two BN-type receptors (SkowBNR1, SkowBNR2) in the acorn worm *S. kowalevskii*—a species belonging to the phylum Hemichordata that is a sister phylum to the Echinodermata in the Ambulacraria clade of the deuterostomes. However, the precursor(s) of peptide(s) that act as ligands for these receptors remain to be identified.

The functional characterization of ArBN in *A. rubens* provides insights into the comparative physiology of BN-type neuropeptide signaling. Previous experimental studies have revealed that the BN-type neuropeptides GRP and NMB act as myoexcitatory agents in vertebrates. For example, GRP and NMB cause dose-dependent contraction of the fundic muscle of mice (51) and BN has an excitatory effect on intestinal smooth muscle from caimans (52). Accordingly, as discussed above, here we found that ArBN has a potent myoexcitatory effect on both tube foot and cardiac stomach preparations from *A. rubens*. Furthermore, it is noteworthy that the efficacy of ArBN in causing contraction of tube foot preparations was approximately three-four times greater than that of acetylcholine. The myoexcitatory action of BN-type neuropeptides in vertebrates and in starfish indicates that this is an evolutionarily ancient physiological role that may have originated in a common ancestor of the deuterostomes. Therefore, in future studies, it will be of interest to investigate whether this action of BN-type neuropeptides is also observed in other echinoderms.

BN-type neuropeptides have a variety of other physiological roles in mammals/vertebrates, including stimulation of the release of gastrointestinal hormones, suppression of food intake and mediation of satiety (30, 32, 33). Therefore, it is interesting that in the starfish *A. rubens* ArBN causes cardiac stomach contraction in vitro, retraction of the everted cardiac stomach in vivo and a significant increase in the time taken for animals to enclose prey (a mussel). These findings indicate that ArBN has a physiological role in inhibiting the onset of feeding behavior and/or promoting the termination of feeding behavior in *A. rubens*. Furthermore, these findings also indicate that inhibitory regulation of feeding behavior is an evolutionarily ancient role of BN-type neuropeptides that may have originated in a common ancestor of the deuterostomes. Facilitated by our discovery and functional characterization of BN-type signaling in the starfish *A. rubens*, broader insights into the comparative physiology of BN-type neuropeptides could now be obtained from experimental studies on other echinoderms (e.g., sea cucumbers, sea urchins) and hemichordates.

## Materials and Methods

### Animals.

Adult starfish (*A. rubens*) were collected and maintained, as described previously (53).

### Identification of Putative BN-Type Precursors in Echinoderms.

To identify novel putative neuropeptide precursors in echinoderms, an amino acid sequence file was compiled that contained open reading frames (ORFs) from all 6-frame translations of echinoderm expressed sequence tags (ESTs) and nucleotide sequences downloaded from the NCBI database. These sequences were then screened for the presence of putative precursors of peptide hormones and neuropeptides using a Hidden Markov Model described previously (54). Restricting analysis to the top 5,000 candidates, sequences were screened for the presence of conserved families of peptides. To achieve this, lists of predicted peptides (using the HMM predictions) from distinct candidate precursor sequences that shared a proportion of identical residues at their N- or C-terminus (for instance 4 out of 6 residues) were identified. Members of one of these families of orthologous proteins contained features found in neuropeptide precursors, including an N-terminal signal peptide determined by SignalP-6.0 (https://services.healthtech.dtu.dk/services/SignalP-6.0/) and a putative neuropeptide sequence with a conserved C-terminal GPXXG motif that was bounded by dibasic residues (KR or RR) (54). Furthermore, analysis of the putative neuropeptide sequences revealed some similarities with vertebrate BN-type peptides.

Since BN-type peptides were expected to be present in echinoderms because of the occurrence of BN-type receptors in this phylum (4), the potential neuropeptides derived from members of this uncharacterized echinoderm peptide precursor family were aligned with vertebrate/cephalochordate BN-type neuropeptides (obtained from NCBI, https://www.ncbi.nlm.nih.gov/). The alignment of the peptide amino acid sequences (*SI Appendix,* Table S1) was performed using the MUSCLE method plugin in SnapGene (https://www.snapgene.com/) and then manually curated. Conserved residues that occur in at least one echinoderm species and several chordate species were identified.

### Phylogenetic Analysis of Precursors of BN-Type Peptides and Closely Related Peptides.

Previous studies have revealed that BN-type signaling is closely related to the vertebrate ET-type and the protostome CCHa/EP-type signaling systems (4). Therefore, to further investigate relationships between putative BN-type peptide precursors in echinoderms and BN/ET/CCHa/EP-type precursors in other taxa, a maximum-likelihood phylogenetic tree was generated using W-IQ-TREE (http://iqtree.cibiv.univie.ac.at/) (55) (JTT+I+G4 model, 1,000 bootstrap replicates). Elevenin-type precursors were selected as an outgroup to root the tree because previous studies have revealed that elevenin-type receptors are closely related to BN/ET/CCHa/EP-type receptors (1, 4). The precursor sequences (*SI Appendix,* Table S2) were aligned using the MAFFT online tool (https://mafft.cbrc.jp/alignment/server/) and a phylogenetic tree was generated using FigTree.v1.4.4 (http://tree.bio.ed.ac.uk/software/figtree/).

### Analysis of the Exon/Intron Structure of Genes Encoding BN-Type Precursors.

The sequences of transcripts and genes encoding precursors of the putative BN-type peptides in echinoderms and known BN-type peptides in chordates were obtained from the NCBI GenBank database (*SI Appendix,* Table S3). The online tool Splign (https://www.ncbi.nlm.nih.gov/sutils/splign/splign.cgi) (56) was used to determine gene structure and Adobe Illustrator was used to generate diagrams comparing the structure of genes from echinoderms and chordates.

### Cloning and Sequencing of a cDNA Encoding ArBNP.

Having identified a candidate precursor of a BN-type neuropeptide in *A. rubens* (ArBNP) based on analysis of transcriptome/genome sequence data, we cloned and sequenced a cDNA encoding this protein. Total RNA was extracted from radial nerve cords using the Monarch® Total RNA Miniprep Kit (NEB, Cat. No. T2010S) and reverse transcription was conducted using the QuantiTect Reverse Transcription Kit (Qiagen, Cat. No. 205311). A cDNA comprising the open reading frame of ArBNP was amplified from *A. rubens* radial nerve cord total cDNA by PCR using forward primer 5′-CCACATCACCGTGAACGACT-3′, reverse primer 5′-TCCCCGTTGTTAGGGTAAGAC-3′ and Q5 High-fidelity DNA polymerase (NEB, Cat. No. M0491S). Then the ArBNP cDNA was blunt-end cloned into pBluescript II SK+ vector (Agilent Technologies, Cat. No. 212205) that had been cut with restriction enzyme *EcoRV*-HF (NEB, Cat. No. R3195T) and sequenced (Source Bioscience, UK) using standard T7 and T3 sequencing primers.

### Structural Characterization of the ArBNP-Derived Neuropeptides Using MS.

Having identified a candidate precursor of BN-type neuropeptide(s) in *A. rubens* (ArBNP), we investigated the occurrence of peptides derived from this protein by analysis of an extract of radial nerve cords from this species using MS. The BN-type peptide predicted to be derived from ArBNP has the predicted mature structure EPRRNYNRVFGPTY-NH_2_ (ArBN). However, this peptide contains a pair of arginine residues at positions 3 and 4, which could function as a dibasic cleavage site. Therefore, the decapeptide (NYNRVFGPTY-NH_2_) could also be derived from ArBNP and we refer to this putative peptide as ArBN_5-14_. Furthermore, in the C-terminal region of ArBNP there is another peptide sequence bounded by potential monobasic/dibasic cleavage sites. The predicted mature structure of this peptide is EPMPSSLALYIANLSP-NH_2_ and we refer to it as ArBNP_65-81_. To facilitate investigation of the presence of ArBN, ArBN_5-14_, and ArBNP_65-81_ in *A. rubens* using MS, all three peptides were custom synthesized and purified (Peptide Protein Research Ltd., UK). Details of the methods employed for preparation of an *A. rubens* radial nerve cord extract and use of NanoLC-ESI-MS/MS for analysis of the extract are described in *SI Appendix*, *SI Methods*.

### Identification of Candidate BN-Type Receptors in the Starfish *A. rubens*.

To identify candidate BN-type receptors in *A. rubens*, BLAST analysis of *A. rubens* neural transcriptome (8) and genome sequence data (https://www.ncbi.nlm.nih.gov/datasets/genome/GCF_902459465.1/) was performed. The methods employed for this analysis are described in *SI Appendix*, *SI Methods*.

### Functional Characterization of Putative *A. rubens* BN-Type Receptors.

Having investigated relationships of eleven *A. rubens* BN-type receptor candidates (ArBNR1-11) with BN-type, ET-type, and CCHa/EP-type receptors in other taxa, ArBNR1 was identified as the best candidate and therefore this was selected for experimental testing. However, two other receptors (ArBNR2 and ArBNR3) were also tested. The methods employed for functional characterization of *A. rubens* neuropeptide receptors have been described previously (57) and a description of the methods employed in this study is included in *SI Appendix*, *SI Methods*.

### Mapping the Expression of ArBNP Transcripts in *A. rubens* Using mRNA In Situ Hybridization.

A pBluescript II SK+ plasmid containing the cloned ArBNP cDNA was linearized by PCR with standard M13 primers (Forward primer: 5′-GTAAAACGACGGCCAGTG-3′; Reverse primer: 5′-GGAAACAGCTATGACCATG-3′) and Q5 High-fidelity DNA polymerase (NEB, Cat. No. M0491S). Then PCR products were purified using the QIAquick gel extraction kit (Qiagen, Cat. No. 28704). The methods employed for probe synthesis and analysis of neuropeptide precursor expression in *A. rubens* using mRNA in situ hybridization have been described previously (58) and a description of the methods employed in this study is included in *SI Appendix*, *SI Methods*.

### Generation and Characterization of Antisera to ArBN and ArBNP and Immunohistochemical Localization of ArBNP in *A. rubens*.

To facilitate localization of ArBN expression in *A. rubens* using immunohistochemistry, antisera to ArBN were generated, as described in *SI Appendix*, *SI Methods*. However, because generation of antisera to ArBN was unsuccessful (*SI Appendix,* Fig. S11*A*), an alternative experimental approach for neuropeptide immunohistochemistry was used by generating antisera to a peptide corresponding to the C-terminal region of ArBNP. This experimental approach has been used successfully for other neuropeptides in *A. rubens* (39) and details of the methods employed here are described in *SI Appendix*, *SI Methods*. The methods employed for immunohistochemical localization of neuropeptide expression in *A. rubens* have been described previously (44) and a description of the methods employed in this study is included in *SI Appendix*, *SI Methods*.

### Analysis of In Vitro Effects of ArBN, ArBN_5-14_, and ArBNP_65-81_ on Cardiac Stomach and Tube Foot Preparations from *A. rubens*.

ArBN (EPRRNYNRVFGPTY-NH_2_), ArBN_5-14_ (NYNRVFGPTY-NH_2_), and ArBNP_65-81_ (EPMPSSLALYIANLSP-NH_2_), were tested for effects on in vitro cardiac stomach and tube foot preparations from *A. rubens*. The preparations were dissected as described previously (43, 59–61), with the organs immersed in ice-cold artificial seawater during dissection. A description of the methods employed for investigation of in vitro effects of ArBN on cardiac stomach and tube foot preparations from *A. rubens* is included in *SI Appendix*, *SI Methods*.

### Analysis of the In Vivo Effect of ArBN on the Everted Cardiac Stomach of *A. rubens*.

The neuropeptide NGFFYamide has been shown to induce cardiac stomach contraction in vitro and cause cardiac stomach retraction in vivo (43). Here, ArBN was found to induce contraction of cardiac stomach preparations in vitro. Therefore, we performed experiments to investigate whether ArBN causes cardiac stomach retraction in *A. rubens* when injected in vivo. A description of the methods employed for investigation of in vivo effects of ArBN on the everted cardiac stomach of *A. rubens* is included in *SI Appendix*, *SI Methods*.

### Analysis of the In Vivo Effect of ArBN on Feeding Behavior of *A. rubens*.

The neuropeptides NGFFYamide, ArSK/CCK1, and ArSK/CCK2 inhibit the onset of feeding behavior of *A. rubens* (46, 47). Here, experiments were performed to investigate whether ArBN affects feeding behavior in *A. rubens* using similar methods to those employed previously (46, 47). A description of the methods employed for investigation of in vivo effects of ArBN on feeding behavior in *A. rubens* is included in *SI Appendix*, *SI Methods*.

## Supplementary Material

Appendix 01 (PDF)

Dataset S01 (XLSX)

Dataset S02 (XLSX)

Dataset S03 (XLSX)

Dataset S04 (XLSX)

## Data Availability

All study data are included in the article and/or supporting information.
