# Supplementary material for: Discovery and functional characterization of a bombesin-type neuropeptide signaling system in an invertebrate
Source: Proc Natl Acad Sci U S A. 2025 Mar 28;122(13):e2420966122. doi: 10.1073/pnas.2420966122 (PMC12002301; doi:10.1073/pnas.2420966122)
Supplement: Supplementary file 1 — Appendix 01 (PDF) [file pnas.2420966122.sapp.pdf]

PNAS

Supporting Information for

Discovery and functional characterization of a bombesin-type neuropeptide signaling system in an invertebrate

Weiling Huang[1], Xingxing Zhong[1], Cleidiane G. Zampronio[2], Andrew R. Bottrill[2], Kite G.E. Jones[1], Ana B. Tinoco[1+], Lijin Guo[3], Michaela Egertová[1], Olivier Mirabeau[4], Maurice R. Elphick[1*]

1. Centre for Evolutionary and Functional Genomics, School of Biological and Behavioural Sciences, Queen Mary University of London, London, E1 4NS, UK.

2. School of Life Sciences, University of Warwick, Coventry, CV4 7AL, UK

3. College of Animal Science, South China Agricultural University, Guangzhou, China.

4. Institut Pasteur, Université Paris Cité, Bioinformatics and Biostatistics Hub, F-75015 Paris, France.

+ Present address: Department of Biology, Faculty of Marine and Environmental Sciences and Marine Research Institute (INMAR), University of Cádiz, The European University of the Seas (SEA-EU), Puerto Real, E11510, Spain

* Corresponding author: Maurice R. Elphick.
Email: m.r.elphick@qmul.ac.uk

This PDF file includes:

- Supporting text
- Figures S1 to S17
- Tables S1 to S5
- Legends for Datasets S1 to S4
- SI References

Other supporting materials for this manuscript include the following:

- Datasets S1 to S4

Supporting Information Text

Results

Identification of peptides derived from the BN-type precursor in the starfish *A. rubens* (ArBNP)

Mass spectra from MS/MS analysis of synthetic ArBN revealed a mass/charge (884.46 m/z, 2+ ion) and fragmentation profile (with both b and y ions identified) consistent with the structure of this peptide (*SI Appendix,* Fig. S4*A*i) and a peptide with the same mass/charge and a similar fragmentation profile was detected in *A. rubens* radial nerve cord extract (*SI Appendix,* Fig. S4*A*ii, highlighted with a pink rectangle). Likewise, mass spectra from MS/MS analysis of synthetic $ArBN_{5-14}$ revealed a mass/charge (615.31 m/z, 2+ ion) and a fragmentation profile (with both b and y ions identified) consistent with the structure of this peptide (*SI Appendix,* Fig. S4*B*i) and a peptide with the same mass/charge and a similar fragmentation profile was detected in *A. rubens* radial nerve cord extract (*SI Appendix,* Fig. S4*B*ii, highlighted with an orange rectangle). In contrast, whilst mass spectra from MS/MS analysis of the synthetic peptide $ArBNP_{65-81}$ revealed a mass/charge (851.45 m/z, 2+ ion) and fragmentation profile (with both b and y ions identified) consistent with the structure of this peptide (*SI Appendix,* Fig. S4*C*i), this peptide was not detected in *A. rubens* radial nerve cord extract. However, a peptide with mass/charge (944.49 m/z, 2+ ion) and fragmentation profile (with both b and y ions identified) consistent with the sequence EPMPSSLALYIANLSPGK was detected in the extract of radial nerve cords from *A. rubens* that had been subjected to digestion with trypsin (*SI Appendix,* Fig. S4*C*ii, highlighted with a green rectangle). These findings demonstrate that the C-terminally amidated peptide ArBN and a peptide corresponding to the C-terminal region of ArBN ($ArBN_{5-14}$) are present in the radial nerve cords of *A. rubens*. However, these findings also indicate that the putative C-terminally amidated peptide $ArBNP_{65-81}$ may not be present in the radial nerve cords of *A. rubens.* Furthermore, whilst the peptide $ArBNP_{65-82}$ was detected in the extract of radial nerve cords from *A. rubens* that had been subjected to digestion with trypsin, this peptide was not detected in the extract that had not been subjected to trypsin digestion. These findings indicate that neither $ArBNP_{65-81}$ nor $ArBNP_{65-82}$ are derived from ArBNP in *A. rubens* physiologically.

Methods

Structural characterization of ArBNP-derived neuropeptides using mass spectrometry

To prepare an extract of *A. rubens* radial nerve cords, radial nerve cords were dissected from six adult specimens of *A. rubens* using a method described previously (1), and transferred to an extraction buffer comprising 90% HPLC-grade methanol, 9% water and 1% glacial acetic acid. After homogenization of the radial nerve cord tissue, the extract was centrifuged at 9000 rpm (PK 131R multispeed refrigerated centrifuge, ALC international) for 3 min at 10°C. The supernatant was bubbled with nitrogen gas to remove the solvent, leaving 2 ml of aqueous extract, which was then centrifuged for 3 min at 9000 rpm at 10°C to pellet any remaining solids. 1 ml of the supernatant was transferred to a fresh microcentrifuge tube for dehydration using a speed dry vacuum concentrator (RVC 2-18 CD plus mini concentrator, Martin Christ) for 10 h 30 min at 1750 rpm at room temperature. The dried extract was stored at -20°C and then thawed in a desiccator prior to transportation from Queen Mary University of London to the University of Warwick for MS analysis.

Samples of the *A. rubens* radial nerve cord extract were prepared by performing i). reduction and alkylation followed by trypsin digest; ii). reduction and alkylation only; iii). trypsin digest only; iv). no reduction or alkylation, and no trypsin digest, employing methods described previously (2), and then these samples were analysed using mass spectrometry (NanoLC-ESI-MS/MS). Samples of the synthetic peptides ArBN, $ArBN_{5-14}$ and $ArBNP_{65-81}$ were also analysed using NanoLC-ESI-MS/MS to enable comparison of spectra for these peptides with spectra for peptides present in the radial nerve cord extract. For NanoLC-ESI-MS/MS, an Ultimate 3000 RSLCnano system (Thermo Fisher Scientific) was coupled online to two different mass spectrometers: Hybrid TimsTOF Pro (Bruker Daltonics, Germany) (3) and Thermo Orbitrap Fusion (Q-OT-qIT, Thermo Fisher Scientific) (4). Reversed phase chromatography was used to separate peptides prior to mass spectrometric analysis. The mobile phase buffer A was 0.1% formic acid in

water and the mobile phase B was 0.1% formic acid in acetonitrile. Samples were loaded onto a µ-precolumn equilibrated in 2% aqueous acetonitrile containing 0.1% trifluoroacetic and peptides were eluted onto an analytical column by increasing the mobile phase B concentration from 4% B to 25% over 36 min, then to 35% B over 10 min, and to 90% B over 3 min, followed by a 10-minute re-equilibration at 4% B.

For mass spectrometry using Hybrid TimsTOF Pro (Bruker Daltonics, Germany) via a CaptiveSpray nano-electrospray ion source, two C18 columns were utilized: an Acclaim PepMap µ-precolumn cartridge 300 µm i.d. × 5 mm 5 µm 100 Å (Thermo Fisher Scientific) and a 75 µm × 40 cm 1.9 µm (Bruker nanoElute Forty Analytical column). The TimsTOF Pro was operated in Data-Dependent Parallel Accumulation-Serial Fragmentation (PASEF) mode. Peptides were separated by ion mobility depending on their collisional cross sections and charge states. The method settings were as follows: mass range 100 to 1700 m/z, ion mobility range 1/K0 Start 0.6 Vs/cm^2 End 1.6 Vs/cm^2, Ramp rate 9.42 Hz and Duty cycle 100%.

For mass spectrometry using Thermo Orbitrap Fusion (Q-OT-qIT, Thermo Fisher Scientific), two C18 columns were utilized: an Acclaim PepMap µ-precolumn cartridge 300 µm i.d. × 5 mm 5 µm 100 Å and an Acclaim PepMap RSLC 75 µm × 50 cm 2 µm 100 Å (Thermo Fisher Scientific). Survey scans of peptide precursors from 375 to 1575 m/z were performed at 120K resolution (at 200 m/z) with a 50% normalized AGC target and the maximum injection time was 150 ms. Tandem MS was performed by isolation at 1.2 Th using the quadrupole, HCD fragmentation with normalized collision energy of 33, and rapid scan MS analysis in the ion trap. The MS2 was set to 50% normalized AGC target and the maximum injection time was 200 ms. Precursors with charge state 2–6 were selected and sampled for MS2. The dynamic exclusion duration was set to 45 s with a 10 ppm tolerance around the selected precursor and its isotopes. Monoisotopic precursor selection was turned on. The instrument was run in top speed mode with 2 s cycles.

Raw data were converted to mascot generic format using MSConvert in ProteoWizard Toolkit (v. 3.0.5759) (5). MS spectra were searched with Mascot engine (Matrix Science, v. 2.4.1) (6) against a database comprising 40 *A. rubens* neuropeptide precursor proteins and all proteins in GenBank from species belonging to the family *Asteriidae* database. A no-enzyme search was performed with up to four missed cleavages and carbamidomethyl as fixed modification. Post-translational amidation by modification of C-terminal glycine residues and oxidation were included as variable modifications. Precursor mass tolerance was 20 ppm and product ions were searched at 20 ppm tolerances.

Scaffold (version Scaffold_5, Proteome Software Inc.) was used to validate MS/MS based peptide and protein identifications. Peptide identifications were accepted if they could be established at greater than 95.0% probability by the Scaffold Local FDR algorithm. Protein identifications were accepted if they could be established at greater than 95.0% probability and contained at least two identified peptides.

Identification and phylogenetic analysis of candidate BN-type receptors in the starfish *A. rubens* and in other echinoderms

It is reported that BN-type signaling is closely related to the vertebrate endothelin (ET)-type and protostome CCHa/EP-type signaling systems (7). Therefore, BN-type and ET-type receptor sequences from *H. sapiens* (NCBI accession no. NP_001948.1, NP_000106.1, NP_005305.1, NP_001718.1 and NP_002502.2) and CCHa-type receptor sequences from *D. melanogaster* (NCBI accession no. NP_611241.2 and NP_610199.2) were submitted as query sequences for BLAST analysis of *A. rubens* transcriptome/genome sequence data using the BLAST tool in TBtools-II (Toolbox for Biologists; v2.019) (8) with the number of threads set as 2, e-value as 1e-5 and number of hits as 50. BLAST analysis of transcriptome data identified transcripts encoding ten putative BN-type receptor related proteins (contigs 1114094, 1110162, 1113802, 1107822, 1112764, 1110464, 1122947, 1115257, 1118095, 1103008). Then BLAST analysis (https://blast.ncbi.nlm.nih.gov/) of the *A. rubens* genome sequence using the protein encoded by contig 1114094 as a query sequence identified two additional putative BN-type receptor related proteins (NCBI accession no. XP_033640355.1 and XP_033640356.1). Then, the amino acid sequences of these *A. rubens* BN-type receptor related proteins were analysed using Protter V1.0 (http://wlab.ethz.ch/protter/start/) to determine the locations of predicted membrane-spanning

domains and other structural features (9). The topology of ten of the potential *A. rubens* BN-type receptors as predicted by Protter V1.0 comprised seven transmembrane domains, as expected for a GPCR. However, the topology of the proteins encoded by contig 1110162 and contig 1110464 comprised only six and four transmembrane domains, respectively. Analysis of the transcript sequence encoding ArBNR2 (contig 1110162) revealed that it had an incomplete open reading frame encoding a 341-residue protein and analysis of this protein sequence using Protter V1.0 revealed an abnormal structure for a GPCR (*SI Appendix,* Fig. S5*B*). To determine the complete open reading frame of ArBNR2, contig 1110162 was used as a query for blastx analysis of *A. rubens* genome sequence data on NCBI and accession no. XP_033631283.1 was identified as the top hit. The protein (XP_033631283.1 referred to henceforth as ArBNR2) was analysed using Protter V1.0 and this also revealed an abnormal structure for a GPCR, with only six predicted transmembrane domains and with the C-terminus located extracellularly (*SI Appendix,* Fig. S15*A*). However, interestingly, analysis of the sequence of ArBNR2 using a different method for prediction of transmembrane helices in proteins (DeepTMHMM; https://services.healthtech.dtu.dk/services/DeepTMHMM-1.0/) revealed the presence of seven predicted transmembrane domains in ArBNR2. Because of this inconsistency in analysis of the ArBNR2 sequence, we investigated if this characteristic of ArBNR2 also occurs in closely related starfish species. To accomplish this, the protein sequence XP_033631283.1 was used as a query for tblastn analysis of the nucleotide collection (nr/nt) database on NCBI. A protein sequence from the starfish *Asterias amurensis* (referred to henceforth as AaBNR2) and a protein sequence from the starfish *Marthasterias glacialis* (referred to henceforth as MgBNR2) were found, two species that belong to the same family as *A. rubens* – the *Asteriidae*. Consistent with our findings for ArBNR2, analysis of the amino acid sequence of AaBNR2 using Protter revealed six predicted transmembrane domains (*SI Appendix,* Fig. S15*B*) and analysis of the amino acid sequence of AaBNR2 using DeepTMHMM revealed seven predicted transmembrane domains. In contrast, analysis of MgBNR2 using Protter (*SI Appendix,* Fig. S15*C*) and DeepTMHMM revealed that MgBNR2 contains seven predicted transmembrane domains with the N-terminus and C-terminus located extracellularly and intracellularly, respectively, as expected for a GPCR.

Having discovered that MgBNR2 has the expected topology for a GPCR when analysed using both Protter and DeepTMHMM, we then compared its sequence to ArBNR2. This revealed a notable difference in the sequences of the predicted sixth transmembrane domains (TMD6) of these proteins. In MgBNR2, TMD6 is predicted to be formed by twenty-eight residues (250 to 277), whereas in ArBNR2 TMD6 is predicted to be formed by nineteen residues (250 to 268). Furthermore, we noticed that residue 272 in MgBNR2, which is predicted to be located in TMD6, is glycine (G), whereas residue 272 in ArBNR2 is glutamate (E) and this is predicted to be located extracellularly. We hypothesized that this may explain the topology of ArBNR2 predicted by Protter and to test this hypothesis, we substituted Glu272 in ArBNR2 with a glycine residue and submitted this sequence (ArBNR2(G_{272})) for Protter analysis. This revealed that ArBNR2(G_{272}), like MgBNR2, has the expected topology for a GPCR with seven transmembrane domains and with the N-terminus and C-terminus predicted to be located extracellularly and intracellularly, respectively (*SI Appendix,* Fig. S15*D*). Furthermore, the codons for Glu272 in ArBNR2 and Gly272 in MgBNR2 are GAG and GGG, respectively, and therefore only a single nucleotide substitution is required to give rise to these amino acid differences.

Further analysis of the sequence of contig 1110464 and the corresponding genomic sequence revealed that it is a pseudogene. The protein encoded by the contig 1110464 is identical to the protein XP_033624147.1 (NCBI GenBank). Analysis of the sequence of XP_033624147.1 using Protter V1.0 revealed an abnormal topology, presenting only four predicted transmembrane domains, not as expected for a GPCR (*SI Appendix,* Fig. S5*L*). Therefore, XP_033624147.1 could be partial protein sequence. To obtain a potential complete sequence for this protein, its amino acid sequence was used as a query for tblastn analysis of the nucleotide collection (nr/nt) database on NCBI. A highly similar protein sequence was found in the starfish *Asterias amurensis* (encoded by a gene on chromosome 5; AaChr5), a species that belongs to the same family as *A. rubens* – the *Asteriidae*. The nucleotide sequence encoding this protein (bases 11,353,641 to 11,354,759 on AaChr5) was translated to a protein sequence (referred to as AaBNR12) using Expasy translate (https://web.expasy.org/translate/), which revealed this protein is encoded by a single exon. Analysis of the amino acid sequence of AaBNR12 by Protter V1.0 revealed that it contains seven

predicted transmembrane domains, as expected for a GPCR. Having discovered that AaBNR12 has the expected topology for a GPCR, this sequence was used as a query for tblastn analysis of *Asterias rubens* genome data on NCBI to obtain a potential full-length sequence for the protein XP_033624147.1. Protein sequences encoded by bases from 13,641,840 to 13,642,439 and from 13,641,325 to 13,641,852 on the *A. rubens* chromosome 3 (ArChr3) were found to correspond with the sequence of AaBNR12. Then, we compared the nucleotide sequences on AaChr5 ranging from 11,353,641, to 11,354,759 and ArChr3 ranging from 13,641,325, to 13,642,439. The sequence alignment revealed that in *A. rubens* there is a deletion of a nucleotide in the position corresponding to nucleotide 601 in *A. amurensis*, which causes a frameshift and introduction of a premature stop codon. Therefore, this confirmed that the *A. rubens* ortholog of the gene encoding AaBNR12 in *A. rubens* is a pseudogene and therefore henceforth we refer to it as *ArBNRP.*

Hereafter we refer to the eleven *A. rubens* BN-type receptor candidates as ArBNR1 (contig 1114094), ArBNR2 (contig 1110162), ArBNR3 (contig 1113802), ArBNR4 (contig 1107822), ArBNR5 (XP_033640355.1), ArBNR6 (XP_033640356.1), ArBNR7 (contig 1112764), ArBNR8 (contig 1122947), ArBNR9 (contig 1115257), ArBNR10 (contig 1118095) and ArBNR11 (contig 1103008). Furthermore, analysis of transcriptome/genome sequence data (https://blast.ncbi.nlm.nih.gov/) enabled identification of homologs of the *A. rubens* BN-type receptors in other echinoderms, including the crown-of-thorns starfish *Acanthaster* cf. *solaris*, the brittle star *Ophionotus victoriae*, the sea urchin *Strongylocentrotus purpuratus* and the sea cucumber *Apostichopus japonicus*. With the exception of BN-type receptor 1, other putative BN-type receptors identified in other echinoderms were named using the last four numbers of their protein accession numbers. Putative BN-type receptor 1 in other echinoderms were named in accordance with their relationships to the BN-type receptor 1 in *A. rubens* (ArBNR1). Analysing radial nerve cord transcriptome data from *A. rubens*, contig 1114094 was identified as a transcript encoding a bombesin-type receptor 1 in *A. rubens* (ArBNR1). Protein XP_033635529.1 on NCBI comprises only a partial sequence of the ArBNR1 protein encoded by contig 1114094. Accordingly, analysis of the protein XP_033635529.1 using Protter V1.0 revealed an abnormal topology, presenting only six predicted transmembrane domains, not as expected for a GPCR. Using the ArBNR1 amino acid sequence as a query for blastp analysis of the non-redundant protein sequence (nr) database for Echinodermata (taxid:7586) on NCBI, orthologous proteins were found in the sea cucumber *Apostichopus japonicus* (PIK58426.1) and the sea urchin *Strongylocentrotus purpuratus* (XP_785425.3). But, like the protein XP_033635529.1 in *A. rubens*, these proteins were found to be partial sequences. To obtain the full-length sequences of bombesin-type receptor 1 in *A. japonicus* and *S. purpuratus*, the ArBNR1 amino acid sequence was used as a query for tblastn analysis of RefSeq Genome Database (refseq_genomes) of Echinodermata (taxid:7586) on NCBI. This enabled the full-length sequence of bombesin-type receptor 1 in *S. purpuratus* (Spur_BNR1) to be identified. Furthermore, by manually checking the genome sequence of *S. purpuratus*, the exon/intron splice sites were identified (10) for Spur_BNR1. Then, the full-length sequence of Spur_BNR1 was analysed using Protter V1.0, which revealed that it comprises seven transmembrane domains, as expected for a GPCR. As tblastn analysis of RefSeq Genome Database (refseq_genomes) of Echinodermata (taxid:7586) did not identify the full-length sequence of bombesin-type receptor 1 in *A. japonicus*, the protein sequence encoded by second exon of the ArBNR1 gene was used as a query for tblastn analysis of Sequence Read Archive (SRA) SRX18487905 for *Apostichopus japonicus* (taxid:307972). Sequence ID: SRA:SRR22523578.142727211.1 was identified as a hit and then this sequence was found in assembled genome sequence data for *Apostichopus japonicus*. Then, by manually checking the genome sequence of *Apostichopus japonicus*, the exon/intron splice sites for the gene encoding Ajap_BNR1 were identified. Ultimately, the full-length sequence of Ajap_BNR1 was determined and then analysed using Protter V1.0, which revealed it comprises seven transmembrane domains, as expected for a GPCR. The full-length DNA sequences and amino acid sequences for Spur_BNR1 and Ajap_BNR1 are shown in *SI Appendix,* Fig. S16.

To investigate relationships between the putative BN-type receptors in *A. rubens* and other echinoderms and BN-type, ET-type and CCHa/EP-type receptors in other taxa (*SI Appendix,* Table S4), CLuster ANalysis of Sequences (CLANS) was performed, with elevenin (ELev)-type receptors and orexin-type receptors included as outgroups. CLANS is a tool for visualization of all-against-all pairwise protein sequence similarities in a Fruchterman-Reingold force directed graph (11). CLANS

was performed in the MPI Bioinformatics Toolkit (https://toolkit.tuebingen.mpg.de/tools/clans) with Scoring Matrix set at BLOSUM62 and Extract BLAST HSP up to e-values of 1e-40. Clustering was first run in three dimensions, and then the map was collapsed to a two-dimensional diagram.

As a complement to use of CLANS, phylogenetic trees were generated with the maximum-likelihood method (1000 bootstrap replicates) using One Step Build a ML Tree plugin in TBtools (12), specifying orexin-type receptors as an outgroup. First a phylogenetic tree was generated using the same sequences used for CLANS, which include all eleven of the potential BN-type receptors in *A. rubens* and homologs of these receptors in other echinoderms (tree_A). Then, informed by findings from tree A, branches of this tree containing subsets of putative echinoderm BN-type receptors were individually subjected to further phylogenetic analysis together with BN-type, ET-type and CCHa/EP-type receptors from other taxa and with elevenin (ELev)-type receptors and orexin-type receptors included as outgroups. Tree_B analysed the branch of tree_A containing ArBNR1; tree_C analysed the branch of tree_A containing ArBNR2; tree_D analysed the branch of tree_A containing ArBNR3; tree_E analysed the branch of tree_A containing ArBNR4, ArBNR5 and ArBNR6; tree_F analysed the branch of tree_A containing ArBNR7; tree_G analysed the branch of tree_A containing ArBNR8; tree_H analysed the branch of tree_A containing ArBNR9 and tree_I analysed the branch of tree_A containing ArBNR10 and ArBNR11.

Functional characterization of *A. rubens* BN-type receptors

Two cDNAs encoding ArBNR1 and ArBNR3 were cloned from *A. rubens* radial nerve cord cDNA using Q5 polymerase (NEB, Cat. no. M0491S) and PCR with specific primers (*SI Appendix,* Table S5). To facilitate expression of the cloned receptors, the forward primers were designed to contain a partial Kozak consensus sequence (ACC) before the ATG start codon. Then, PCR products were inserted into the mammalian expression vector pcDNA 3.1(+) (Invitrogen, Cat. no. V79020) that had been cut in advance with the restriction enzyme EcoRV (NEB, Cat. no. R3195T) by performing blunt-end ligation with T4 DNA ligase (NEB, Cat. no. M0202S). Successful ligation and the direction of the insert was determined by restriction enzyme digestion and sequencing (TubeSeq service; Eurofins Genomics).

As described above, the protein ArBNR2 encoded by contig 1110162 revealed an abnormal predicted topology for a GPCR (*SI Appendix,* Fig. S5*B*). However, the sequence of ArBNR2 encoded by contig 1110162 (derived from neural transcriptome data) was found to be identical to residues 18 - 270 of the protein XP_033631283.1, which was determined from genome sequence data. Comparative analysis of starfish genome sequence data revealed that a single amino acid at position 272 in ArBNR2 affects the predicted topology of this protein. Therefore, we tested two forms of ArBNR2: firstly, the naturally occurring protein (ArBNR2) and secondly, a modified form of ArBNR2 in which the naturally occurring residue at position 272 (glutamate) was replaced with glycine ArBNR2(G_{272}) and which has seven predicted transmembrane domains. The cDNA sequences of ArBNR2 and ArBNR2(G_{272}) were codon-optimized for expression in mammalian cells, custom synthesized with a partial Kozak sequence (GCCACC) added before the ATG start codon and cloned into pcDNA3.1(+) vector (using BamHI and XhoI sites) in the forward orientation (GenScript Biotech Corporation, Hong Kong, China). The non-codon optimized and codon-optimized sequences are shown in the *SI Appendix,* Fig. S17. Midi-preps of the plasmids pcDNA3.1(+)_ArBNR2 and pcDNA3.1(+)_ArBNR2(G_{272}) were prepared using a MACHEREY-NAGEL Plasmid Midi Kit (Qiagen, Germany) and sequenced using primers CMVF_pCDNA3 (5′-CAACGGGACTTTCCAAAATG-3′) and BGHRev (5′-TAGAAGGCACAGTCGAGG-3′) (Source Bioscience, UK).

To functionally characterize the potential *A. rubens* BN-type receptors, Chinese hamster ovary (CHO)-K1 cells stably expressing the calcium-sensitive bioluminescent reporter GFP-aequorin fusion protein (G5A) were used as an expression system (13, 14). Previous studies have revealed that BN-type receptors couple via Gαq-type and Gαs type G proteins to stimulate activation of phospholipase-C (PLC)/Ca^{2+} signaling or adenylyl cyclase/cyclic AMP signaling, respectively (15-19). Therefore, for receptor assays we transfected CHO-K1 cells with the chimeric G-protein Gqs5, which enables GPCRs that preferentially couple with Gαs to signal via PLC/Ca^{2+} signaling (20). CHO-K1 cells were cultured in Dulbecco’s Modified Eagle’s Medium (DMEM)/F12 (Thermo Fisher Scientific, Cat. no. 11039047) supplemented with 10% of fetal bovine serum (Thermo Fisher Scientific, Cat. no. 10082147), Antibiotic-Antimycotic 1x (Thermo Fisher Scientific,

Cat. no. 15240062) and 30 µg/ml Geneticin (Thermo Fisher Scientific, Cat. no. 10131035) in a T25 flask at 37°C with 5% carbon dioxide (CO_2). When cell confluency reached 80%, 5 µg of pcDNA3.1(+) plasmid encoding a potential BN-type receptor or empty pcDNA3.1(+) vector (negative control) was co-transfected with 1 µg of plasmid encoding Gqs5 (Addgene plasmid #24498; http://n2t.net/addgene:24498; RRID: Addgene_24498) using Lipofectamine 3000® Transfection Kit (Thermo Fisher Scientific, Cat. no. L300008). The transfected cells were cultured for 48 h after transfection. Then, the cells were detached using PBS/EDTA buffer at pH 7.4 (Thermo Fisher Scientific, Cat. no. 10010023) and collected. 7 µl of 1 mM coelenterazine-H (Thermo Fisher Scientific, Cat no. C6780) dissolved in methanol was added to the harvested cells to incubate for 2 h with gentle stirring in the dark. After this, the cells were diluted with an additional 6 ml of BSA medium and left for a further 30 min with stirring. 50 µl of synthetic ArBN (EPRRNYNRVFGPTY-NH_2), $ArBN_{5-14}$ (NYNRVFGPTY-NH_2) or $ArBNP_{65-81}$ (EPMPSSLALYIANLSP-NH_2) were added to 96-well plates (Costar Assay microplate, REF: 3903/Falcon 96 well microplate, Code: 353377) at a range of concentrations. A FLUOstar Omega or a VANTAstar plate reader (BMG LABTECH, Germany) was set up to inject a fixed volume (50 µl) of cells into each well containing different concentrations of ArBN, $ArBN_{5-14}$ or $ArBNP_{65-81}$. A program was established to record the luminescence values over a 35 s period after the cells were injected into one well of the plate. The total luminescence of each well over the 35 s period was determined and normalized to the maximum value obtained in each experiment (100% activation) and to the value obtained with vehicle media (0% activation). The viability of cells and 100% activation in tests where no dose-dependent luminescence responses to peptides were observed were determined by detection of Triton-X100-induced luminescence. Dose-response curves were plotted with a four-parameter logistic curve and luminescence responses with 10^{-5} M ArBN and vehicle media were statistically analysed by Student's *t*-test using Prism 10 (GraphPad software, USA). Data were presented as means (± SEM).

Mapping the expression of ArBNP transcripts in *A. rubens* using mRNA *in situ* hybridization

Antisense and sense probes were generated by *in vitro* transcription in a mixture containing 1 µg of purified PCR product, 2 µl of DIG RNA Labelling Mix (Roche, Cat. no. 11277073910), 2 µl of 0.2 M dithiothreitol (DTT) (Promega, Cat. no. P1171), 0.5 µl of placental ribonuclease inhibitor (10 U/µl; NEB, Cat. no. M0307S), 0.5 µl of T3 (50 U/µl; antisense probes; NEB, Cat. no. M0378S) or T7 RNA polymerase (50 U/µl; sense probes; NEB, Cat. no. M0251S), 2 µl of 10× transcription buffer (NEB, Cat. no, M0251S) at 37°C in a water bath for 2 h. Analysis of the sequence of the cloned ArBNP cDNA inserted in the pBluescript II SK(+) vector revealed that cloned ArBNP cDNA is inserted in the forward direction in the pBluescript II SK(+) vector. Thus, after linearization of the vector, T3 polymerase could be used to generate anti-sense probes and T7 polymerase could be used to generate sense probes. Following digestion of the DNA templates with RNAse free DNAse (NEB, Cat. no. M0303S) at 37°C in a water bath for 30 min, the labelled RNA probes were made up to total volume of 200 µl using distilled water and precipitated by adding 20 µl of 3 M sodium acetate (pH 5.5) and 550 µl 100% ethanol at -80°C for 1 h. Then, the solution mixture was centrifuged and the pellet was washed with freezer-cold 70% ethanol and centrifuged again. The pellet was left at room temperature to air dry for 30 min and then stored in 25% formamide (VWR Chemicals; Cat. no. 0606-500ML) made up in saline-sodium citrate buffer (2x SSC), with final probe concentration at around 100 ng/µl at -20°C.

Three starfish (*A. rubens*, diameter 3-4 cm) dissected into arms and central disks were fixed in 4% paraformaldehyde (PFA; Sigma-Aldrich, Cat. no. P6148-1KG) overnight at 4°C. The next day, the starfish were decalcified in Morse's solution (10% sodium citrate; 20% formic acid) for 10 h (central disks) or 8 h (arms), changing the solution every two hours. After decalcification, the specimens were washed two times for 10 min each in PBS and dehydrated through an ascending series of ethanol (30%, 50%, 70%, 90%, 100%), with 30 min for each step. The dehydrated tissue was then cleared twice for 10 min in fresh 100% xylene. The tissue was incubated in melted filtered paraffin wax at 60°C three times for 1 h each and finally embedded in paraffin wax. Sectioning was performed on a microtome (RM 2145, Leica Microsystems, UK), with

14 µm sections mounted over water on Poly-L-lysine coated microscope slides (Polysine; VWR Chemicals, UK) at around 50°C and then left to dry overnight at room temperature.

Slides were dried at 60°C for more than 1 h and then deparaffinized in xylene (3 × 7 min), rehydrated through a descending ethanol series (100%, 90%, 70%, 50%, 25%; 7 min for each step) and PBS twice for 5 min each. After rehydration, the tissue was fixed using 4% PFA/PBS for 20 min and then the PFA was washed away in PBS three times for 5 min each. The slides were treated with proteinase K (Qiagen, Cat. no. 19131) at a concentration of 10 µg/ml at 37°C for 15 min. The sections were post-fixed using 4% PFA/PBS for 10 min and washed two times with PBS to remove PFA. The slides were acetylated in acetylation solution (1.325% triethanolamine, 0.25% acetic anhydride and 0.175% acetic acid) for 10 min and washed in PBS twice for 5 min each. A pre-hybridization step was performed using 500 µl hybridization buffer (50% formamide; 5× SSC; 500 µg/ml yeast RNA; 50 µg/ml heparin; 0.1% Tween-20 in dH_2O) in a humidified chamber for 2 h. Next, 150 µl hybridization buffer with the DIG-labelled mRNA probes (1000 ng/ml) was added to the slides to incubate in a humidified chamber at 52°C for 36 h. The next day (second day) the slides were washed twice with 5× SSC buffer for 20 min each and then 0.2× SSC buffer for 40 min each time. The slides were equilibrated in buffer B1 (0.1 M Tris pH 7.5, 0.15 M NaCl) for 10 min. Then the slides were blocked using blocking buffer (5% goat serum diluted in buffer B1) for 2 h at room temperature. Next, the sections were incubated with alkaline phosphatase-conjugated anti-DIG antibody (1:3000; Roche, Cat. no. 11093274910) in a humidified chamber overnight at 4°C. The following day (third day), the slides were washed twice with B1 buffer for 10 min to remove unbound antibodies. Buffer B3 (0.08 M Tris pH 9.5, 0.08 M NaCl, 0.04 M $MgCl_2$) was used to equilibrate the slides for 10 min. Then, a staining solution (17.5 µl per ml of NBT (75 mg/ml in 70% dimethylformamide) and BCIP (50 mg/ml 22.5 µl per ml diluted in buffer B3) was added to the slides in a humidified chamber at room temperature until staining was observed. The slides were then washed with distilled water three times for 5 min each and dried on a hot plate at 65°C for 10 min, cleared in xylene twice for 5 min and mounted with 400 µl of histomount mounting media (Sigma-Aldrich, Cat. no. 06522).

Images of stained sections were captured with a QIClich CCD Colour Camera (Qimaging, UK) linked to a DMRAI light microscope (Leica Microsystems, UK) and using Volocity v. 6.3.1 image analysis software (Perkin-Elmer, USA) running on an iMac computer (27 inch with OS Yosemite, v. 10.10).

Generation and characterization of antisera to ArBN and ArBNP

To enable the localization of ArBN peptide expression in *A. rubens* using immunohistochemistry, an antiserum to the peptide ArBN was generated. An antigen peptide (ArBNag; KNYNRVFGPTY-NH_2) was custom synthesized by (PPR Ltd., UK), incorporating an N-terminal lysine residue (K) providing a reactive amine group for coupling to a carrier protein (keyhole limpet hemocyanin (KLH); Sigma-Aldrich, UK). Conjugation of ArBNag to KLH was done using 5% glutaraldehyde (Sigma-Aldrich, UK) in phosphate buffer (0.1 M; pH 7.2) and then the conjugate was used for rabbit immunization (performed by Charles River Labs, UK). On day 0, pre-immune serum was collected and then booster immunizations were administered on days 28, 42, and 56. Antiserum was collected on days 36 and 53 for testing and then a final bleed was collected on day 75. To test for production of antibodies to ArBNag during and after immunization, enzyme-linked immunosorbent assays (ELISA) were performed to test serum samples.

Because generation of an antiserum to ArBNag (KNYNRVFGPTY-NH_2) was unsuccessful (SI Appendix, Fig. S11*A*), an alternative experimental approach for neuropeptide immunohistochemistry was used by generating an antiserum to a peptide corresponding to the C-terminal region of ArBNP. This experimental approach has been used successfully recently for other neuropeptides in *A. rubens* (21). The antigen peptide (ArBNPag; KLAMTRMNSEAENE) was custom synthesized (PPR Ltd., UK). and conjugated to thyroglobulin (Sigma-Aldrich, UK) as a carrier protein using 5% glutaraldehyde (Sigma-Aldrich, UK) in phosphate buffer (0.1 M; pH 7.2). Then the conjugate was used for rabbit immunization (Charles River Labs, UK). On day 0, pre-immune serum was collected. Booster immunizations were administered on days 29, 43, and 56. Antiserum samples were collected on day 36 and 50 and a final bleed was collected on day 70. To test for production of antibodies to ArBNPag during the immunization, ELISA was performed to test the collected sera. Antibodies to ArBNPag were purified from the final bleed antiserum by affinity

purification using the AminoLink Plus Immobilization Kit (Thermo Fisher Scientific, Cat. no. 44894), with bound antibodies eluted using trimethylamine (TEA) elution buffer (6.3 ml of TEA (Sigma-Aldrich, Cat. no. 121-44-8), and 0.7 ml of Tris (1 M, pH = 7.0)). Eluates were dialyzed and sodium azide (0.1%) was added for long-term storage of the affinity-purified polyclonal antibodies at 4°C. The rabbit antiserum to ArBNP has been assigned the RRID: AB_3674122.

Immunohistochemical localization of ArBNP in *A. rubens*

Three small specimens of *A. rubens* (3-4 cm in diameter) were fixed in Bouin's solution (75% saturated picric acid in seawater, 25% formalin, 5% acetic acid) for 3 days at 4°C. Then the arms and the central disk were dissected apart and decalcified in 1% ascorbic acid/0.15 M sodium chloride solution at 4°C for at least 2 weeks. After decalcification, the specimens were dehydrated through an ethanol series, embedded in paraffin and then 8 μm sections were cut using a microtome (RM 2145, Leica Microsystems, UK) and mounted on chrome alum/gelatin-coated microscope slides.

Wax was removed from slides using xylene (3 × 10 min) and then slides were immersed in 100% ethanol (2 × 10 min). 0.3% hydrogen peroxide (VWR Chemicals, UK) in methanol was used to quench endogenous peroxidases for 30 min and then an ethanol series (90%, 70%, 50%), distilled water and PBST were used to rehydrate the tissue sections. 5% goat serum (Sigma-Aldrich, UK) made up in PBST was used for blocking for 2 h at room temperature. Then the slides were incubated with affinity-purified rabbit polyclonal antibodies to ArBNP (TEA fraction diluted 1:30 in 5% NGS/PBST) at 4°C overnight. The next day, the slides were washed with PBST (2 × 5 min and then 3 × 10 min) and incubated with Peroxidase-AffiniPure Goat Anti-Rabbit IgG (H + L) conjugated to Horseradish Peroxidase (RRID: AB_2313567; Jackson ImmunoResearch, West Grove, PA) diluted 1:2000 in 2% goat serum/PBST for 3 h. Bound antibodies were revealed using a solution comprising 0.05% diaminobenzidine (VWR Chemicals, UK), 0.05% nickel chloride (Sigma-Aldrich, Gillingham, UK), 0.015% hydrogen peroxide (VWR Chemicals, UK) diluted in PBS. Once staining was observed, slides were washed in distilled water (2 × 10 min) and dehydrated in an ethanol series (50%, 70%, 90%, 100%) for 10 min each. After cleaning in xylene (2 × 10 min), the slides were mounted with coverslips using DPX mounting medium (Thermo Fisher Scientific). Slides tested with primary antibodies that had been pre-adsorbed with the antigen peptide at a concentration of 20 μM were prepared to assess the specificity of immunostaining. Images of stained sections were captured as described above for mRNA *in situ* hybridization.

Analysis of *in vitro* effects of ArBN on cardiac stomach and tube foot preparations from *A. rubens*

For cardiac stomach preparations, a cardiac stomach and its associated pyloric stomach were dissected from the central disk of the starfish. The aboral side of the cardiac stomach and the oesophagus (oral side) were tied with cotton ligatures. Then the oral ligature was tied to a fixed metal hook and the aboral ligature was tied to a high-grade isotonic transducer (MLT0015; ADInstruments Ltd; Oxford, UK). For tube foot preparations, firstly a region of the ambulacrum containing a cluster of tube feet was dissected from one arm of *A. rubens*. Then, the body wall and one tube foot with its intact associated ampulla were retained while other tube feet and ampullae were removed. The ambulacral body wall was tied with a cotton ligature and then tied to a fixed metal hook. The disk of the tube foot was tied with a cotton ligature and the other end of the ligature was tied to a high-grade isotonic transducer (MLT0015; ADInstruments Ltd; Oxford, UK).

All the dissected preparations were set up in an aerated organ bath filled with 20 ml artificial seawater maintained at approximately 11°C. Changes in the contractile state of preparations were detected by the transducer, which was linked via a bridge amplifier (FE221 Bridge Amp, ADInstruments Ltd, Oxford, UK) to data acquisition hardware (PowerLab 2/36, ADInstruments Ltd, Oxford, UK). Data recording, visualization and analysis were acquired by LabChart (v. 8.0.7) software running on a laptop (Lenovo E540, Windows 7 Professional). Preparations were allowed to equilibrate until a stable baseline length was achieved after they were set up in the organ bath. NGFFYamide is known to cause contraction of cardiac stomach preparations (22) and acetylcholine (ACh) is known to cause contraction of tube foot preparations (23). Therefore, to check the viability of preparations and to normalize responses to peptides tested, 10^{-7} M NGFFYamide was tested on cardiac stomach preparations and 10^{-5} M of ACh was tested on tube

foot preparations. Having determined that ArBN had effects on preparations when tested at a high concentration (10^{-6} M), then dose-response data for this peptide were obtained with final concentrations in the organ bath ranging from 10^{-10} M to 10^{-6} M. Two types of tests were performed when obtaining dose-response data for ArBN:

Test type 1: Here the preparation was washed thoroughly with seawater after adding ArBN to the organ at different concentrations. In this way, effects of the peptide at each concentration were reversed by washing before the next concentration was tested.

Test type 2: Here the preparation was not washed in between application of ArBN at different concentration. Thus, cumulative dose-response data were obtained in these experiments.

The contraction effect of NGFFYamide (10^{-7} M) or ACh (10^{-5} M) was defined as 100% and the effects of ArBN were calculated as a percentage of the effect of NGFFYamide or ACh. Dose-response curves were generated using the four-parameter logistic curve in Prism 10 (GraphPad software, USA). Data were presented as means (± SEM).

Analysis of the *in vivo* effects of ArBN on the everted cardiac stomach of *A. rubens*

The physiological status of the starfish (~8 cm in diameter) tested was normalized by a 7-day starvation period. The starved starfish was placed oral side down and immersed in 2% magnesium chloride ($MgCl_2$) dissolved in artificial seawater, which causes muscle relaxation in the marine invertebrates (24) and typically causes cardiac stomach eversion in *A. rubens* within 30 min (22). When the cardiac stomach started to evert, the starfish was placed upside down (aboral side down) until the cardiac stomach was completely and stably everted. Then, the starfish was firstly injected with 10 µl of distilled water (negative control) and video recorded using a camera (Canon EOS 700D) for 7 min. Then, the same starfish was injected with NGFFYamide (positive control) or ArBN and video recorded for 7 min. Starfish (*A. rubens*) with a diameter of ~8 cm typically contain ~10 ml perivisceral coelomic fluid. Therefore, to achieve an estimated final concentration *in vivo* equivalent to that at which NGFFYamide (22) and ArBN (this study; Fig. 6*B*) exhibited a maximum contracting effect on cardiac stomach preparations *in vitro* (10^{-7} M), 10 µl of 10^{-4} M NGFFYamide or 10^{-4} M ArBN was injected into starfish. All the injections were performed using a Hamilton 75N 50 µl syringe (Sigma-Aldrich, UK) and all test agents were injected into the perivisceral coelom of starfish, taking care not to inject into the cardiac stomach. The *in vivo* effects of the peptides tested on *A. rubens* were analysed from the recorded videos. Images were captured from the videos every 30 seconds and the two-dimensional area of the everted cardiac stomach was measured using image J software (https://imagej.nih.gov/ij/). Retraction-inducing effects were calculated as a percentage of the cardiac stomach area just before injection. Graphs were generated using the nonlinear regression curve fit in Prism 10 (GraphPad software, USA). Data were presented as means (± SEM).

Analysis of the *in vivo* effect of ArBN on feeding behavior of *A. rubens*

Medium-sized intact adult starfish (n = 108) that exhibited a normal righting response (25) and normal feeding behavior on a mussel after 30 days of starvation were selected and starved for another 30 days. After 27-days of starvation, the starfish were transferred to and kept individually in Plexiglas aquaria (27.5 × 19 × 19.6 cm) filled with 6 L of seawater and gravel (Tropical Marine Centre™ Gravel Coarse #5) on the base. The base and sides of the aquaria were covered with black plastic, as described previously (26). After 3 days of acclimation (30 days of starvation at this point), 8 or 10 starfish per experiment were divided into a control group (injection of 10 µl of distilled water; n = 4 or 5) and a test group (injection of 10 µl of 10^{-4} M ArBN; n = 4 or 5). Ten minutes after the injection, one mussel (20-30 mm) without any encrusting organisms attached (27) was placed at one end of the tank, whilst the starfish was placed at the opposite end with one arm touching the wall of the tank and the madreporite directed toward the mussel. The time taken for starfish to first touch a mussel and the time to enclose the mussel were recorded. Twenty-four hours later the tank was checked to determine whether the starfish had successfully fed on the mussel. These experiments were repeated 11 times (n = 108 in total). However, four starfish that took more than 10 h to feed on mussels were removed from data analysis. Therefore, results from a total 104 starfish were used for data analysis. The proportion of the starfish that did not touch the mussel (total n = 20 for ArBN group; total n = 17 for control group) and the proportion of the starfish that did not feed on the mussel (total n = 36 for ArBN group; total n = 36 for control group) were

calculated for each experiment. Both for starfish that did feed and for starfish that did not feed, the time taken to first touch the mussel was also calculated. For starfish that successfully fed on the mussels, the time taken to enclose the mussel was calculated.

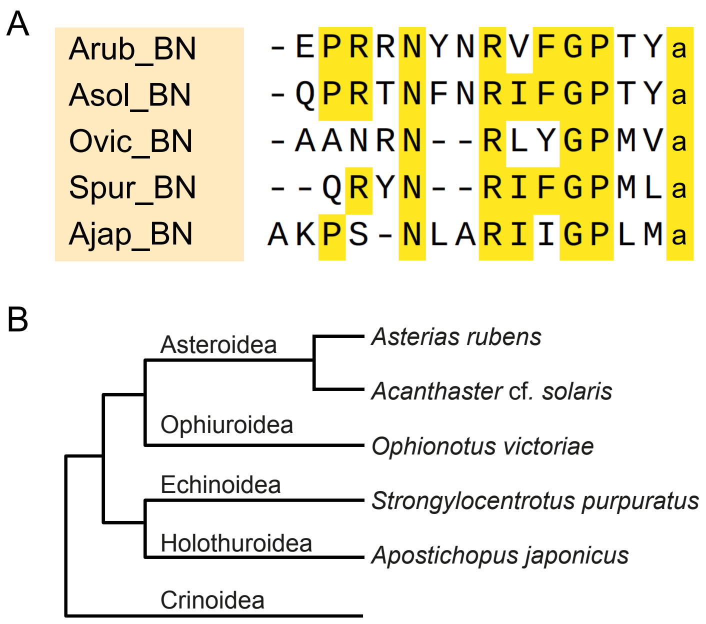

Fig. S1. (A) Sequence alignment of putative BN-type neuropeptides from *A. rubens* and other echinoderm species. A consensus N-terminal PRXN motif and a consensus C-terminal RIFGPXXa motif, where X is variable and “a” is a C-terminal amide predicted to be derived from glycine in the precursor proteins, are highlighted in yellow. The alignment shows high sequence conservation across echinoderm BN-type neuropeptides. Species and peptide name abbreviations are as follows: Ajap (*Apostichopus japonicus*), Arub (*Asterias rubens*), Asol (*Acanthaster* cf. *solaris*), Ovic (*Ophionotus victoriae*), Spur (*Strongylocentrotus purpuratus*), BN (Bombesin). (B) Dendrogram showing phylogenetic relationships of the five extant classes of the phylum Echinodermata, including representative species from the sequence alignment in panel A. Branch lengths are arbitrary but the topology is based on previously determined phylogenetic relationships (28). Note that BN-type neuropeptide precursors have yet to be identified in crinoids.

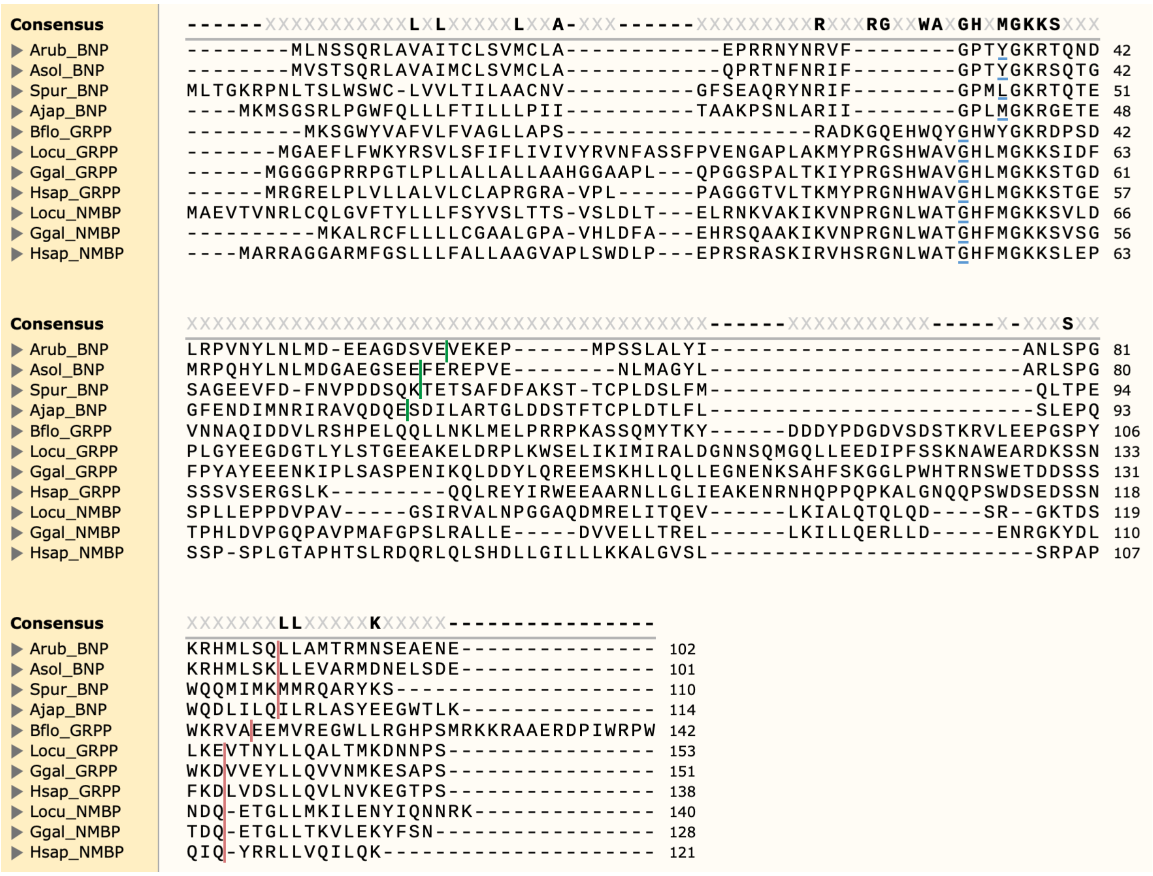

Fig. S2. Sequence alignment of putative BN-type precursors in echinoderms and BN-type precursors in chordates, showing the position of introns that interrupt the coding sequence in the corresponding genes. The position of the first intron (phase 1) in both echinoderms and chordates is shown by underlining with a blue line the amino acid whose corresponding codon is interrupted by the intron. The position of the second intron (phase 0) in echinoderms is marked with a vertical green line. The position of the third intron (phase 0) in echinoderms and the second intron in chordates is marked with a vertical red line. This shows that the locations of the first and third introns of genes encoding putative precursors of BN-type neuropeptides in echinoderms are consistent with those of the first and second introns in chordate BN-type precursor genes, respectively. The alignment of the precursor amino acid sequences was performed using the MUSCLE method plugin in SnapGene (https://www.snapgene.com/). The consensus threshold is >50%. Full names of species: Ajap (*Apostichopus japonicus*), Arub (*Asterias rubens*), Asol (*Acanthaster* cf. *solaris*), Bflo (*Branchiostoma floridae*), Ggal (*Gallus gallus*), Hsap (*Homo sapiens*), Locu (*Lepisosteus oculatus*), Spur (*Strongylocentrotus purpuratus*). Full names of peptide precursors: BNP (Bombesin precursor), GRPP (Gastrin-releasing peptide precursor), NMBP (Neuromedin B precursor).

1 gactcgtcggacggcaagtgtgcagaagaaaacacggggcctaagaaaacgcattggaga

61 gagaaaacagcacaagcgcacgaagaacgcccgcgtacaaaccaacaactatatacagcc

121 cgcattctgtaccaaagcagtgcacgtaa**ccacatcaccgtgaacgact**ttctttcttac

181 gtcttgagtttgctcttcgatttagaactttgcaaaaagccttctttgaggctgattagt

241 gggctagttggtctttgtctcaagactttgtaaatagttccttttggggggtgtttgtcta

301 ctcacaacgaagatgttaaactcatctcaacggctggctgtggctataacgtgcctctcg

M L N S S Q R L A V A I T C L S 16

361 gtgatgtgccttgcagaaccaagaaggaactacaacagagtctttggacccacatatggt

V M C L A E P R R N Y N R V F G P T Y G 36

421 aaaagaacacagaatgacctgaggccggtaaactatcttaatttgatggacgaagaagct

K R T Q N D L R P V N Y L N L M D E E A 56

481 ggggattcggtagaggttgaaaaggagcctatgccaagctcactagcgttgtacatcgcc

G D S V E V E K E P M P S S L A L Y I A 76

541 aacctctcaccagggaagaggcatatgttatcacagttacttgccatgacaagaatgaac

N L S P G K R H M L S Q L L A M T R M N 96

601 agtgaagccgagaatgagtgagacggagaccgtctggcgaccgcctcgtctaagtctggc

S E A E N E * 102

661 gactgactcgtctaagtcttaagaacagg**gtcttaccctaacaacgggga**tcaaacaaaa

721 aacaacaaaagtgatttttttttcgaatacaaaattttaaatgaattaaacgacctatgca

781 tagcgcaacgactataaaaggactgataaatataaatactgacgcgttattcaatgcaat

841 tgacctttgaccttttgtttatctagccaactctgttattcaaaatttgcattttgataa

901 ttagtttgggtgcatctttggatagatctctaaaaacaaagaaaccggaaaataaactca

961 taataattgaaatgataagttccgaccaatatccaaatttgaagaaaaaaaacaaatctt

1021 tagaattattgaataaattgtgggtttgtaagctaggtaggaaggtgtaccattggtggt

1081 tttcgggtagtttgttattttaaataatccattagcagtttaaactataagggtatcctt

1141 tacttaagttttcttttcaaaattgttaaaaagttttgtctaaattttcagtaattctca

1201 atcccaaaacacaacgcggaaactattaaaattaaataaaataaaccgttaaaacaacac

1261 ccctggacaaaaacttctgtgaaaattaattgtttgaactaattaaacctttctcgtgag

1321 tctggtaccccgtcgtcacatgctttacggatctgcatttatatgggaccagttttaaag

1381 aaaccgaataccaccaatgttacactttccctttgaaagacaaactgtcaattatgagta

1441 tttatttaaattttaactaattattgtcattaaaagtgtcactgtagaagatgaactaat

1501 tgatctagttgacaaacaaatatattaatgctaggtctttctgcccttgctagttttttt

1561 tttctttttttttttctccaaagtccttgtttgagaagactgcggtatatagcgctattt

1621 ttctattaaccgtattggttcgtgtgattttgcctccaaaattcgtttctcaattaattc

1681 cgatcgcaactttagttttgacgacatgcgtttcagaagtaagtcatatcggtgaggcta

1741 gtccatgtactgtactacgcaagactagtttatctgaacaagtattacttgggtgtaaga

1801 cagtcactaattaaaggaaacaaaatttgcctgtaaaaaaacaagtctttgtcttggtgt

1861 aaatttcggatgcgtttgggcaagattcagttttaaaaattcacattttagtggcgtcag

1921 tgaagacacgttgatgtaccagctagtcttcaaatgttggtaatagaaacaatccattcc

1981 caaagttcgttttgtcgactaaaaaaccaccaatgtcgagaaaaacccaaatcatttcgt

2041 tcaaaccttagatagcaataaatagtttaattttgacacaagagctggccaaagccgcca

2101 ttttgcagtcgataaccagaacaacagcccattccaatccgaacaaattaattattaaat

2161 atatttgtctgctttcagcttgtacatttctttaaagcgattaatgataacttaatgcag

2221 agtattgaacattggagagttttaaaattgtagatgctcctcttagcttaactcaagaaa

2281 ggttacgtagtagcactctagtgtcaaaggtcatgctgtatccacactgacacgcccccc

2341 cccccccacacacaaccacaaaccatgcccttctcccataactgaacgggcatactcgat

2401 atcgggtacaacaattatgaaaaccatgttttgaagaagcgctgaagtcttaatgcagac

2461 tttactatcaatctttatgcatattgacggacgggtgtctgctaagatgacaaacaaaat

2521 tcactcaccacagcgaaataaataatgaaccaaattttgatgggagggatttgccttccg

2581 aacaatctccagaatctgccaagagggtgctactgcctatactgggaagagtgaccgatt

2641 accgtaggccctctaccgttttgctgacgagacaaattgtataaagataaagaagaacat

2701 attttgtgtcttgattttttaagtcatgttattaaatctagtgagtaaattagcccacgta

2761 ccgtctggcaccctgactcaacgctgaactaccgatagtgttatcgtaatgtgcattttt

2821 gaaaaacttcttcaattacccctgttaaatgtatagtttaaagttaataacttgtgttca

2881 aacattaatgagagttagccccagggtccatgcgcaaattacgcaaacgtaaacgtaaac

2941 atgttacgttgtttcggatgagacaaattcacaagctcatgtttacgtttcgcgtaccta

3001 gcttaatagcatagagaaagcccagtggtcctttatctatgctctgtaagctatagtcca

3061 ctctcactctgtctcggggctattaagtgttacttcctactgctatacatcacccactgt

3121 ttgcctatgtctaggttggacatcaaaaacaacatgtagtcacggaataaacatggagtt

3181 ccaggtaatacgcaaacatgttacctgaggaattgatgatgtagcgatactgggccttat

3241 cattgactgatcgtaatggcgctgtctatggtaattataagtatacatccgataattggt

3301 tgataatactccattaggtagaccccccccccccccccccccc

Fig. S3. *A. rubens* BN-type neuropeptide precursor ArBNP. The nucleotide sequence (lowercase, numbered on the left) encoding ArBNP (uppercase, numbered on the right) is shown. The predicted signal peptide is highlighted in blue, the ArBN neuropeptide sequence is highlighted in red, C-terminal glycine (G) residue that is a predicted substrate for amidation is shown in orange and a putative dibasic cleavage site is highlighted in green. The asterisk shows the position of the stop codon. This sequence has been deposited in GenBank under accession number XM_033768834.1. A cDNA containing the coding region was cloned by PCR using specific primers (underlined) and sequenced.

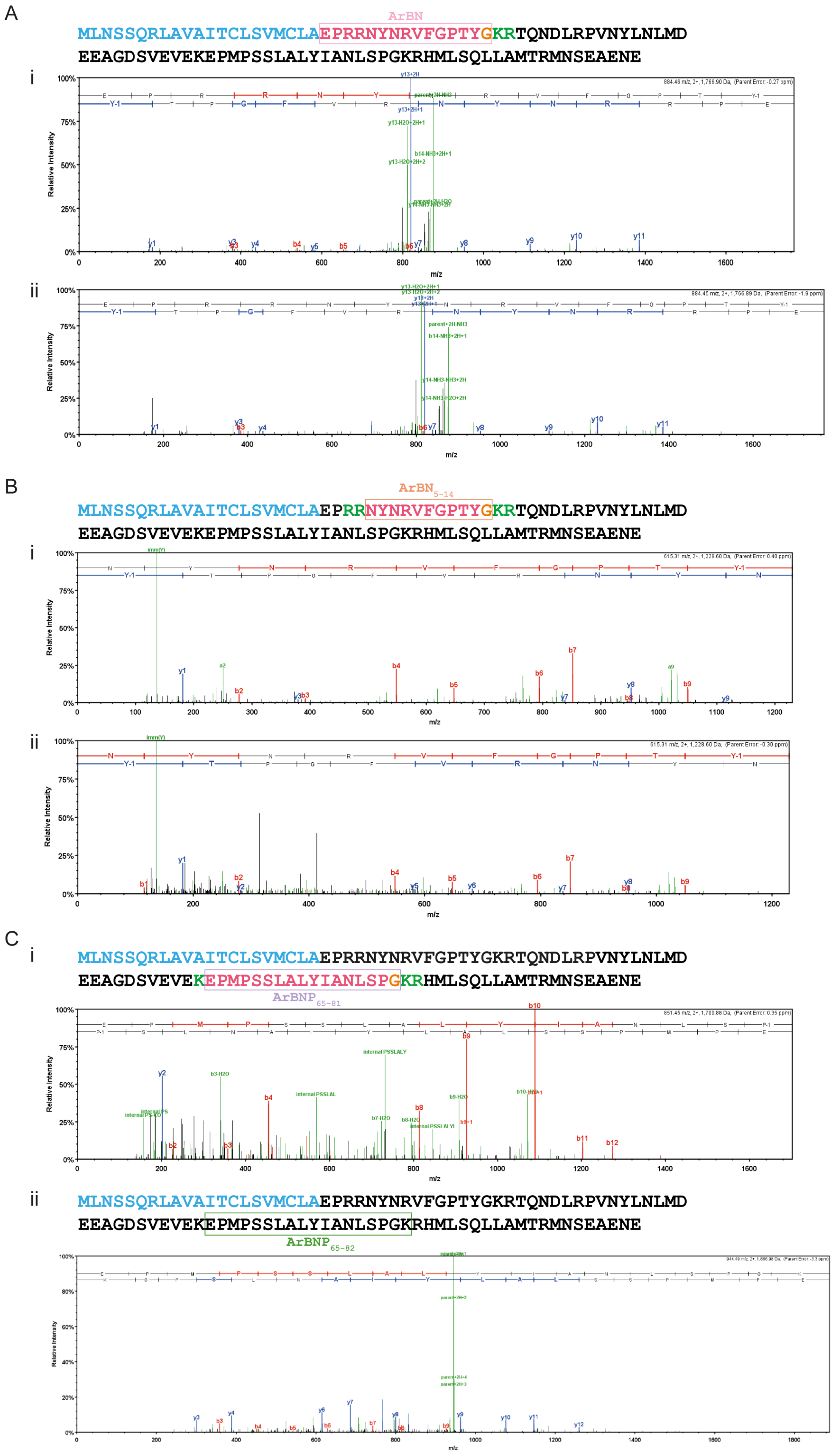
A
ArBN
MLNSSQRLAVAITCLSVMCLAEPRRNYNRVFGPTYGKRTQNDLRPVNYLNLMD
EEAGDSVEVEKEPMPSSLALYIANLSPGKRHMLSQLLAMTRMNSEAENE
i
Relative Intensity
m/z
ii
Relative Intensity
m/z
B
ArBN5-14
MLNSSQRLAVAITCLSVMCLAEPRRNYNRVFGPTYGKRTQNDLRPVNYLNLMD
EEAGDSVEVEKEPMPSSLALYIANLSPGKRHMLSQLLAMTRMNSEAENE
i
Relative Intensity
m/z
ii
Relative Intensity
m/z
C
i
MLNSSQRLAVAITCLSVMCLAEPRRNYNRVFGPTYGKRTQNDLRPVNYLNLMD
EEAGDSVEVEKEPMPSSLALYIANLSPGKRHMLSQLLAMTRMNSEAENE
ArBNP65-81
Relative Intensity
m/z
ii
MLNSSQRLAVAITCLSVMCLAEPRRNYNRVFGPTYGKRTQNDLRPVNYLNLMD
EEAGDSVEVEKEPMPSSLALYIANLSPGKRHMLSQLLAMTRMNSEAENE
ArBNP65-82
Relative Intensity
m/z

Fig. S4. Mass spectrometric characterization of neuropeptides derived from ArBN precursor. (A) Amino acid sequence of ArBN precursor is shown at the top. The predicted signal peptide is shown in blue, the predicted cleavage site is shown in green and the predicted ArBN peptide is shown in red but with the C-terminal glycine, which is a predicted substrate for amidation, shown in orange. The part of the precursor containing the ArBN peptide detected in an *A. rubens* radial nerve cord extract by mass spectrometry is also highlighted with a pink rectangle. Mass spectra derived from MS/MS analysis of (i) the synthetic peptide ArBN (EPRRNYNRVFGPTY-NH_2; 884.45 m/z, 2+ ion); and (ii) the same peptide detected in an extract of radial nerve cords from *A. rubens*. (B) Amino acid sequence of ArBN precursor is shown at the top. The predicted signal peptide is shown in blue, predicted cleavage sites are shown in green and the predicted peptide $ArBN_{5-14}$ is shown in red but with the C-terminal glycine, which is a predicted substrate for the amidation, shown in orange. The part of the precursor containing the $ArBN_{5-14}$ peptide detected an in *A. rubens* radial nerve cord extract by the mass spectrometry is also highlighted with an orange rectangle. Mass spectra derived from MS/MS analysis of (i) the synthetic peptide $ArBN_{5-14}$ (NYNRVFGPTY-NH_2; 615.31 m/z, 2+ ion); and (ii) the same peptide detected in an extract of radial nerve cords from *A. rubens*. (C) (i) Amino acid sequence of ArBN precursor is shown at the top. The predicted signal peptide is shown in blue, predicted cleavage sites are shown in green and the predicted peptide $ArBNP_{65-81}$ is shown in red but with the C-terminal glycine, which is a predicted substrate for the amidation, shown in orange. The $ArBNP_{65-81}$ peptide is highlighted with a purple rectangle. Below the precursor sequence is a mass spectrum derived from MS/MS analysis of the synthetic peptide $ArBNP_{65-81}$ (EPMPSSLALYIANLSP-NH_2; 851.45 m/z, 2+ ion). (ii) Amino acid sequence of ArBN precursor is shown at the top. The predicted signal peptide is shown in blue. The $ArBNP_{65-82}$ peptide that was detected in an extract of *A. rubens* radial nerve cords by mass spectrometry is highlighted with a green rectangle. Below the precursor sequence is a mass spectrum showing that the $ArBNP_{65-82}$ peptide (EPMPSSLALYIANLSPGK; 944.49 m/z, 2+ ion) is detected in an extract of radial nerve cords from *A. rubens* that had been subjected to trypsin digestion.

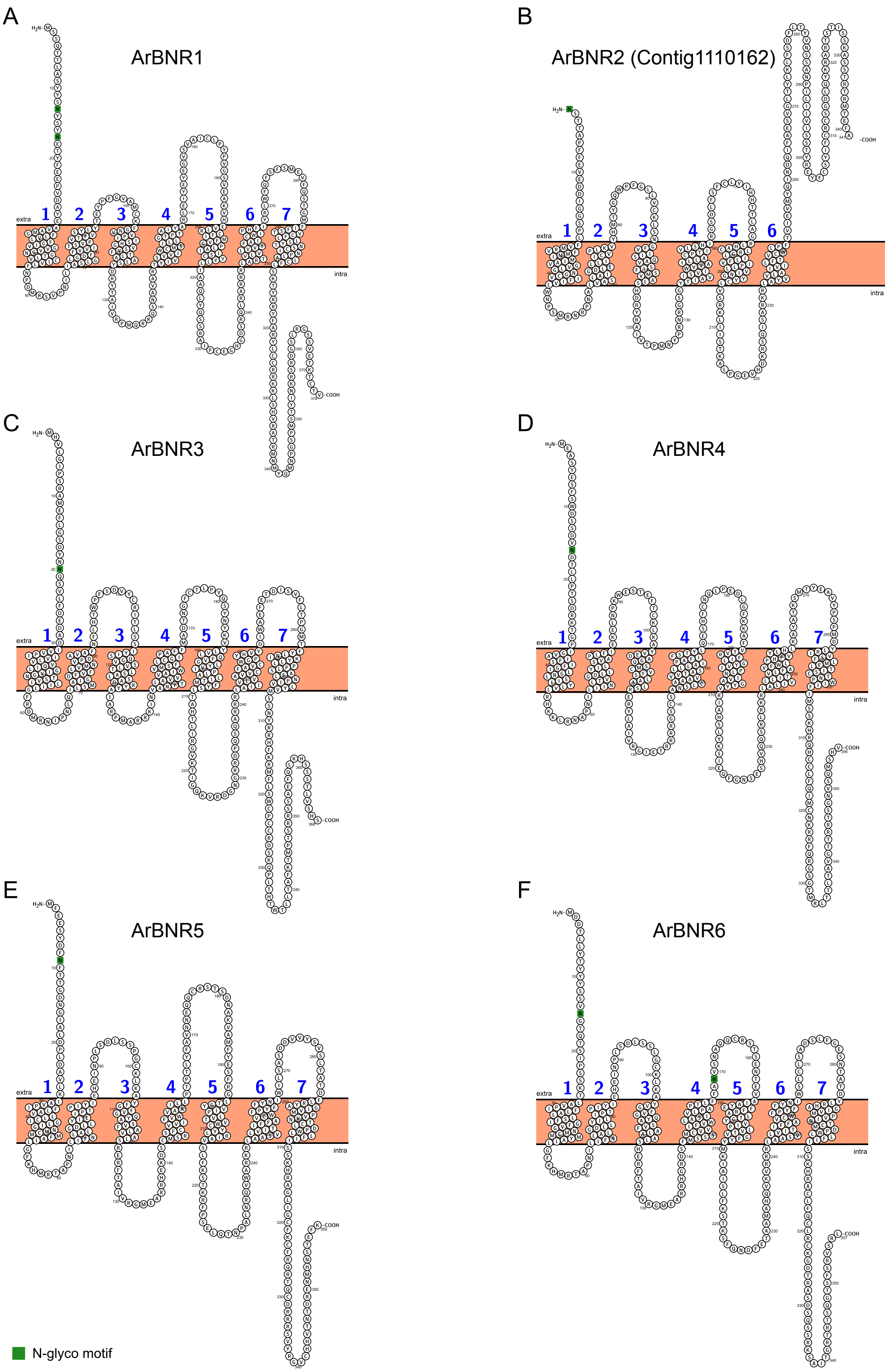
A
ArBNR1
B
ArBNR2 (Contig1110162)
C
ArBNR3
D
ArBNR4
E
ArBNR5
F
ArBNR6
extra
intra
N-glyco motif

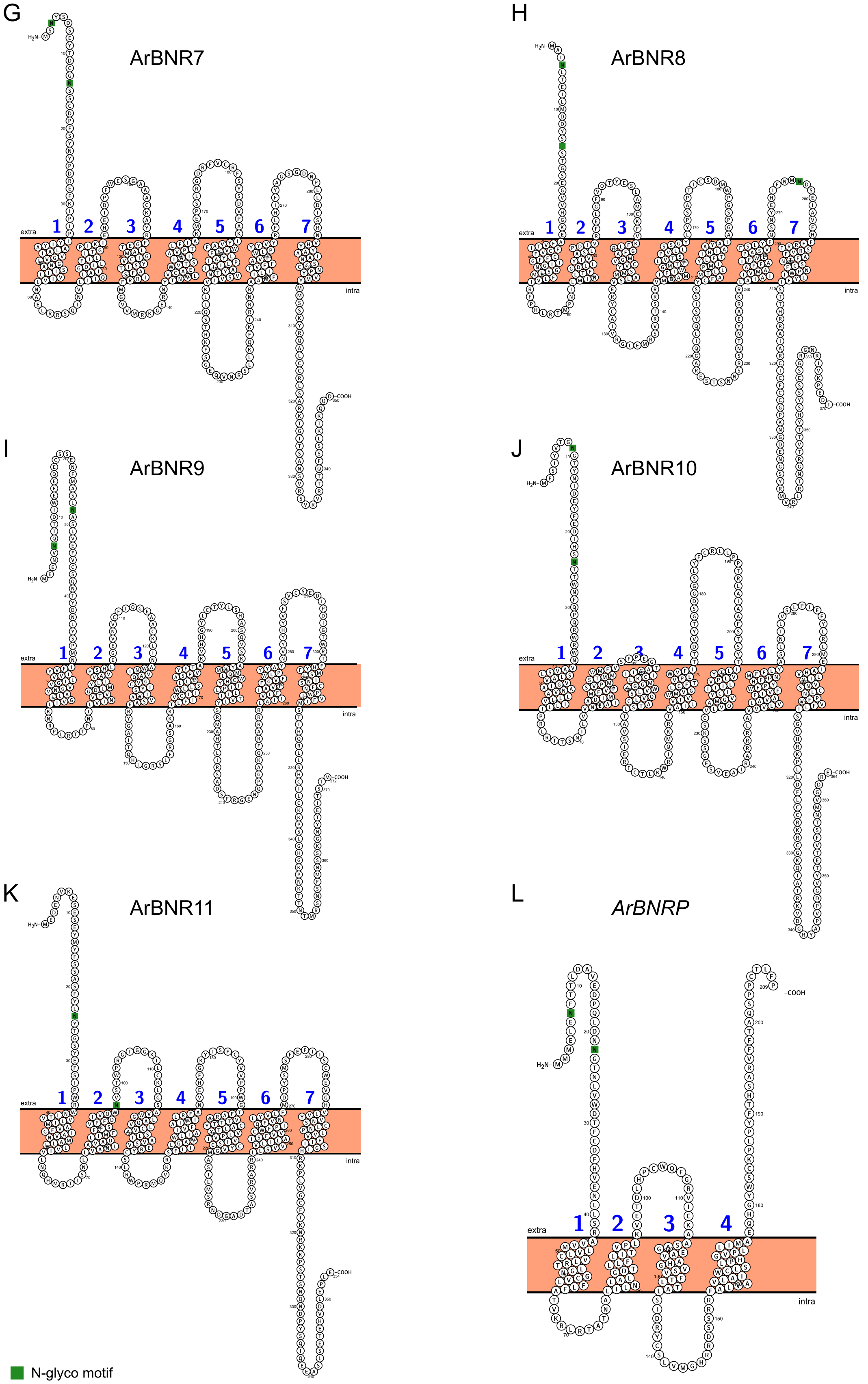
G
ArBNR7
H
ArBNR8
I
ArBNR9
J
ArBNR10
K
ArBNR11
L
ArBNRP
extra
intra
N-glyco motif

Fig. S5. Predicted topologies of ArBNR1-11 and *ArBNRP*. ArBNR1 and ArBNR3-ArBNR11 contain seven predicted transmembrane domains, as expected for GPCRs. ArBNR2 (a 341-residue protein encoded by contig 1110162) contains six predicted transmembrane domains. *ArBNRP* contains four predicted transmembrane domains and, as explained above, *ArBNRP* has been identified as pseudogene. The predicted transmembrane domains are numbered successively in blue and predicted N-glycosylation sites are highlighted in green. These diagrams were generated using Protter v1.0 (http://wlab.ethz.ch/protter/start/).

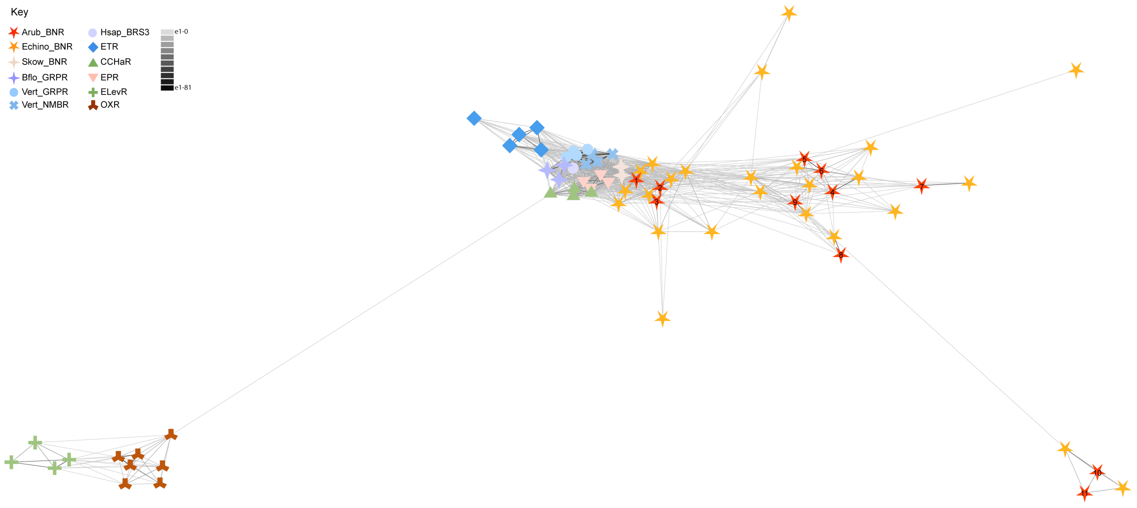

Fig. S6. CLANS analysis of BN/ET/CCHa/EP-type receptors. The label of each node is shown in the key. Links represent BLAST relationships with a P value >1e-40. Abbreviations: Arub_BNR (*Asterias rubens* bombesin-type receptor), Echino_BNR (Echinoderm bombesin-type receptor), Skow_BNR (*Saccoglossus kowalevskii* bombesin-type receptor), Bflo_GRPR (*Branchiostoma floridae* Gastrin-releasing peptide-type receptor), Hsap_BRS3 (*Homo sapiens* bombesin receptor subtype 3), Vert_GRPR (Vertebrate Gastrin-releasing peptide receptor), Vert_NMBR (Vertebrate Neuromedin B receptor), ETR (Endothelin receptor), CCHaR (CCHamide peptide receptor), EPR (Excitatory peptide receptor), ELevR (Elevenin receptor), OXR (Orexin receptor). The sequences of the receptors included in this figure are listed in *SI Appendix,* Table S4.

A

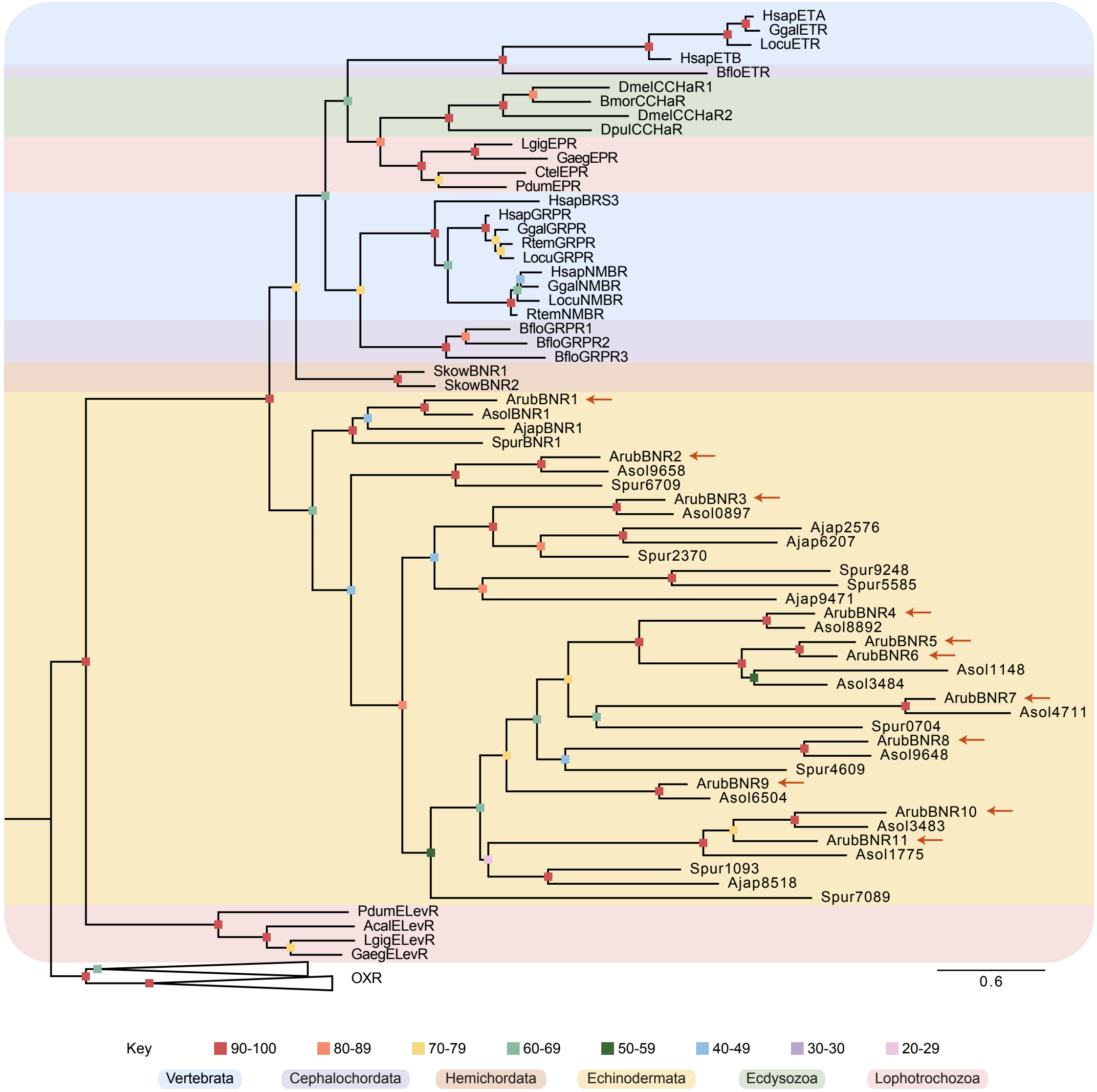
HsapETA
GgalETR
LocuETR
HsapETB
BfloETR
DmelCCHaR1
BmorCCHaR
DmelCCHaR2
DpulCCHaR
LgigEPR
GaegEPR
CtelEPR
PdumEPR
HsapBRS3
HsapGRPR
GgalGRPR
RtemGRPR
LocuGRPR
HsapNMBR
GgalNMBR
LocuNMBR
RtemNMBR
BfloGRPR1
BfloGRPR2
BfloGRPR3
SkowBNR1
SkowBNR2
ArubBNR1
AsolBNR1
AjapBNR1
SpurBNR1
ArubBNR2
Asol9658
Spur6709
ArubBNR3
Asol0897
Ajap2576
Ajap6207
Spur2370
Spur9248
Spur5585
Ajap9471
ArubBNR4
Asol8892
ArubBNR5
ArubBNR6
Asol1148
Asol3484
ArubBNR7
Asol4711
Spur0704
ArubBNR8
Asol9648
Spur4609
ArubBNR9
Asol6504
ArubBNR10
Asol3483
ArubBNR11
Asol1775
Spur1093
Ajap8518
Spur7089
PdumELevR
AcalELevR
LgigELevR
GaegELevR
OXR
0.6
Key
90-100
80-89
70-79
60-69
50-59
40-49
30-30
20-29
Vertebrata
Cephalochordata
Hemichordata
Echinodermata
Ecdysozoa
Lophotrochozoa

B

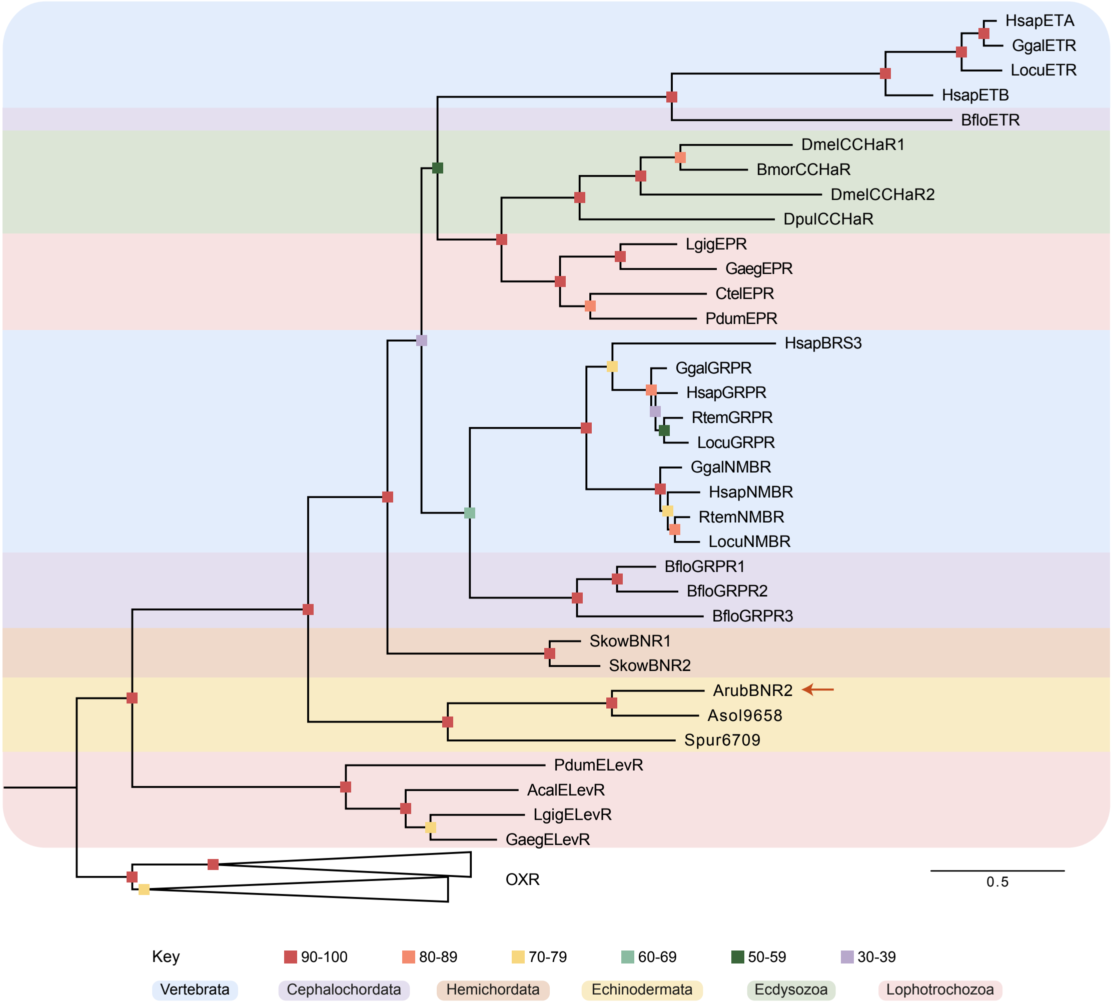

HsapETA
GgalETR
LocuETR
HsapETB
BfloETR
DmelCCHaR1
BmorCCHaR
DmelCCHaR2
DpulCCHaR
LgigEPR
GaegEPR
CtelEPR
PdumEPR
HsapBRS3
GgalGRPR
HsapGRPR
RtemGRPR
LocuGRPR
GgalNMBR
HsapNMBR
RtemNMBR
LocuNMBR
BfloGRPR1
BfloGRPR2
BfloGRPR3
SkowBNR1
SkowBNR2
ArubBNR2
Asol9658
Spur6709
PdumELevR
AcalELevR
LgigELevR
GaegELevR
OXR
0.5
Key
90-100
80-89
70-79
60-69
50-59
30-39
Vertebrata
Cephalochordata
Hemichordata
Echinodermata
Ecdysozoa
Lophotrochozoa

C

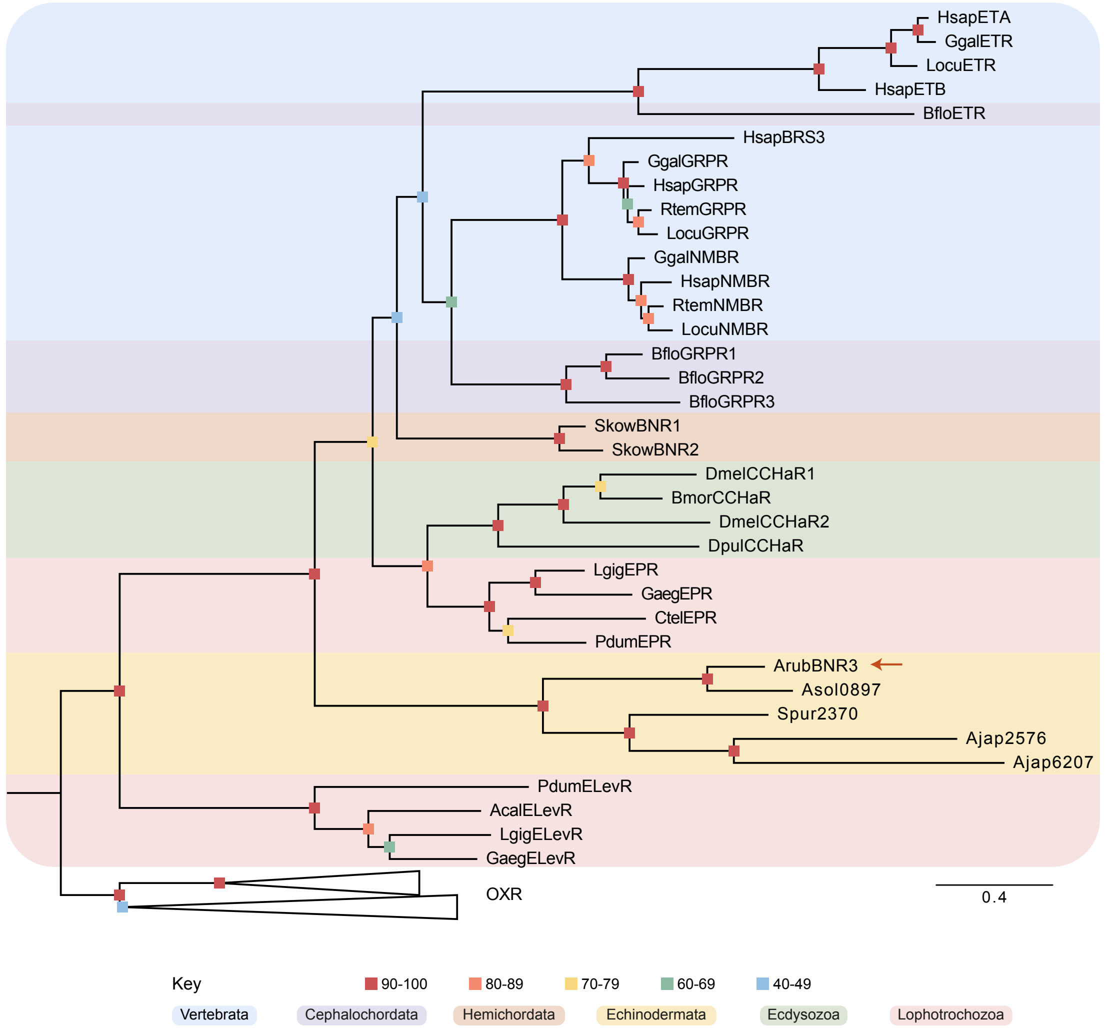
HsapETA
GgalETR
LocuETR
HsapETB
BfloETR
HsapBRS3
GgalGRPR
HsapGRPR
RtemGRPR
LocuGRPR
GgalNMBR
HsapNMBR
RtemNMBR
LocuNMBR
BfloGRPR1
BfloGRPR2
BfloGRPR3
SkowBNR1
SkowBNR2
DmelCCHaR1
BmorCCHaR
DmelCCHaR2
DpulCCHaR
LgigEPR
GaegEPR
CtelEPR
PdumEPR
ArubBNR3
Asol0897
Spur2370
Ajap2576
Ajap6207
PdumELevR
AcalELevR
LgigELevR
GaegELevR
OXR
0.4
Key
90-100
80-89
70-79
60-69
40-49
Vertebrata
Cephalochordata
Hemichordata
Echinodermata
Ecdysozoa
Lophotrochozoa

D

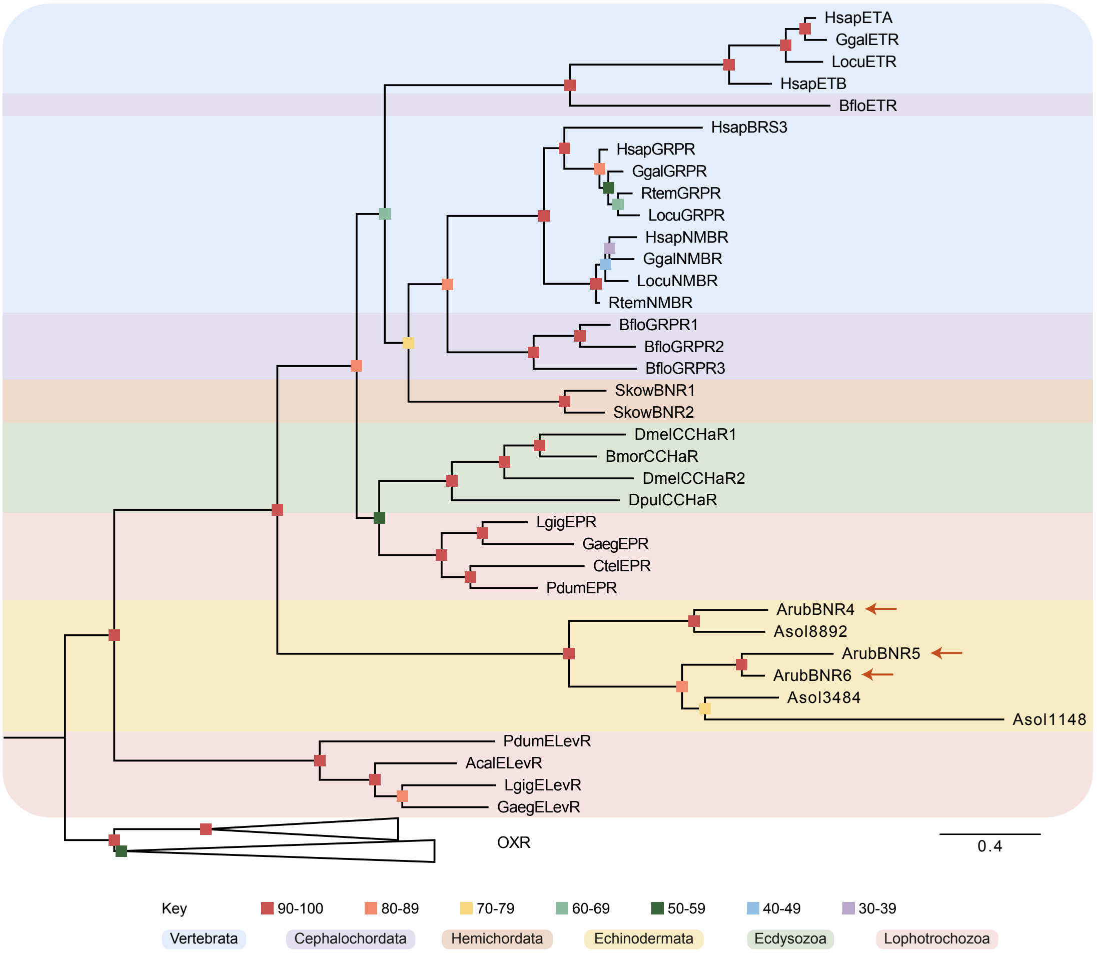

HsapETA
GgalETR
LocuETR
HsapETB
BfloETR
HsapBRS3
HsapGRPR
GgalGRPR
RtemGRPR
LocuGRPR
HsapNMBR
GgalNMBR
LocuNMBR
RtemNMBR
BfloGRPR1
BfloGRPR2
BfloGRPR3
SkowBNR1
SkowBNR2
DmelCCHaR1
BmorCCHaR
DmelCCHaR2
DpulCCHaR
LgigEPR
GaegEPR
CtelEPR
PdumEPR
ArubBNR4
Asol8892
ArubBNR5
ArubBNR6
Asol3484
Asol1148
PdumELevR
AcalELevR
LgigELevR
GaegELevR
OXR
0.4
Key
90-100
80-89
70-79
60-69
50-59
40-49
30-39
Vertebrata
Cephalochordata
Hemichordata
Echinodermata
Ecdysozoa
Lophotrochozoa

E

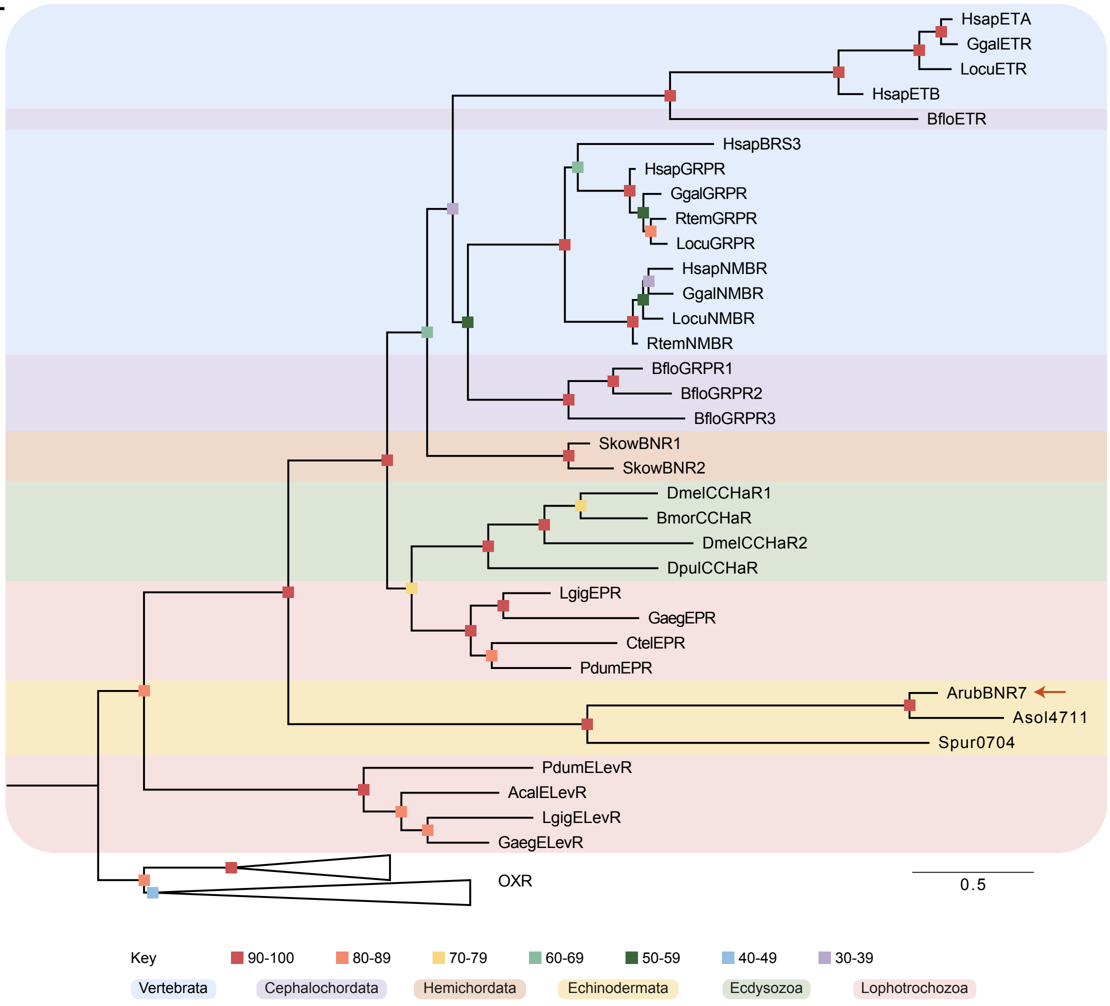

HsapETA
GgalETR
LocuETR
HsapETB
BfloETR
HsapBRS3
HsapGRPR
GgalGRPR
RtemGRPR
LocuGRPR
HsapNMBR
GgalNMBR
LocuNMBR
RtemNMBR
BfloGRPR1
BfloGRPR2
BfloGRPR3
SkowBNR1
SkowBNR2
DmelCCHaR1
BmorCCHaR
DmelCCHaR2
DpulCCHaR
LgigEPR
GaegEPR
CtelEPR
PdumEPR
ArubBNR7
Asol4711
Spur0704
PdumELevR
AcalELevR
LgigELevR
GaegELevR
OXR
0.5
Key
90-100
80-89
70-79
60-69
50-59
40-49
30-39
Vertebrata
Cephalochordata
Hemichordata
Echinodermata
Ecdysozoa
Lophotrochozoa

F

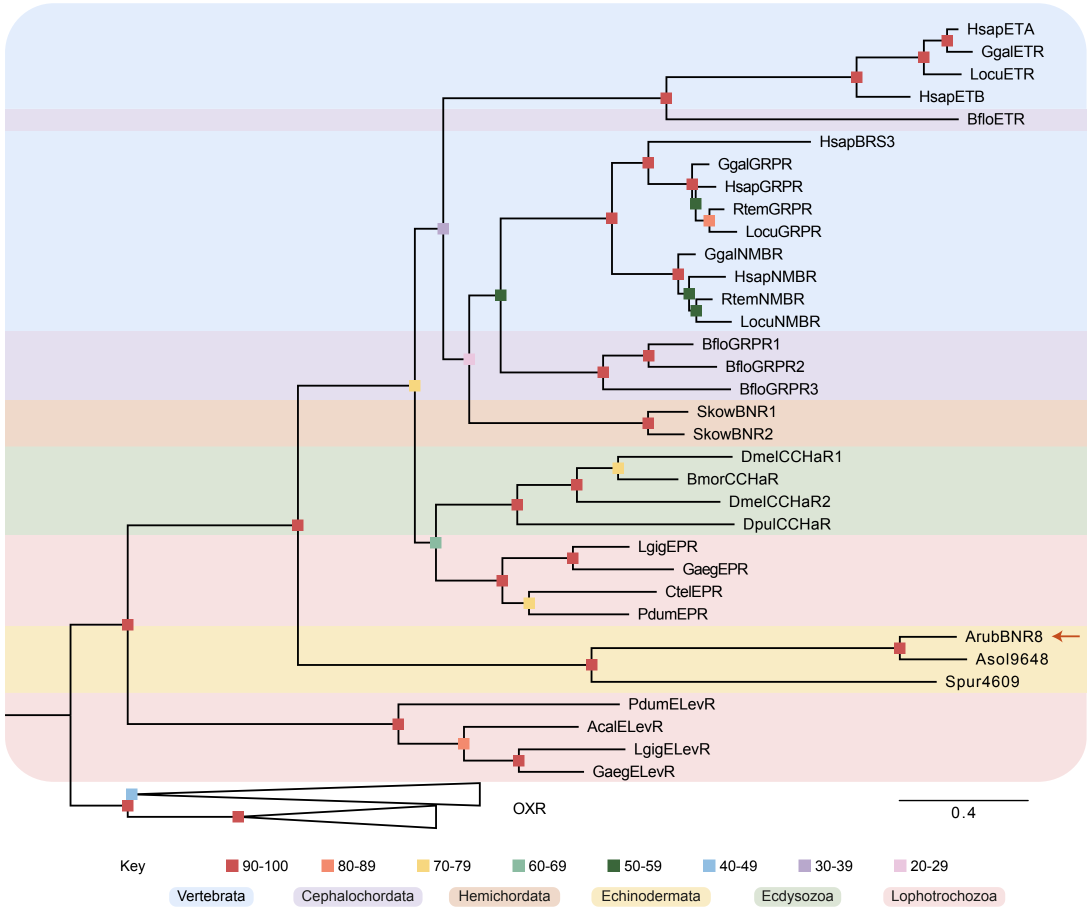
HsapETA
GgalETR
LocuETR
HsapETB
BfloETR
HsapBRS3
GgalGRPR
HsapGRPR
RtemGRPR
LocuGRPR
GgalNMBR
HsapNMBR
RtemNMBR
LocuNMBR
BfloGRPR1
BfloGRPR2
BfloGRPR3
SkowBNR1
SkowBNR2
DmelCCHaR1
BmorCCHaR
DmelCCHaR2
DpulCCHaR
LgigEPR
GaegEPR
CtelEPR
PdumEPR
ArubBNR8
Asol9648
Spur4609
PdumELevR
AcalELevR
LgigELevR
GaegELevR
OXR
0.4
Key
90-100
80-89
70-79
60-69
50-59
40-49
30-39
20-29
Vertebrata
Cephalochordata
Hemichordata
Echinodermata
Ecdysozoa
Lophotrochozoa

G

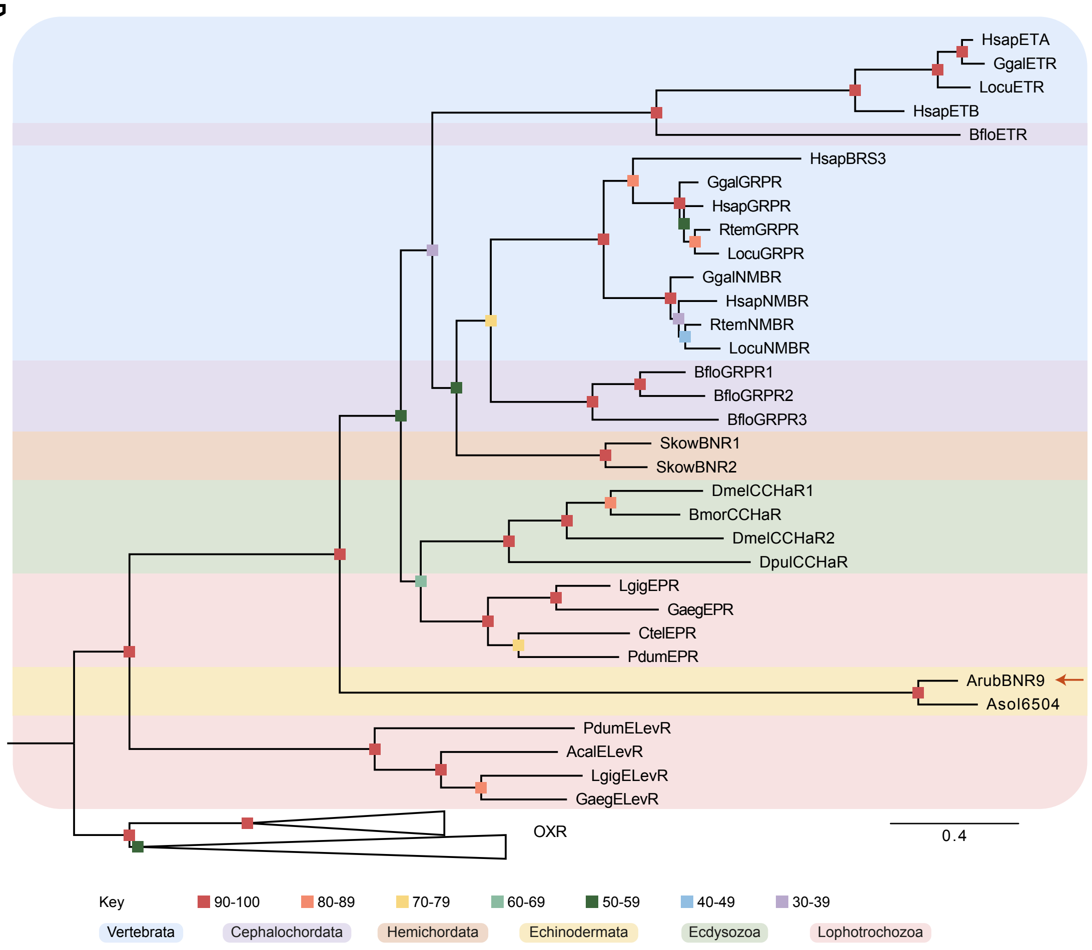
HsapETA
GgalETR
LocuETR
HsapETB
BfloETR
HsapBRS3
GgalGRPR
HsapGRPR
RtemGRPR
LocuGRPR
GgalNMBR
HsapNMBR
RtemNMBR
LocuNMBR
BfloGRPR1
BfloGRPR2
BfloGRPR3
SkowBNR1
SkowBNR2
DmelCCHaR1
BmorCCHaR
DmelCCHaR2
DpulCCHaR
LgigEPR
GaegEPR
CtelEPR
PdumEPR
ArubBNR9
Asol6504
PdumELevR
AcalELevR
LgigELevR
GaegELevR
OXR
0.4
Key
90-100
80-89
70-79
60-69
50-59
40-49
30-39
Vertebrata
Cephalochordata
Hemichordata
Echinodermata
Ecdysozoa
Lophotrochozoa

H

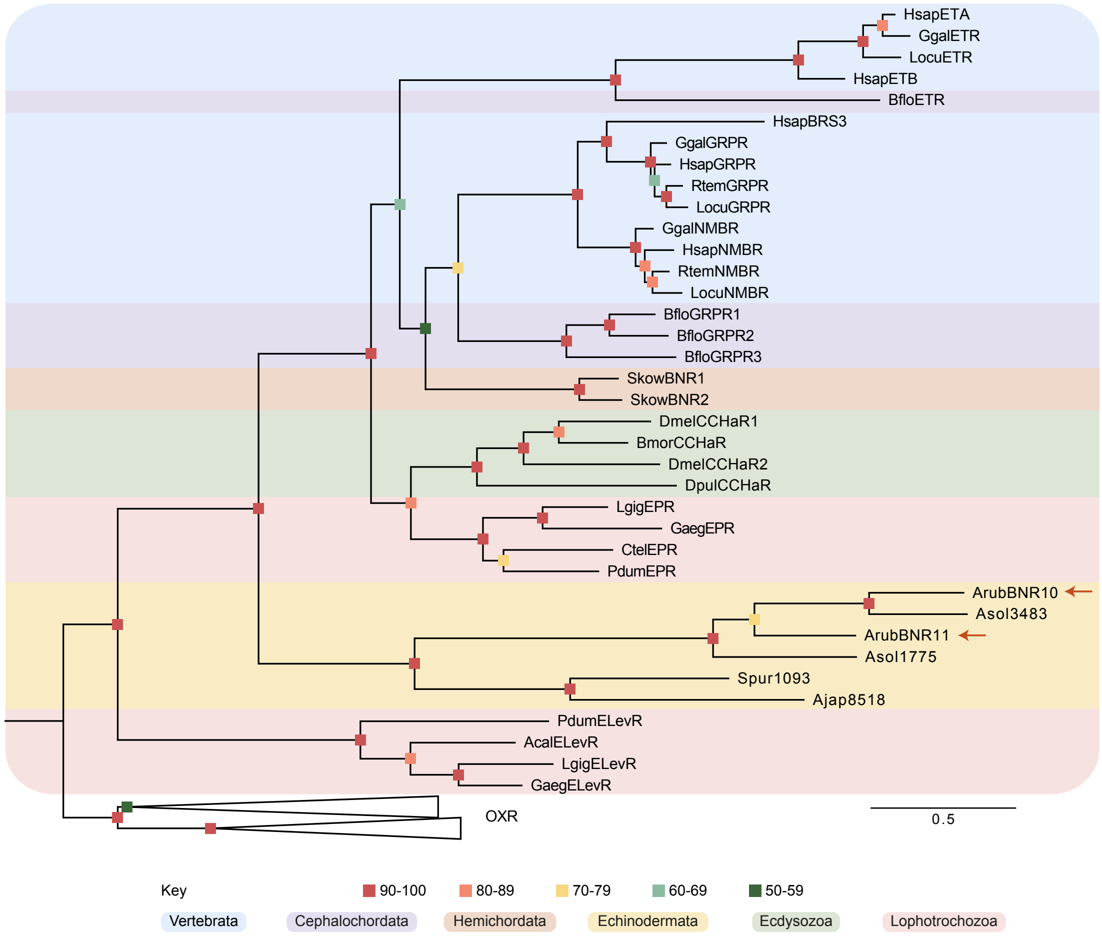

HsapETA
GgalETR
LocuETR
HsapETB
BfloETR
HsapBRS3
GgalGRPR
HsapGRPR
RtemGRPR
LocuGRPR
GgalNMBR
HsapNMBR
RtemNMBR
LocuNMBR
BfloGRPR1
BfloGRPR2
BfloGRPR3
SkowBNR1
SkowBNR2
DmelCCHaR1
BmorCCHaR
DmelCCHaR2
DpulCCHaR
LgigEPR
GaegEPR
CtelEPR
PdumEPR
ArubBNR10
Asol3483
ArubBNR11
Asol1775
Spur1093
Ajap8518
PdumELevR
AcalELevR
LgigELevR
GaegELevR
OXR
0.5
Key
90-100
80-89
70-79
60-69
50-59
Vertebrata
Cephalochordata
Hemichordata
Echinodermata
Ecdysozoa
Lophotrochozoa

Fig. S7. Phylogenetic analysis of all candidate *A. rubens* bombesin-type receptors (ArBNR1-11) and individual receptors (ArBNR2-11 individually) with GRP/NMB/BN-type, ET-type, CCHa/EP-type and elevenin (ELev)-type receptors from other taxa. Trees were constructed using the maximum likelihood method (1000 bootstrap replicates) with orexin-type receptors as an outgroup to root the tree. (A) All *A. rubens* bombesin-type receptors (ArBNR1-11) (arrows) and other echinoderm BN-type receptors (LG+F+I+G4 model). (B) ArBNR2 (arrow) and related echinoderm BN-type receptors (LG+F+G4 model). (C) ArBNR3 (arrow) and related echinoderm BN-type receptors (LG+F+G4 model). (D) ArBNR4, 5, 6 (arrows) and related echinoderm BN-type receptors (LG+F+G4 model). (E) ArBNR7 (arrow) and related echinoderm BN-type receptors (LG+F+G4 model). (F) ArBNR8 (arrow) and related echinoderm BN-type receptors (LG+F+I+G4 model). (G) ArBNR9 (arrow) and related echinoderm BN-type receptors (LG+F+G4 model). (H) ArBNR10, 11 (arrows) and related echinoderm BN-type receptors (LG+F+I+G4 model). The colored squares represent bootstrap support for clades and colored backgrounds highlight different taxonomic groups (see key for each tree). The scale bar represents the average residue substitution per site. Abbreviations for species names: Ajap (*Apostichopus japonicus*), Arub (*Asterias rubens*), Asol (*Acanthaster* cf. *solaris*), Bflo (*Branchiostoma floridae*), Bmor (*Bombyx mori*), Ctel (*Capitella teleta*), Dmel (*Drosophila melanogaster*), Dpul (*Daphnia pulex*), Gaeg (*Gigantopelta aegis*), Ggal (*Gallus gallus*), Hsap (*Homo sapiens*), Lgig (*Lottia gigantea*), Locu (*Lepisosteus oculatus*), Pdum (*Platynereis dumerilii*), Rtem (*Rana temporaria*), Skow (*Saccoglossus kowalevskii*), Spur (*Strongylocentrotus purpuratus*). Accession numbers of receptor sequences used to generate this figure are listed in *SI Appendix,* Table S4.

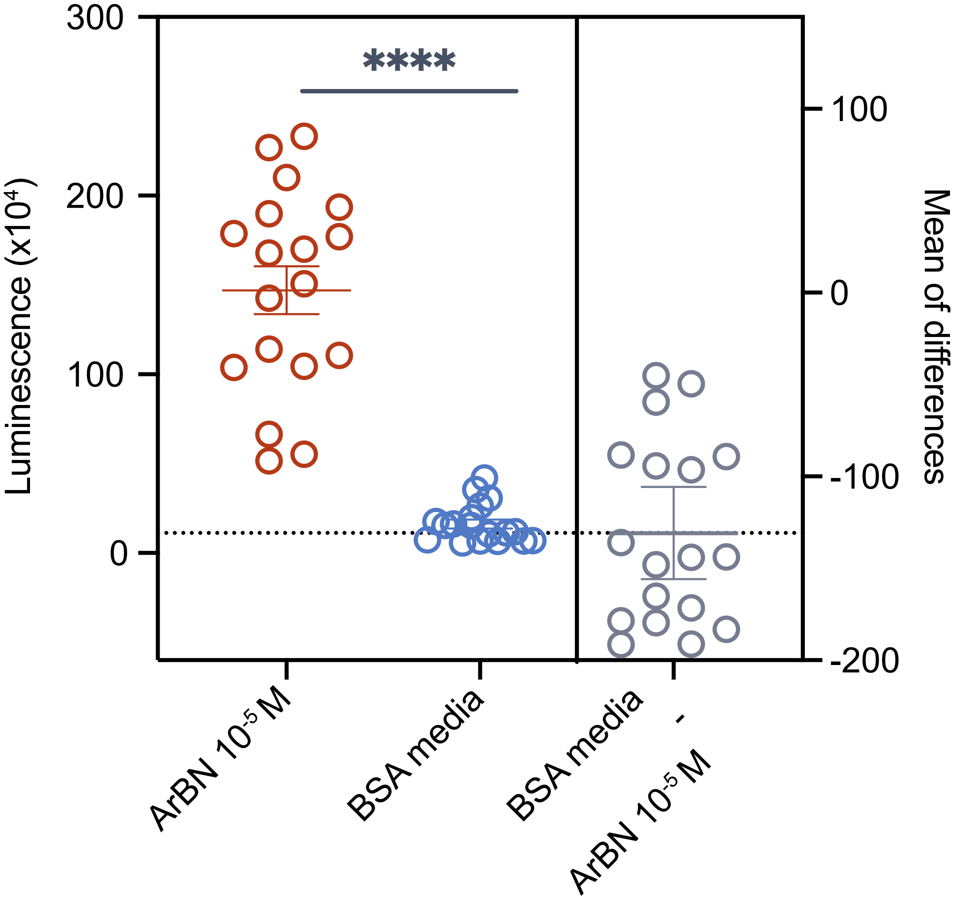

Fig. S8. Comparison of the total luminescence responses of ArBNR1-expressing cells when exposed to ArBN at a concentration of 10^{-5} M and total luminescence responses of ArBNR1-expressing cells when exposed to BSA media. The mean total luminescence response triggered by 10^{-5} M ArBN in cells expressing ArBNR1 was 147.08×10^4, which is greater than the mean total luminescence observed when cells expressing ArBNR1 were exposed to the BSA media used as a vehicle for ArBN (16.29×10^4). Data were analysed statistically using a two-tailed Student's *t*-test in Prism 10 and **** indicates $P < 0.0001$.

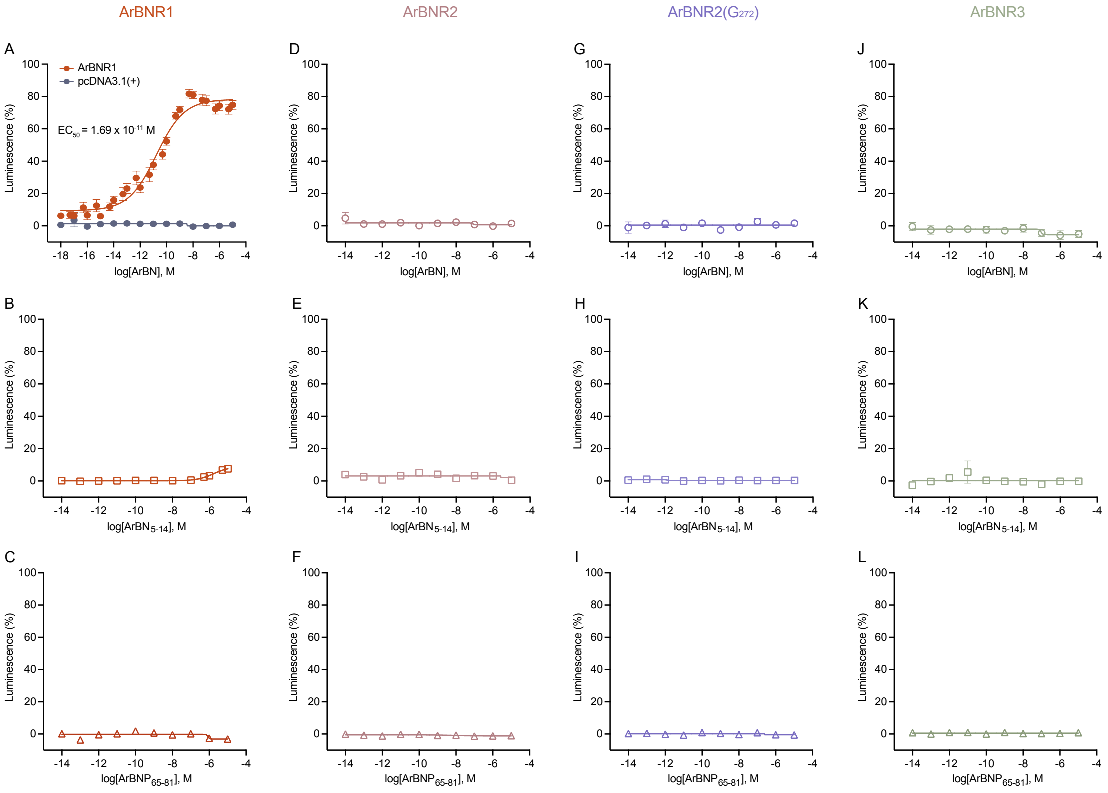

Fig. S9. Testing of ArBN, ArBN$_{5-14}$ and ArBNP$_{65-81}$ as ligands for ArBNR1-3. (A) Peptide-induced luminescence in cells expressing ArBNR1 was observed with ArBN (as also shown in Fig. 3). (B) Peptide-induced luminescence in cells expressing ArBNR1 was observed with ArBN$_{5-14,}$ but only at the highest concentrations tested (10^{-6} - 10^{-5} M). (D, G, J) ArBN did not trigger luminescence in cells expressing the other receptors tested (ArBNR2, ArBNR2(G$_{272}$) and ArBNR3). (E, H, K) ArBN$_{5-14}$ did not trigger luminescence in cells expressing the other receptors tested (ArBNR2, ArBNR2(G$_{272}$) and ArBNR3). (C, F, I, L) ArBNP$_{65-81}$ did not trigger luminescence in cells expressing ArBNR1, ArBNR2, ArBNR2(G$_{272}$) or ArBNR3.

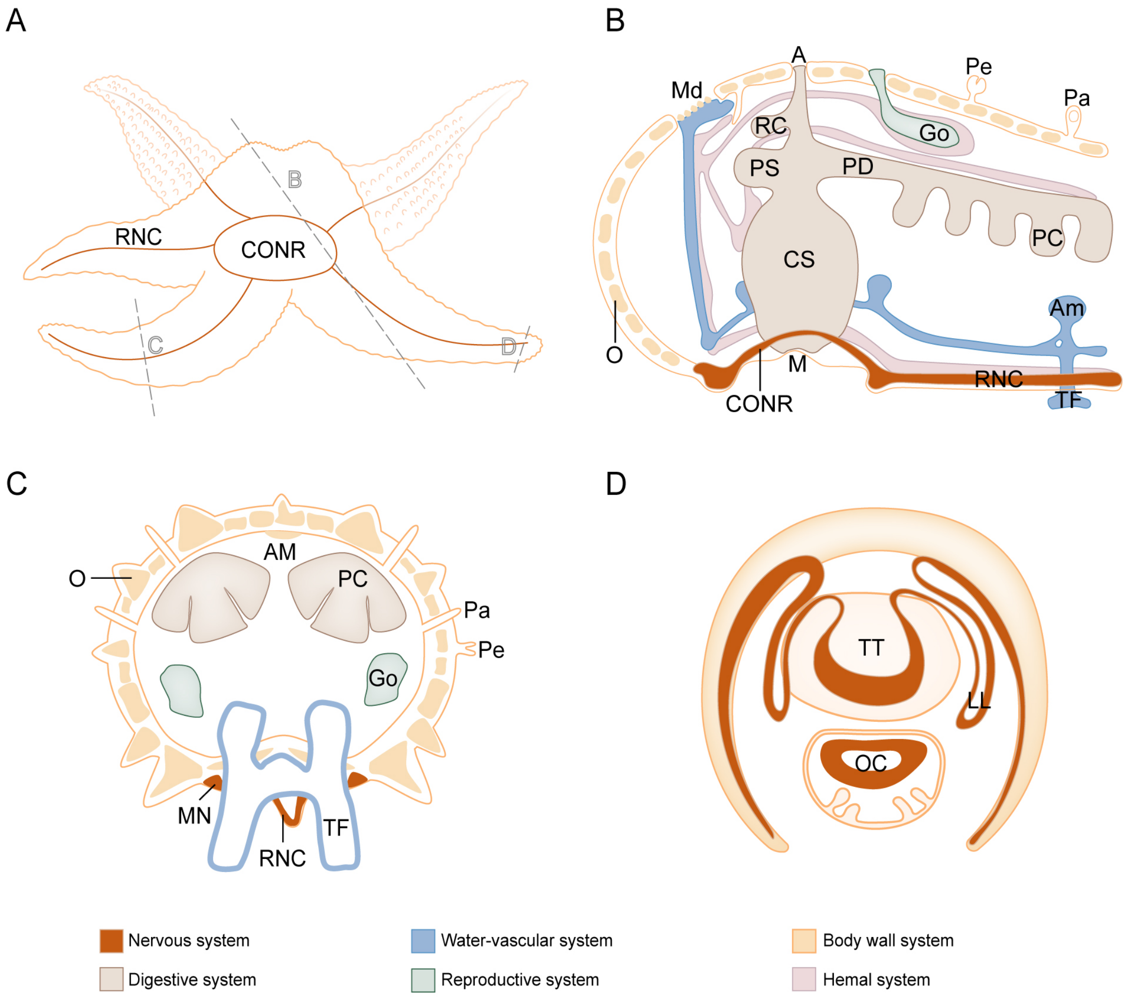

Fig. S10. Anatomy of the starfish *Asterias rubens*. (A) A schematic of a starfish with the circumoral nerve ring and five radial nerve cords shown. The grey dashed lines B, C and D show the position and orientation of the schematics in panels B, C and D, respectively. (B) A schematic showing the anatomy of the central disk region and the proximal region of one arm. (C) A schematic showing the anatomy of a starfish arm in a transverse section. (D) A schematic showing the anatomy of a starfish arm tip in a transverse section. Abbreviations: A, anus; AM, apical muscle; Amp, ampulla; CONR, circumoral nerve ring; CS, cardiac stomach; Go, gonad; LL, lateral lappet; M, mouth; Md, madreporite; MN, marginal nerve; O, ossicle; OC, optic cushion; Pa, papula; PC, pyloric caecum; PD, pyloric duct; Pe, pedicellaria; PS, pyloric stomach; RC, rectal caecum; RNC, radial nerve cord; TF, tube foot; TT, terminal tentacle.

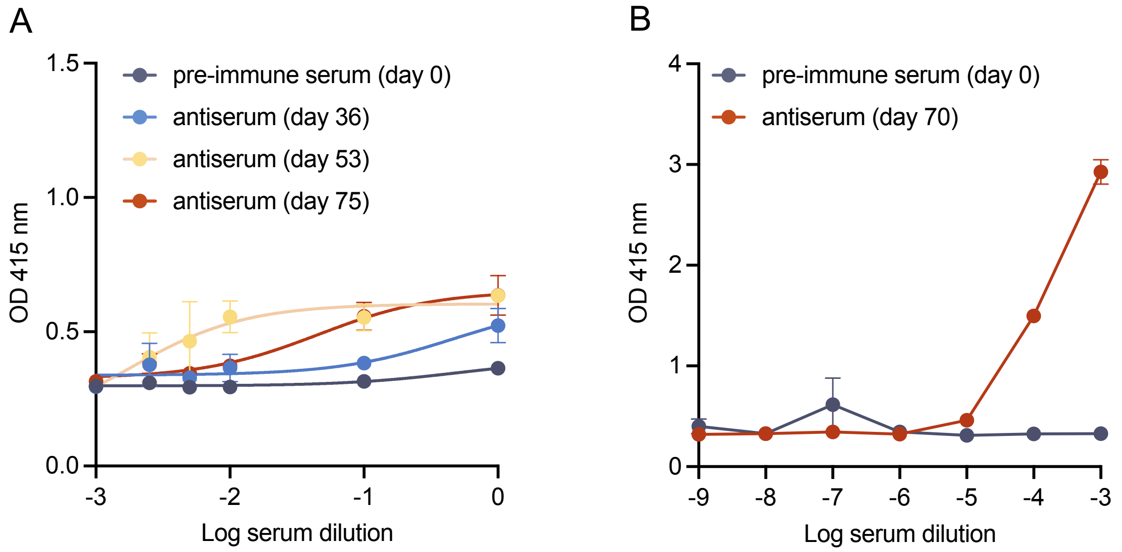

Fig. S11. Characterization of rabbit antisera to ArBNag and ArBNPag using ELISA. (A) Sera were tested for the presence of antibodies to ArBNag before, during and after immunization. Incubation of pre-immune serum (day 0, black) or antiserum (day 36, blue; day 53, yellow; day 75, red) at dilutions between 10^{-3} and 10^{-0} with 0.1 nmol of antigen peptide reveals no immunoreaction with pre-immune serum and very weak immunoreaction with undiluted antiserum and with antiserum at dilution 10^{-1}. (B) Sera were tested for the presence of antibodies to ArBNPag before and after immunization. Incubation of pre-immune serum (day 0, black) and antiserum (day 70, red) at dilutions between 10^{-9} and 10^{-3} with 0.1 nmol of antigen peptide per well reveals no immunoreaction with pre-immune serum, whereas the antigen is detected with antiserum at dilutions 10^{-3} and 10^{-4}.

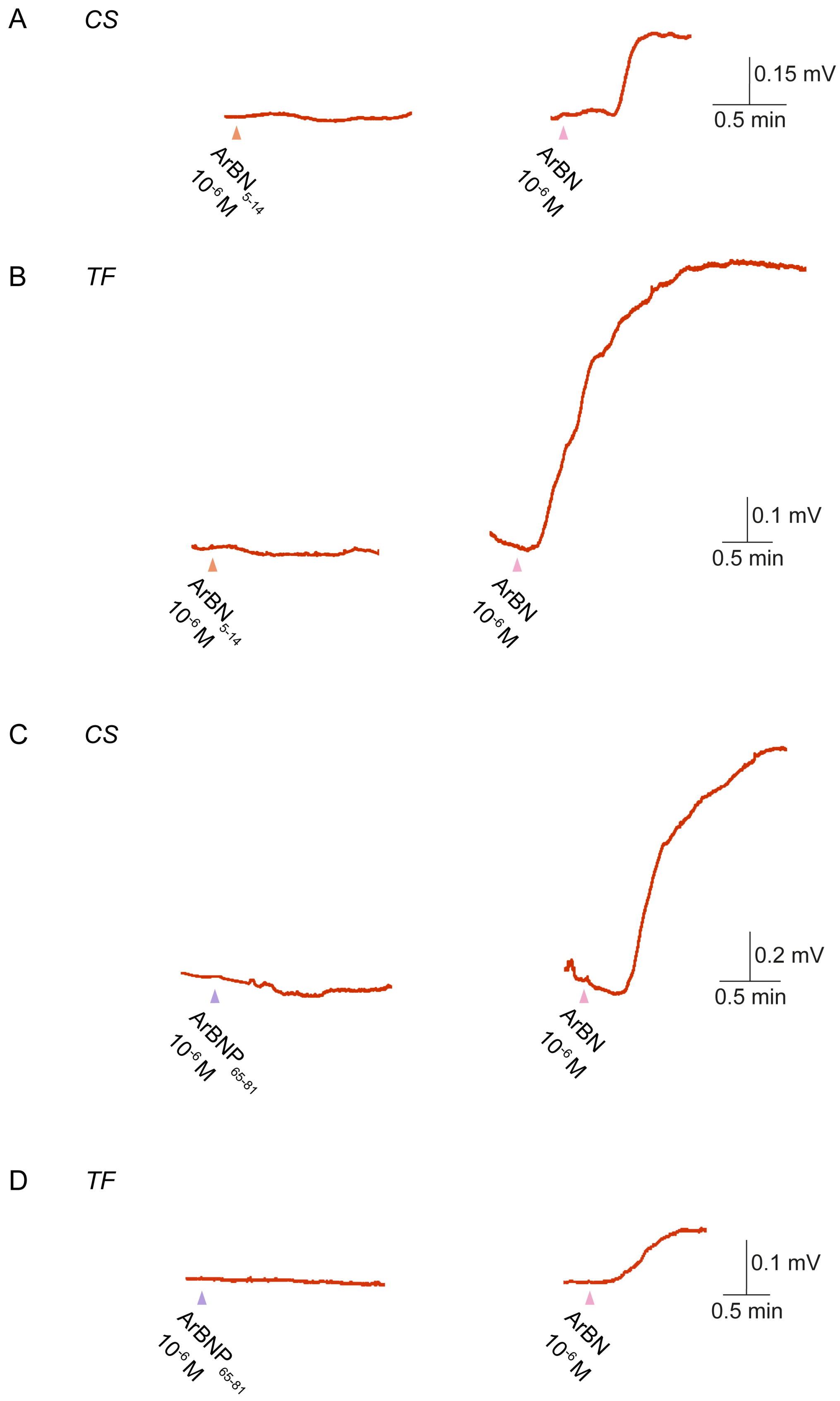
A
CS
ArBN 5-14
10-6 M
ArBN
10-6 M
0.15 mV
0.5 min
B
TF
ArBN 5-14
10-6 M
ArBN
10-6 M
0.1 mV
0.5 min
C
CS
ArBNP 65-81
10-6 M
ArBN
10-6 M
0.2 mV
0.5 min
D
TF
ArBNP 65-81
10-6 M
ArBN
10-6 M
0.1 mV
0.5 min

Fig. S12. $ArBN_{5-14}$ and $ArBNP_{65-81}$ do not have any observable effect on the contractility of cardiac stomach and tube foot preparations. (A) Representative recording showing the lack of an effect of $ArBN_{5-14}$ (10^{-6} M) on a cardiac stomach preparation, with the contracting effect of ArBN (10^{-6} M) shown as a positive control. (B) Representative recording showing the lack of an effect of $ArBN_{5-14}$ (10^{-6} M) on a tube foot preparation, with the contracting effect of ArBN (10^{-6} M) shown as a positive control. (C) Representative recording showing the lack of an effect of $ArBNP_{65-81}$ (10^{-6} M) on a cardiac stomach preparation, with the contracting effect of ArBN (10^{-6} M) shown as a positive control. (D) Representative recording showing the lack of an effect of $ArBNP_{65-81}$ (10^{-6} M) on a tube foot preparation, with the contracting effect of ArBN (10^{-6} M) shown as a positive control.

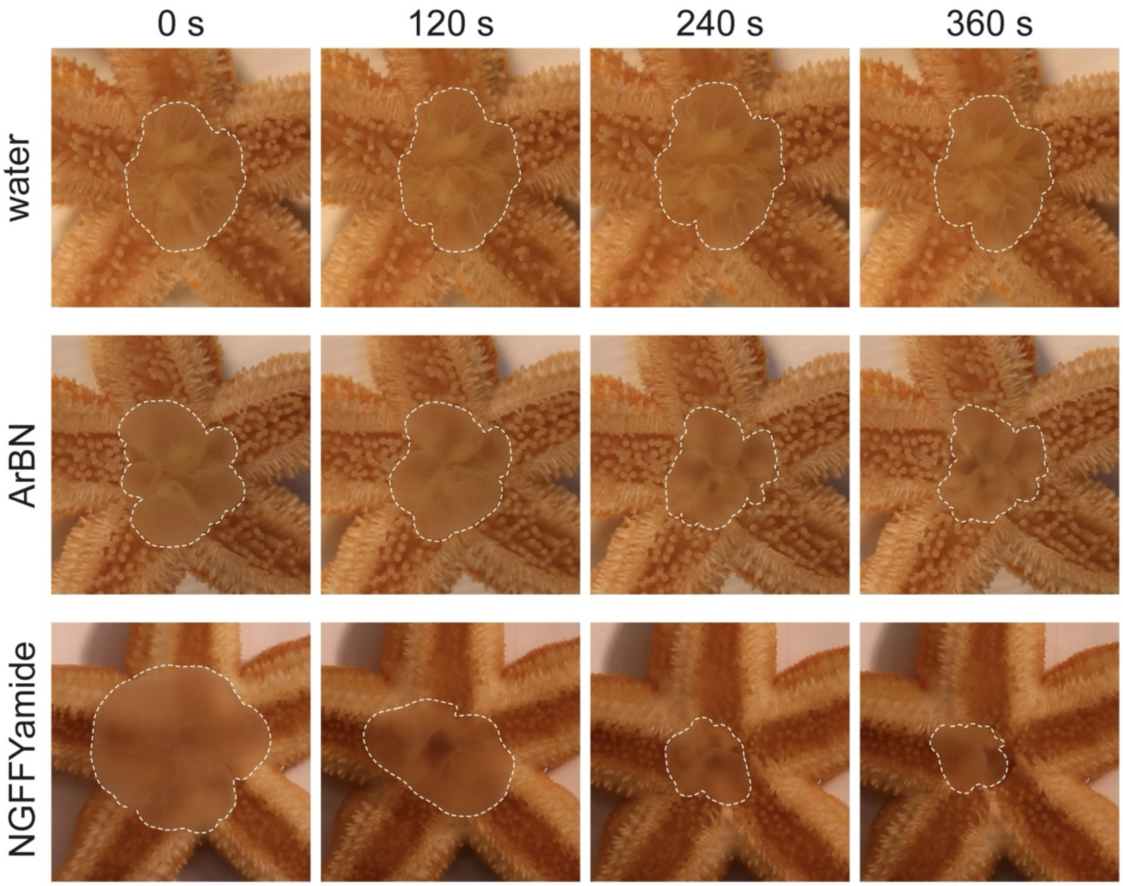

Fig. S13. (A) Photographs of representative *in vivo* experiments showing that ArBN and NGFFYamide, but not water (control), trigger cardiac stomach retraction in *A. rubens*. The two-dimensional area (marked by white dashed lines) of the everted cardiac stomach is progressively reduced at 120, 240 and 360 s after injection (0 s) of 10 µl ArBN (10^{-4} M) or 10 µl NGFFYamide (10^{-4} M; positive control) but not with water (negative control). See Fig. 7B for data from multiple experiments.

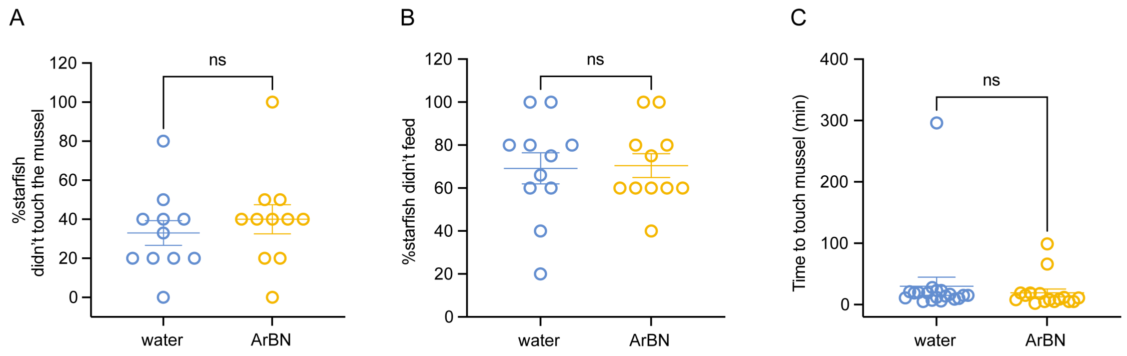

Fig. S14. Testing if ArBN affects feeding behavior in *A. rubens.* (A) There is not a statistically significant difference (P > 0.05) in the percentage of starfish that didn't touch the mussel in the control group (water-injection) and in the ArBN-injection group. (B) There is not a statistically significant difference (P > 0.05) in the percentage of the starfish that didn't feed on the mussel in the control group (water-injection) and in the ArBN-injection group. (C) There is not a statistically significant difference (P > 0.05) in the time taken for starfish to first touch the mussel when comparing the control group (water-injection) and the ArBN-injection group. (A and B) Data were analysed statistically using a Wilcoxon test in Prism 10 and shown as scatter plots. (C) Data were analysed statistically using a two-tailed Student's *t*-test in Prism 10 and shown as scatter plots.

A

ArBNR2

MANAYTVNTTAAMLGLVNSTTAPFFEVEDDIGGSPLFVMRVFMMIMIAVGVLGNGCVIFIV
LWNPSMRNRPNALVASLAIGDLLLLIICVPLKVYHMTYGQWPFGLLVCKLSNGFVIISQAVS
IFSMVALSHDRYRAIVTPMNYPRNRGSGYTIAVISVIWVVAILLACPTIVLSHIRGSDLFSFCL
YIHHTTLAGRLHACLRGLILFVIPLLVINGYYCLVSRKLIISTKALPGEVHDKRSQISARKRLA
YVVLAIVILFTVCWLPFTVIEVMYQINDQIFAESVGLTYLKLFSDFLTYVNSSANPILIIVISST
YREYFCSYIFCRCSGDLQYKRARTSTISSKASSTRTRMTEFA

Protter of ArBNR2

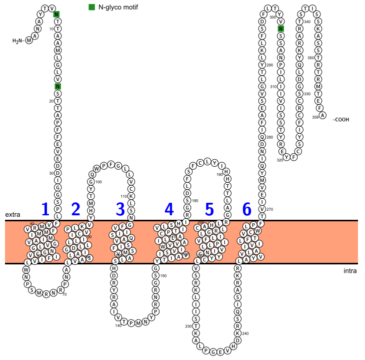

B

AaBNR2

LSDYSDLHNMANAYTVNTTAAMLGLVNSTTAPFFEVEDDIGGSPLFVMRVFMMIMIAVGV
LGNGCVIFIVLWNPSMRNRPNALVASLAIGDLLLLIICVPLKVYHMTYGQWPFGLLVCKLS
NGFVIISQAVSIFSMVALSHDRYRAIVTPMNYPRNRGSGYTIAVISVIWVVAILLACPTIVLS
HIRGSDLFSFCLYIHHTTLAGRLHACLRGLILFVIPLLVINGYYCLVSRKLIISTKALPGEVH
DKRSQISARKRLAYVVLAIVILFTVCWLPFTVIEVMYQINDQIFAESVGLTYLKLFSDFLTY
VNSSANPILIIVISSTYREYFCSYIFCRCSGDLQYKRARTSMISSKASSTRTRMTEFA

Protter of AaBNR2

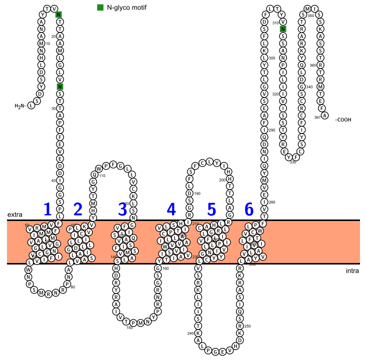

C

MgBNR2

MANPYTVNTTAAMLGLVNSTTAPFFEVEDDIGGSSLFTMKVLMIIMIVVGFLGNGCVIFIV
LWNPSMRNRPNALVASLAIGDLLLLIVCVPLKVYHMTYGQWPFGLFVCKLSNGLVIISQA
VSIFSMVALSHDRYRAIVTPMNYPRSRGSGSTIAVISVIWVVAILLACPTIVLSHIRGSELF
SFCLYIHHTTLAGRLHACLRGLILFVIPLLVINGYYCLVSRKLIISTKALPGEVHDKRSQISA
RKRLAYVVLAIVILFTVCWFPFTVIGVMYQFNDNMFAESVGLTYLKLFSDFLTYVNSSAN
PILIIVISSTYRKYFCSYIFCKCSGDLQYKRPRTSTISSKASSTRTRMTEFA

Protter of MgBNR2

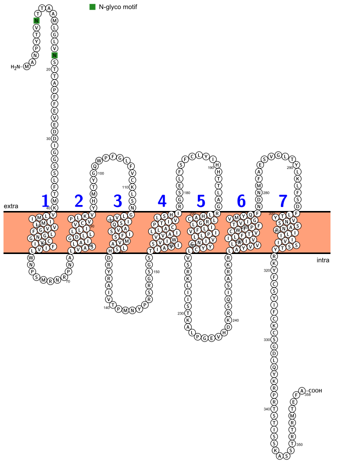

D

ArBNR2(G_{272})

MANAYTVNTTAAMLGLVNSTTAPFFEVEDDIGGSPLFVMRVFMMIMIAVGVLGNGCVIFIV
LWNPSMRNRPNALVASLAIGDLLLLIICVPLKVYHMTYGQWPFGLLVCKLSNGFVIISQAVS
IFSMVALSHDRYRAIVTPMNYPRNRGSGYTIAVISVIWVVAILLACPTIVLSHIRGSDLFSFCL
YIHHTTLAGRLHACLRGLILFVIPLLVINGYYCLVSRKLIISTKALPGEVHDKRSQISARKRLA
YVVLAIVILFTVCWLPFTVIGVMYQINDQIFAESVGLTYLKLFSDFLTYVNSSANPILIIVISST
YREYFCSYIFCRCSGDLQYKRARTSTISSKASSTRTRMTEFA

Protter of ArBNR2(G_{272})

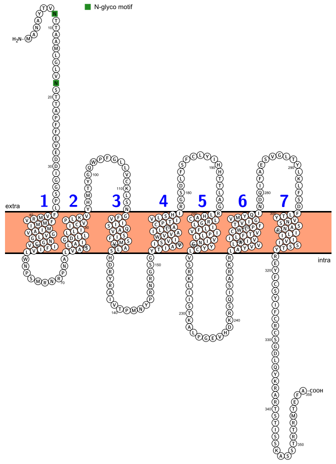

Fig. S15. Amino acid sequences and predicted topologies of *A. rubens* BNR2 (ArBNR2), *A. amurensis* BNR2 (AaBNR2) and *Marthasterias glacialis* BNR2 (MgBNR2). (A) Amino acid sequence and predicted topology of ArBNR2 (XP_033631283.1). Six predicted transmembrane domains are numbered successively in blue and predicted N-glycosylation sites are highlighted in green. The glutamate (E) at position 272 is highlighted in blue in the sequence. (B) Amino acid sequence and predicted topology of AaBNR2. Six predicted transmembrane domains are numbered successively in blue and predicted N-glycosylation sites are highlighted in green. The glutamate (E) at position 272 is highlighted in blue in the sequence. (C) Amino acid sequence and predicted topology of MgBNR2. Seven predicted transmembrane domains are numbered successively in blue and predicted N-glycosylation sites are highlighted in green. The glycine (G) at position 272 is highlighted in orange in the sequence. (D) Amino acid sequence and predicted topology of ArBNR2(G_{272}). Seven predicted transmembrane domains are numbered successively in blue and predicted N-glycosylation sites are highlighted in green. The glycine (G) at position 272 is highlighted in orange.

```
(A) >Spur_BNR1
atggatgcagtgagctattctcgggtagggagtacggccttcccgatgtctcctggaaaccaattcaatgtggcc
cttgggaactatagcgacggtaattataccgatcatgatatcccaccactgatgttccgggactacttcgagatg
ttcgtgttggtggtgataggtatctgcggtgtggtcggcaacggaacattgatatacagtgtactaatgaacaaa
gacatgaggagtgtccccaacgtcctcatagccaatgtggccgtcggtgacctccttgtgctgtcgttctcggtg
ccgttgaccgtcttatcctacatccaggacgatttccctctgggagcgaccatgtgcaaggtccaagcgttcatc
ccgattgtttctgaaggtgtctctgtgttcacgtttacggcgctcagcttcgaccgttacaacgccatcgtccgc
cccatacagcggcgacgaagccccgttgtccgtcgcacctgcgtggtggctgtctgtatctgggtcgtagccgta
tgccttgggataccctccatgtttcttgccttcctcagctacgagatgaaaccctatgtgctctgcttcattcta
ccccacttcaccctccaagcacgcatccacgaggtcacccgatgccttctcatgtacgttatcccactcaccatc
atcacttgttattactgcctcatcgcaatccagctctttcaaagttccagggacatgcccggtgaaggtcaccag
gagagcaagcaggcaagggctaggagacgtctcgccaaggctgttcttgtattggtctttatctttggtatctgc
tggtttccgcacttcatgagaagtctatggttccagttcggctacaacgagctactattcactaaaacagggatg
atgatattccaaagcgcatcccactttcttgtctacttcaactgcagcgccaaccctatcgctctctgttttcta
agtcggacctatcggcagtacttcatgaagtacctgtgctgtaaaagcggtcgtgagtttcgcggcccaatcaga
tttcaaaagaagtcgaccttcccactcaacccaagcaccaccaagaactcggctctggacgagtcgttgcggaca
tcttcggctgagacgaaaacatgcacggtttga

(B) >Spur_BNR1
MDAVSYSRVGSTAFPMSPGNQFNVALGNYSDGNYTDHDIPPLMFRDYFEMFVLVVIGICGVVGNGTLIYSVLMNK
DMRSVPNVLIANVAVGDLLVLSFSVPLTVLSYIQDDFPLGATMCKVQAFIPIVSEGVSVFTFTALSFDRYNAIVR
PIQRRRSPVVRRTCVVAVCIWVVAVCLGIPSMFLAFLSYEMKPYVLCFILPHFTLQARIHEVTRCLLMYVIPLTI
ITCYYCLIAIQLFQSSRDMPGEGHQESKQARARRRLAKAVLVLVFIFGICWFPHFMRSLWFQFGYNELLFTKTGM
MIFQSASHFLVYFNCSANPIALCFLSRTYRQYFMKYLCCKSGREFRGPIRFQKKSTFPLNPSTTKNSALDESLRT
SSAETKTCTV

(C) >Ajap_BNR1
atggagtcagtaataccgtatgaagttactactgtgcctggtatgaacgccacatcgtcttatgagtacgagtta
tacgcgataggatggaagacatatttagaggttgtattcatgtgtttttcggcgcagttggtaccattggtaat
ggtacgctcatactaattgtactgttaaatgctgatatgcgtaatgtacctaacacactaattgctagcctagcc
ttgggagatttcgtatttctcgcatggaatgtccctttcacaattgcatcgtattttcaccacacctatccattt
agtatgttcgtttgtaaaatgagttccgccatcccgattctctcggaaggtgtttctgtgttgacattaggggca
ctaagctttgaccgatataacgcgatcgtgcggcctatgcatcgtcgccgtagcaacgcagtcgtacagacgtac
cttttagcgttaattatttgggtagtggcgaccgctctctccgttcccagcttcataatcgcaaagagagtcgat
gtcgggatccctctttgttattcgctgccgttcggacaacccatctcgatcgttcacgagatatttcggtttttg
gtgctttacgttttcccgctcatcgtcatttcgcttttctacactctcatcgcactgcagctactggctagctca
cgtgacatgcctggagagagtcgtggtgacgcaaccaaacaagtgaaagcacgacgccgattggcccggtctgtc
atcgttctggtggccctctttgcaatatgctggtttccccatttcgtttatcagttcatatttcagttcaagttc
agtttggaagaagaggatttggcgttccttggaagccttcccatgagcattcttcaaactgtgtcacataatctg
atttacataaattctagtatcaaccctattgccatgtgctttctcagccagacgtaccgcagttatttctcgcat
tatctctgctactgtataccatcgaagagacgagagccagcacagagaggaagaatgagcaagaacgggacggct
ggaaataattccaaatggacaactgaaggatcaatgcccccatcggcggtggaaacgaaaacatgcgttttgtaa

(D) >Ajap_BNR1
MESVIPYEVTTVPGMNATSSYEYELYAIGWKTYLEVVFMCFFGAVGTIGNGTLILIVLLNADMRNVPNTLIASLA
LGDFVFLAWNVPFTIASYFHHTYPFSMFVCKMSSAIPILSEGVSVLTLGALSFDRYNAIVRPMHRRRSNAVVQTY
LLALIIWVVATALSVPSFIIAKRVDVGIPLCYSLPFGQPISIVHEIFRFLVLYVFPLIVISLFYTLIALQLLASS
RDMPGESRGDATKQVKARRRLARSVIVLVALFAICWFPHFVYQFIFQFKFSLEEEDLAFLGSLPMSILQTVSHNL
IYINSSINPIAMCFLSQTYRSYFSHYLCYCIPSKRREPAQRGRMSKNGTAGNNSKWTTEGSMPPSAVETKTCVL
```

Fig. S16. Sequences of Spur_BNR1 and Ajap_BNR1. (A) Nucleotide sequence encoding the BN-type receptor 1 in the sea urchin *Strongylocentrotus purpuratus* (Spur_BNR1). (B) Amino acid sequence of Spur_BNR1 (C) Nucleotide sequence encoding the BN-type receptor 1 in the sea cucumber *Apostichopus japonicus* (Ajap_BNR1). (D) Amino acid sequence of Ajap_BNR1.

Non-codon optimized sequence of ArBNR2

GGATCCGCCACC
ATGGCCAATGCTTATACAGTAAACACCACCGCTGCCATGCTTGGTCTAGTCAACAGCACCAC
AGCCCCATTCTTCGAAGTCGAAGACGACATCGGAGGGAGCCCTCTCTTCGTCATGCGGGTCT
TTATGATGATCATGATCGCAGTTGGGGTTCTCGGCAATGGCTGCGTCATCTTCATCGTCCTG
TGGAACCCTTCCATGCGCAACCGGCCCAACGCCCTCGTCGCCAGCCTTGCCATCGGCGATCT
GCTACTCCTGATCATCTGCGTACCTCTCAAGGTCTACCATATGACCTATGGACAGTGGCCCT
TCGGTCTCCTGGTCTGTAAACTCTCCAATGGCTTCGTCATCATCTCTCAGGCAGTGTCAATT
TTCTCCATGGTGGCGCTGAGCCATGACAGGTACCGGGCGATTGTCACACCCATGAACTACCC
TCGCAATCGTGGATCAGGCTACACCATCGCTGTCATATCCGTCATCTGGGTTGTGGCTATAC
TCCTAGCCTGCCCGACAATCGTCCTGTCCCACATCAGAGGCTCTGACTTGTTCTCATTCTGC
TTGTACATCCACCACACGACCTTGGCAGGGAGGCTCCACGCATGCTTGAGAGGCTTGATCCT
CTTCGTCATTCCTTTACTGGTCATTAATGGCTACTACTGCCTGGTGTCGCGGAAGCTGATCA
TCAGCACTAAGGCCCTCCCCGGTGAAGTCCACGATAAGAGGTCTCAGATAAGCGCCAGGAAG
CGCTTAGCATATGTGGTCCTGGCCATCGTGATCCTCTTCACAGTGTGCTGGCTTCCGTTCAC
CGTCATCGAGGTGATGTACCAGATCAACGACCAGATCTTCGCCGAGTCCGTCGGCCTGACCT
ACCTGAAGCTCTTCTCGGACTTCCTGACCTACGTCAACTCCAGCGCCAACCCAATTCTGATC
ATCGTCATCAGCTCAACCTACAGAGAGTACTTCTGCTCTTACATCTTCTGTCGGTGTTCTGG
GGATTTACAATATAAGAGAGCGCGCACATCAACGATATCGTCGAAAGCTTCATCTACGCGAA
CGAGAATGACTGAGTTTGCCTGA
CTCGAG

Optimized Sequence of ArBNR2:

GGATCCGCCACC
ATGGCAAATGCATATACTGTGAATACTACTGCCGCCATGCTGGGACTGGTCAATTCCACCAC
CGCTCCATTCTTCGAAGTGGAAGATGACATCGGCGGCTCCCCTCTGTTCGTGATGAGAGTCT
TCATGATGATCATGATCGCTGTGGGCGTGCTAGGCAACGGCTGCGTGATCTTCATCGTGCTG
TGGAACCCTTCTATGAGAAACAGACCTAACGCCCTGGTGGCCTCCCTGGCTATCGGCGACCT
GCTGCTGCTGATCATCTGCGTGCCCCTGAAAGTGTACCACATGACCTACGGCCAGTGGCCTT
TCGGCCTGCTCGTGTGCAAGCTGTCCAATGGCTTCGTGATCATCTCTCAGGCCGTGTCCATC
TTCTCCATGGTGGCTCTGAGCCATGACAGATACCGAGCCATCGTGACCCCTATGAACTACCC
TCGGAACCGGGGATCTGGATACACCATCGCCGTGATCTCTGTGATCTGGGTCGTGGCTATCC
TGTTGGCTTGTCCTACCATCGTGCTGTCTCACATCAGAGGCTCTGACCTGTTCTCTTTCTGT
CTGTACATCCACCACACAACCCTGGCTGGCAGACTGCACGCCTGCCTGAGAGGCCTCATCCT
GTTTGTGATCCCTCTGCTGGTGATCAACGGTTACTACTGCCTGGTTTCTCGGAAACTGATCA
TCAGCACCAAGGCACTGCCTGGCGAGGTGCACGACAAGCGGTCCCAAATTTCCGCCCGGAAG
CGCCTGGCCTACGTGGTGCTGGCTATCGTCATCCTGTTTACCGTGTGTTGGCTGCCCTTCAC
CGTGATCGAGGTCATGTACCAGATCAACGACCAGATCTTCGCCGAGTCCGTGGGCCTGACAT
ACCTGAAGCTGTTTTCCGACTTCCTGACCTATGTGAACAGCTCTGCCAACCCCATCCTGATC
ATCGTGATCAGCTCCACCTATAGAGAGTACTTCTGCTCCTACATCTTTTGCAGATGCAGTGG
CGATCTGCAGTACAAGAGAGCCCGGACCTCTACCATCTCCTCCAAGGCCTCTAGCACCAGAA
CACGGATGACAGAGTTCGCTTGA
CTCGAG

Non-codon optimized sequence of ArBNR2 (G_{272}):
GGATCCGCCACC
ATGGCCAATGCTTATACAGTAAACACCACCGCTGCCATGCTTGGTCTAGTCAACAGCACCAC
AGCCCCATTCTTCGAAGTCGAAGACGACATCGGAGGGAGCCCTCTCTTCGTCATGCGGGTCT
TTATGATGATCATGATCGCAGTTGGGGTTCTCGGCAATGGCTGCGTCATCTTCATCGTCCTG
TGGAACCCTTCCATGCGCAACCGGCCCAACGCCCTCGTCGCCAGCCTTGCCATCGGCGATCT
GCTACTCCTGATCATCTGCGTACCTCTCAAGGTCTACCATATGACCTATGGACAGTGGCCCT
TCGGTCTCCTGGTCTGTAAACTCTCCAATGGCTTCGTCATCATCTCTCAGGCAGTGTCAATT
TTCTCCATGGTGGCGCTGAGCCATGACAGGTACCGGGCGATTGTCACACCCATGAACTACCC
TCGCAATCGTGGATCAGGCTACACCATCGCTGTCATATCCGTCATCTGGGTTGTGGCTATAC
TCCTAGCCTGCCCGACAATCGTCCTGTCCCACATCAGAGGCTCTGACTTGTTCTCATTCTGC
TTGTACATCCACCACACGACCTTGGCAGGGAGGCTCCACGCATGCTTGAGAGGCTTGATCCT
CTTCGTCATTCCTTTACTGGTCATTAATGGCTACTACTGCCTGGTGTCGCGGAAGCTGATCA
TCAGCACTAAGGCCCTCCCCGGTGAAGTCCACGATAAGAGGTCTCAGATAAGCGCCAGGAAG
CGCTTAGCATATGTGGTCCTGGCCATCGTGATCCTCTTCACAGTGTGCTGGCTTCCGTTCAC
CGTCATCGGGGTGATGTACCAGATCAACGACCAGATCTTCGCCGAGTCCGTCGGCCTGACCT
ACCTGAAGCTCTTCTCGGACTTCCTGACCTACGTCAACTCCAGCGCCAACCCAATTCTGATC
ATCGTCATCAGCTCAACCTACAGAGAGTACTTCTGCTCTTACATCTTCTGTCGGTGTTCTGG
GGATTTACAATATAAGAGAGCGCGCACATCAACGATATCGTCGAAAGCTTCATCTACGCGAA
CGAGAATGACTGAGTTTGCCTGA
CTCGAG

Optimized Sequence of ArBNR2 (G_{272}):
GGATCCGCCACC
ATGGCAAATGCATATACTGTGAATACTACTGCCGCCATGCTGGGCCTGGTCAATTCCACCAC
CGCTCCCTTCTTCGAGGTGGAAGATGACATCGGCGGCTCTCCACTGTTTGTGATGAGAGTTT
TCATGATGATCATGATCGCTGTGGGCGTGCTGGGCAACGGCTGCGTTATCTTCATCGTGCTG
TGGAACCCCTCTATGCGGAACAGACCTAACGCCCTGGTGGCCTCTCTGGCTATCGGAGATCT
GCTGCTGCTCATCATCTGCGTGCCTCTGAAAGTGTACCACATGACCTACGGCCAGTGGCCTT
TCGGCCTGCTGGTGTGCAAGCTGTCCAACGGCTTCGTGATCATCTCTCAGGCCGTGTCCATC
TTCTCCATGGTGGCCCTGTCTCATGACAGATACAGAGCTATTGTGACCCCTATGAACTATCC
TAGGAACCGGGGAAGCGGCTATACAATCGCCGTGATCTCTGTGATCTGGGTCGTGGCTATCC
TGCTCGCCTGTCCTACCATCGTGCTGTCCCACATCAGAGGCTCTGATCTGTTTAGCTTCTGC
CTGTACATCCACCACACCACTCTGGCTGGCAGACTGCACGCCTGTCTGAGAGGCCTGATCCT
GTTCGTGATCCCTCTGCTGGTGATCAATGGCTACTACTGCCTGGTGTCCCGGAAGCTGATCA
TCAGCACCAAGGCTCTGCCTGGCGAAGTGCACGACAAGAGAAGCCAAATCTCCGCTCGGAAA
CGCCTTGCCTACGTGGTGCTGGCTATCGTGATCCTGTTCACCGTGTGCTGGCTGCCCTTCAC
CGTGATCGGAGTGATGTACCAGATCAACGACCAGATCTTTGCCGAGTCCGTGGGTCTGACCT
ACTTGAAGCTGTTTTCTGACTTCCTGACCTACGTGAACTCCTCCGCCAACCCTATCCTGATC
ATCGTCATTTCCTCTACATACCGGGAGTACTTCTGCTCCTACATCTTCTGCAGATGTTCTGG
CGACCTGCAGTACAAGCGGGCCAGAACCTCTACCATCTCCTCCAAGGCCTCCTCTACCAGAA
CACGGATGACCGAGTTCGCTTGA
CTCGAG

Fig. S17. The non-codon optimized and codon-optimized sequences of cDNAs encoding ArBNR2 and ArBNR2(G_{272}). The partial Kozak sequence (GCCACC) added before the ATG start codon is shown in underlined text. The BamHI enzyme cut site is shown in blue. The XhoI enzyme cut site is shown in green.

Table S1. List of GenBank accession numbers used for sequence alignment of neuropeptides derived from BN-type precursors in Figure 1A.

**Species name**	**Neuropeptide name**	**Accession number**
Asterias rubens	Bombesin (BN)	XP_033624725.1
Acanthaster cf. *solaris*	Bombesin (BN)	XP_022087127.1
Ophionotus victoriae	Bombesin (BN)	O_vic_ts_scaffold51173 (PMID: 25699014)
Strongylocentrotus purpuratus	Bombesin (BN)	XP_030829469.1
Apostichopus japonicus	Bombesin (BN)	GHCH01035201.1; GHCH01064199.1: https://www.ncbi.nlm.nih.gov/Traces/wgs/GHCH01 (PMID: 31222106)
Branchiostoma floridae	Gastrin-releasing peptide (GRP)	XP_035670839.1
Bombina bombina	Bombesin (BN)	710343B
Rana pipiens	Ranatensin (RN)	701177B
Phyllomedusa sauvagii	Phyllolitorin (PN)	0910155A
Lepisosteus oculatus	Gastrin-releasing peptide (GRP)	XP_015194936.1
Gallus gallus	Gastrin-releasing peptide (GRP)	NP_001264829.1
Homo sapiens	Gastrin-releasing peptide (GRP)	NP_001012531.1
Lepisosteus oculatus	Neuromedin B (NMB)	XP_015198865.1
Gallus gallus	Neuromedin B (NMB)	NP_001072944.1
Homo sapiens	Neuromedin B (NMB)	NP_066563.2

Table S2. List of accession numbers used for phylogenetic analysis of BN-type, ET-type, CCHa/EP-type peptide precursors in different taxa for Figure 1B.

**Species name**	**Neuropeptide name**	**Accession number**
Asterias rubens	Bombesin (BN)	XP_033624725.1
Acanthaster cf. *solaris*	Bombesin (BN)	XP_022087127.1
Strongylocentrotus purpuratus	Bombesin (BN)	XP_030829469.1
Apostichopus japonicus	Bombesin (BN)	GHCH01035201.1; GHCH01064199.1: https://www.ncbi.nlm.nih.gov/Traces/wgs/GHCH01 (PMID: 31222106)
Bombina bombina	Bombesin	P21591.1
Rana pipiens	Ranatensin	P08950.1
Phyllomedusa sauvagii	Phyllolitorin	P08948.4
Branchiostoma floridae	Gastrin-releasing peptide (GRP)	XP_035670839.1
Lepisosteus oculatus	Gastrin-releasing peptide (GRP)	XP_015194936.1
Latimeria chalumnae	Gastrin-releasing peptide (GRP)	XP_014349884.1
Gallus gallus	Gastrin-releasing peptide (GRP)	NP_001264829.1
Homo sapiens	Gastrin-releasing peptide (GRP)	NP_001012531.1
Lepisosteus oculatus	Neuromedin B (NMB)	XP_015198865.1
Latimeria chalumnae	Neuromedin B (NMB)	XP_014343687.1
Gallus gallus	Neuromedin B (NMB)	NP_001072944.1
Homo sapiens	Neuromedin B (NMB)	NP_066563.2
Drosophila melanogaster	CCHamide (CCHa1)	NP_001097784.1
Drosophila melanogaster	CCHamide (CCHa2)	NP_001189216.1
Bombyx mori	CCHamide (CCHa)	NP_001123587.1
Eupeodes corollae	CCHamide (CCHa)	XP_055904908.1
Tribolium castaneum	CCHamide (CCHa)	NP_001280542.1
Haliotis rufescens	excitatory peptide (EP)	XP_046370918.1
Lineus longissimus	excitatory peptide (EP)	QDS02609.1
Perinereis vancaurica	excitatory peptide (EP)	BAB83127.1
Mytilus californianus	excitatory peptide (EP)	XP_052087808.1
Gigantopelta aegis	excitatory peptide (EP)	XP_041354247.1
Homo sapiens	endothelin (ET)	NP_001403492.1
Gallus gallus	endothelin (ET)	XP_040520547.1
Latimeria chalumnae	endothelin (ET)	XP_005995271.1
Lepisosteus oculatus	endothelin (ET)	XP_006635920.1
Rana temporaria	endothelin (ET)	XP_040209666.1
Gigantopelta aegis	elevenin (ELev)	XP_041367527.1
Haliotis rufescens	elevenin (ELev)	XP_046329820.1

Charonia tritonis	elevenin (ELev)	AQS80540.1
Conus ebraeus	elevenin (ELev)	UMA82997.1
Conus magus	elevenin (ELev)	WEQ50374.1

Table S3. List of accession numbers of the precursor cDNAs and corresponding genomic sequences used for gene structure comparison in Figure 2.

**Species name**	**Neuropeptide name**	**Transcripts**	**Genome**
Asterias rubens	Bombesin (BN)	XM_033768834.1	NC_047064.1
Acanthaster cf. *solaris*	Bombesin (BN)	XM_022231435.1	NW_019091389.1
Strongylocentrotus purpuratus	Bombesin (BN)	XM_011680527.2	NW_022145544.1
Apostichopus japonicus	Bombesin (BN)	GHCH01035201.1; GHCH01064199.1: https://www.ncbi.nlm.nih.gov/Traces/wgs/GHCH01?display=contigs (PMID: 31222106)	MRZV01000438.1
Branchiostoma floridae	Gastrin-releasing peptide (GRP)	XM_035814946.1	NC_049981.1
Lepisosteus oculatus	Gastrin-releasing peptide (GRP)	XM_015339450.1	NC_023180.1
Gallus gallus	Gastrin-releasing peptide (GRP)	NM_001277900.1	Whole genome accessible with NCBI SPLIGN tool
Homo sapiens	Gastrin-releasing peptide (GRP)	NM_001012512.3	Whole genome accessible with NCBI SPLIGN tool
Lepisosteus oculatus	Neuromedin B (NMB)	XM_015343379.1	NC_023181.1
Gallus gallus	Neuromedin B (NMB)	NM_001079476.3	Whole genome accessible with NCBI SPLIGN tool
Homo sapiens	Neuromedin B (NMB)	NM_021077.4	Whole genome accessible with NCBI SPLIGN tool

Table S4. List of sequences used for CLANS/phylogenetic analysis of BN/ET/CCHa/EP-type receptors.

Species name	Receptor name	Accession number
Asterias rubens	Bombesin-type receptor (BNR1)	XP_033635529.1
Asterias rubens	Bombesin-type receptor (BNR2)	XP_033631283.1
Asterias rubens	Bombesin-type receptor (BNR3)	XP_033634790.1
Asterias rubens	Bombesin-type receptor (BNR4)	XP_033640596.1
Asterias rubens	Bombesin-type receptor (BNR5)	XP_033640355.1
Asterias rubens	Bombesin-type receptor (BNR6)	XP_033640356.1
Asterias rubens	Bombesin-type receptor (BNR7)	XP_033625054.1
Asterias rubens	Bombesin-type receptor (BNR8)	XP_033631320.1
Asterias rubens	Bombesin-type receptor (BNR9)	XP_033624587.1
Asterias rubens	Bombesin-type receptor (BNR10)	XP_033637574.1
Asterias rubens	Bombesin-type receptor (BNR11)	XP_033629579.1
Acanthaster cf. *solaris*	Bombesin-type receptor (BNR1)	XP_022082131.1
Acanthaster cf. *solaris*	Bombesin-type receptor (BNR)	XP_022079658.1
Acanthaster cf. *solaris*	Bombesin-type receptor (BNR)	XP_022100897.1
Acanthaster cf. *solaris*	Bombesin-type receptor (BNR)	XP_022088892.1
Acanthaster cf. *solaris*	Bombesin-type receptor (BNR)	XP_022084711.1
Acanthaster cf. *solaris*	Bombesin-type receptor (BNR)	XP_022079648.1
Acanthaster cf. *solaris*	Bombesin-type receptor (BNR)	XP_022091148.1
Acanthaster cf. *solaris*	Bombesin-type receptor (BNR)	XP_022093483.1
Acanthaster cf. *solaris*	Bombesin-type receptor (BNR)	XP_022083484.1
Acanthaster cf. *solaris*	Bombesin-type receptor (BNR)	XP_022111775.1
Acanthaster cf. *solaris*	Bombesin-type receptor (BNR)	XP_022106504.1
Apostichopus japonicus	Bombesin-type receptor (BNR1)	PIK58426.1
Apostichopus japonicus	Bombesin-type receptor (BNR)	PIK52576.1
Apostichopus japonicus	Bombesin-type receptor (BNR)	PIK36207.1
Apostichopus japonicus	Bombesin-type receptor (BNR)	PIK48518.1
Apostichopus japonicus	Bombesin-type receptor (BNR)	PIK59471.1
Strongylocentrotus purpuratus	Bombesin-type receptor (BNR1)	XP_785425.3
Strongylocentrotus purpuratus	Bombesin-type receptor (BNR)	XP_030836709.1
Strongylocentrotus purpuratus	Bombesin-type receptor (BNR)	XP_011662370.1
Strongylocentrotus purpuratus	Bombesin-type receptor (BNR)	XP_011669248.2
Strongylocentrotus purpuratus	Bombesin-type receptor (BNR)	XP_030837089.1
Strongylocentrotus purpuratus	Bombesin-type receptor (BNR)	XP_003725585.3
Strongylocentrotus purpuratus	Bombesin-type receptor (BNR)	XP_003724609.1
Strongylocentrotus purpuratus	Bombesin-type receptor (BNR)	XP_030830704.1

Strongylocentrotus purpuratus	Bombesin-type receptor (BNR)	XP_003731093.1
Saccoglossus kowalevskii	Bombesin-type receptor (BNR1)	XP_002731022.1
Saccoglossus kowalevskii	Bombesin-type receptor (BNR2)	XP_002731023.1
Branchiostoma floridae	Gastrin-releasing peptide receptor (GRPR1)	XP_035687477.1
Branchiostoma floridae	Gastrin-releasing peptide receptor (GRPR2)	XP_035687654.1
Branchiostoma floridae	Gastrin-releasing peptide receptor (GRPR3)	XP_035685542.1
Lepisosteus oculatus	Gastrin- releasing peptide receptor (GRPR)	XP_006639277.1
Gallus gallus	Gastrin- releasing peptide receptor (GRPR)	NP_989738.1
Homo sapiens	Gastrin- releasing peptide receptor (GRPR)	NP_005305.1
Homo sapiens	Bombesin receptor subtype-3 (BRS3)	NP_001718.1
Lepisosteus oculatus	Neuromedin B receptor (NMBR)	XP_006643031.1
Gallus gallus	Neuromedin B receptor (NMBR)	XP_426167.1
Homo sapiens	Neuromedin B receptor (NMBR)	NP_002502.2
Branchiostoma floridae	Endothelin receptor (ETR)	XP_035681520.1
Lepisosteus oculatus	Endothelin receptor (ETR)	XP_006629609.2
Gallus gallus	Endothelin receptor (ETR)	NP_989450.1
Homo sapiens	Endothelin receptor (ETA)	NP_001948.1
Homo sapiens	Endothelin receptor (ETB)	NP_000106.1
Bombyx mandarina	CCHamide receptor (CCHaR)	XP_028029274.1
Drosophila melanogaster	CCHamide receptor (CCHaR1)	NP_611241.2
Drosophila melanogaster	CCHamide receptor (CCHaR2)	NP_610199.2
Daphnia pulex	CCHamide receptor (CCHaR)	EFX87704.1
Platynereis dumerilii	excitatory peptide receptor (EPR)	AKQ63032.1
Capitella teleta	excitatory peptide receptor (EPR)	ELT88787.1
Lottia gigantea	excitatory peptide receptor (EPR)	XP_009065070.1
Gigantopelta aegis	excitatory peptide receptor (EPR)	XP_041354456.1
Platynereis dumerilii	elevenin receptor (ELevR)	AKQ63044.1
Lottia gigantea	elevenin receptor (ELevR)	XP_009045672.1
Gigantopelta aegis	elevenin receptor (ELevR)	XP_041357866.1
Aplysia californica	elevenin receptor (ELevR)	WHL35492.1
Lottia gigantea	orexin receptor (OXR1)	XP_009061187.1
Lottia gigantea	orexin receptor (OXR2)	XP_009057000.1
Capitella teleta	orexin receptor (OXR)	ELU18833.1
Strongylocentrotus purpuratus	orexin receptor (OXR)	XP_030833857.1
Anguilla anguilla	orexin receptor (OXR)	XP_035255752.1
Homo sapiens	orexin receptor (OXR1)	NP_001516.2
Homo sapiens	orexin receptor (OXR2)	NP_001371201.1

Table S5. Primers used to amplify cDNAs encoding ArBNR1 and ArBNR3.

Receptor (Contig)	Forward Primers (5' to 3')	Reverse Primers (5' to 3')	Annealing Temperature	Product Length
ArBNR1 (1114094)	ACAGTCACCATGAGTT CCCAGACAA	CATTGTGAATTCAAACA GTGCAT	63°C	1144 bp
ArBNR3 (1113802)	TCTACCATGCACGTACT TGGCA	CTAAGCTGGCCGTGGT GTAT	68°C	1159 bp

Dataset S1 (separate file). Data for functional characterization of putative *A. rubens* BN-type receptors (ArBNR1, ArBNR2, ArBNR2 (G_{272}) and ArBNR3).

Dataset S2 (separate file). Data for analysis of the *in vitro* effects of ArBN on cardiac stomach and tube foot preparations from *A. rubens.*

Dataset S3 (separate file). Data for analysis of the *in vivo* effects of ArBN on the everted cardiac stomach of *A. rubens*.

Dataset S4 (separate file). Data for analysis of the *in vivo* effects of ArBN on feeding behavior of *A. rubens*.

SI References

1. A. B. Chaet, A mechanism for obtaining mature gametes from starfish. *The Biological Bulletin* **126(1)**, 8-13 (1964).
2. M. Lin *et al.*, Cellular localization of relaxin-like gonad-stimulating peptide expression in Asterias rubens: New insights into neurohormonal control of spawning in starfish. *J Comp Neurol* **525**, 1599-1617 (2017).
3. F. Meier *et al.*, Online Parallel Accumulation-Serial Fragmentation (PASEF) with a Novel Trapped Ion Mobility Mass Spectrometer. *Mol Cell Proteomics* **17**, 2534-2545 (2018).
4. R. J. N. Qizhi Hu, Hongyan Li, Alexander Makarov, Mark Hardman and R. Graham Cooks, The Orbitrap: anew mass spectrometer. *Journal of Mass Spectrometry* **40**, 430-443 (2005).
5. D. Kessner, M. Chambers, R. Burke, D. Agus, P. Mallick, ProteoWizard: open source software for rapid proteomics tools development. *Bioinformatics* **24**, 2534-2536 (2008).
6. A. I. Nesvizhskii, A. Keller, E. Kolker, R. Aebersold, A statistical model for identifying proteins by tandem mass spectrometry. *Anal Chem* **75**, 4646-4658 (2003).
7. O. Mirabeau, J. S. Joly, Molecular evolution of peptidergic signaling systems in bilaterians. *Proc Natl Acad Sci U S A* **110**, E2028-2037 (2013).
8. C. Chen *et al.*, TBtools: An Integrative Toolkit Developed for Interactive Analyses of Big Biological Data. *Mol Plant* **13**, 1194-1202 (2020).
9. U. Omasits, C. H. Ahrens, S. Muller, B. Wollscheid, Protter: interactive protein feature visualization and integration with experimental proteomic data. *Bioinformatics* **30**, 884-886 (2014).
10. R. Breathnach, C. Benoist, K. O'Hare, F. Gannon, P. Chambon, Ovalbumin gene: evidence for a leader sequence in mRNA and DNA sequences at the exon-intron boundaries. *Proc Natl Acad Sci U S A* **75**, 4853-4857 (1978).
11. T. Frickey, A. Lupas, CLANS: a Java application for visualizing protein families based on pairwise similarity. *Bioinformatics* **20**, 3702-3704 (2004).
12. C. Chen *et al.*, TBtools-II: A "one for all, all for one" bioinformatics platform for biological big-data mining. *Mol Plant* **16**, 1733-1742 (2023).
13. V. Baubet *et al.*, Chimeric green fluorescent protein-aequorin as bioluminescent Ca2+ reporters at the single-cell level. *Proc Natl Acad Sci U S A* **97**, 7260-7265 (2000).
14. Y. Zhang *et al.*, Molecular and functional characterization of somatostatin-type signalling in a deuterostome invertebrate. *Open Biol* **10**, 200172 (2020).
15. J. B. Millar, E. Rozengurt, Bombesin enhancement of cAMP accumulation in Swiss 3T3 cells: evidence of a dual mechanism of action. *J Cell Physiol* **137**, 214-222 (1988).
16. S. Karlsson, B. Ahren, Gastrin-releasing peptide mobilizes calcium from intracellular stores in HIT-T15 cells. *Peptides* **17**, 909-916 (1996).
17. L. J. Garcia, T. K. Pradhan, H. C. Weber, T. W. Moody, R. T. Jensen, The gastrin-releasing peptide receptor is differentially coupled to adenylate cyclase and phospholipase C in different tissues. *Biochim Biophys Acta* **1356**, 343-354 (1997).
18. M. R. Hellmich *et al.*, Multiple protein kinase pathways are involved in gastrin-releasing peptide receptor-regulated secretion. *J Biol Chem* **274**, 23901-23909 (1999).

19. P. E. Squires, R. M. Meloche, A. M. Buchan, Bombesin-evoked gastrin release and calcium signaling in human antral G cells in culture. *Am J Physiol* **276**, G227-237 (1999).
20. B. R. Conklin *et al.*, Carboxyl-terminal mutations of Gq alpha and Gs alpha that alter the fidelity of receptor activation. *Mol Pharmacol* **50**, 885-890 (1996).
21. A. B. Tinoco, M. Egertova, M. R. Elphick, Immunohistochemical localisation of vasopressin/oxytocin-type, corazonin-type and luqin-type neuropeptide expression in the starfish Asterias rubens using antibodies to the C-terminal region of precursor proteins. *Cell Tissue Res* **391**, 441-456 (2023).
22. D. C. Semmens *et al.*, Discovery of a novel neurophysin-associated neuropeptide that triggers cardiac stomach contraction and retraction in starfish. *J Exp Biol* **216**, 4047-4053 (2013).
23. S. Tian, M. Egertova, M. R. Elphick, Functional Characterization of Paralogous Gonadotropin-Releasing Hormone-Type and Corazonin-Type Neuropeptides in an Echinoderm. *Front Endocrinol (Lausanne)* **8**, 259 (2017).
24. A. G. Mayer, On the use of magnesium in stupefying marine animals. *The Biological Bulletin* **17**, 341-342 (1909).
25. J. M. Lawrence, B. C. Cowell, The righting response as an indication of stress in stichaster striatus (Echinodermata, asteroidea). *Marine and Freshwater Behaviour and Physiology* **27**, 239-248 (1996).
26. A. B. Tinoco *et al.*, Characterization of NGFFYamide Signaling in Starfish Reveals Roles in Regulation of Feeding Behavior and Locomotory Systems. *Front Endocrinol (Lausanne)* **9**, 507 (2018).
27. C. Hummel, P. Honkoop, J. van der Meer, Small is profitable: No support for the optimal foraging theory in sea stars Asterias rubens foraging on the blue edible mussel Mytilus edulis. *Estuarine, Coastal and Shelf Science* **94**, 89-92 (2011).
28. M. J. Telford *et al.*, Phylogenomic analysis of echinoderm class relationships supports Asterozoa. *Proc Biol Sci* **281** (2014).
